# Synthesis of Chroman-2,4-diones via Ring-Opening/Ring-Closing Reaction Involving Palladium-Catalyzed Intramolecular Aryloxycarbonylation

**DOI:** 10.1021/acs.joc.3c02337

**Published:** 2024-01-09

**Authors:** Sami Chniti, Péter Pongrácz, László Kollár, Attila Bényei, Ágnes Dörnyei, Attila Takács

**Affiliations:** † Department of General and Inorganic Chemistry, Faculty of Sciences, University of Pécs, Ifjúság u. 6., Pécs H-7624, Hungary; ‡ János Szentágothai Research Centre, University of Pécs, Ifjúság u. 20., Pécs H-7624, Hungary; § HUN-REN-PTE Research Group for Selective Chemical Syntheses, Ifjúság u. 6., Pécs H-7624, Hungary; ∥ Department of Physical Chemistry, University of Debrecen, Egyetem tér 1., Debrecen H-4032, Hungary; ⊥ Department of Analytical and Environmental Chemistry, Faculty of Sciences, University of Pécs, Ifjúság u. 6., Pécs H-7624, Hungary

## Abstract

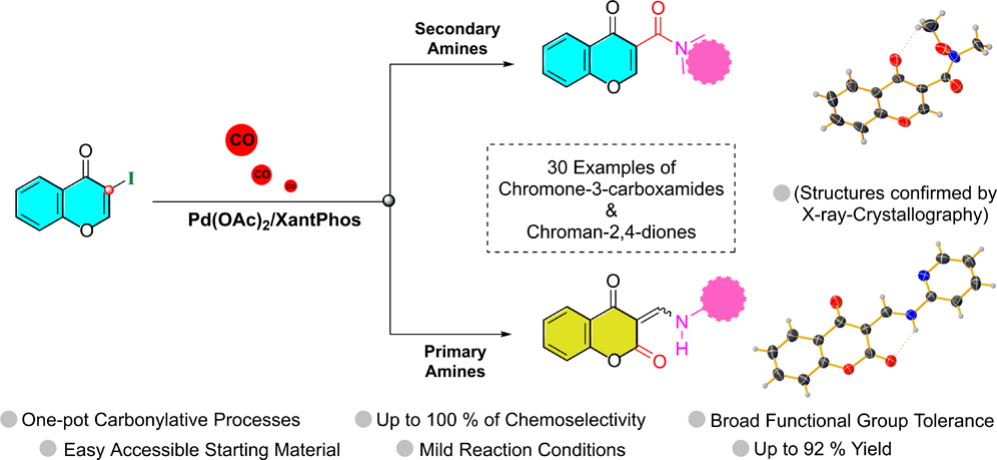

Palladium-catalyzed aminocarbonylation of 3-iodochromone was studied in the presence of primary and secondary amines using atmospheric pressure of carbon monoxide as a carbonyl source. This procedure successfully provided a library of chromone-3-carboxamides and 3-substituted chroman-2,4-diones in 40 to 92% isolated yields. The reaction proceeded via highly chemoselective aminocarbonylation (up to 100%) in the presence of secondary amines by using monodentate or bidentate phosphine ligands. The tendency of 3-iodochromone substrate to undergo ANRORC rearrangement with N-nucleophiles was crucial to shift the reaction toward an unprecedented chemoselective carbonylative transformation, where a late-stage carbonyl insertion is favored concomitantly to the last ring-closure step. The proposed aza-Michael addition/ring-opening/intramolecular aryloxycarbonylation sequence showed compatibility, uniquely, to primary amines when XantPhos was used as a ligand. The solid-state structures of chromone-3-carboxamide (2a) and chroman-2,4-dione (3s) were undoubtedly established by single-crystal XRD analysis. A catalytic cycle was proposed to rationalize the formation of the two types of carbonylated compounds.

## Introduction

Chromone (4H-1-benzopyran-4-one) is a ubiquitous heterocyclic core that constitutes the backbone of various vital compounds produced via biosynthetic pathways in plants.^1^ Several naturally occurring chromones and benzoannelated γ-pyrone-based rings, such as Diosmin, Apigenin, and Flavoxate, serve not only as crucial secondary metabolites throughout the plant’s life cycle but also as valid scaffolds in the design and discovery of original and potent drugs.^2^

The pharmacological profile of chromones and chromone hybrids is increasing by virtue of their ability to act as a privileged skeleton on many biological targets such as enzymes and receptors.^3^ In this context, a panoply of chromone derivatives, generally exhibiting very low mammalian toxicity,^4^ have been reported as antibacterial,^5^ antioxidant,^5^ anti-HIV,^6^ immune stimulators,^7^ and anticancer agents categorized as inhibitors of topoisomerases,^8^ protein kinases,^9^ A3 adenosine receptors,^10^ and as drug transporters.^11^

Different synthetic strategies have been implemented to access chromones including transition-metal mediated transformations involving iridium, ruthenium, and palladium catalysts, Vilsmeier–Haack reaction, Claisen condensation, Simonis reaction, Baker-Venkataraman rearrangement, and Kostanecki-Robinson reaction.^2^ Moreover, structure-activity-relationship (SAR) studies aiming to optimize the chromone nucleus have been carried out. Accordingly, the incorporation of a pharmacophore moiety into 2- and 3-positions or the aromatic substitution on the ring A could confer to the final chromone essential structural features and new biological properties, as in the case of chromone-based marketed drugs: Khellin, Rapitil, and Intal.^12^

Interestingly, many efforts have been made to install the amide unit into the chromone ring since combined theoretical and experimental assessments pointed out that the resulting framework could be a future human monamine oxidase-B inhibitor, a relevant candidate for the treatment of challenging neurodegenerative disorders such Alzheimer’s and Parkinson’s diseases.^13^

**Figure 1:**
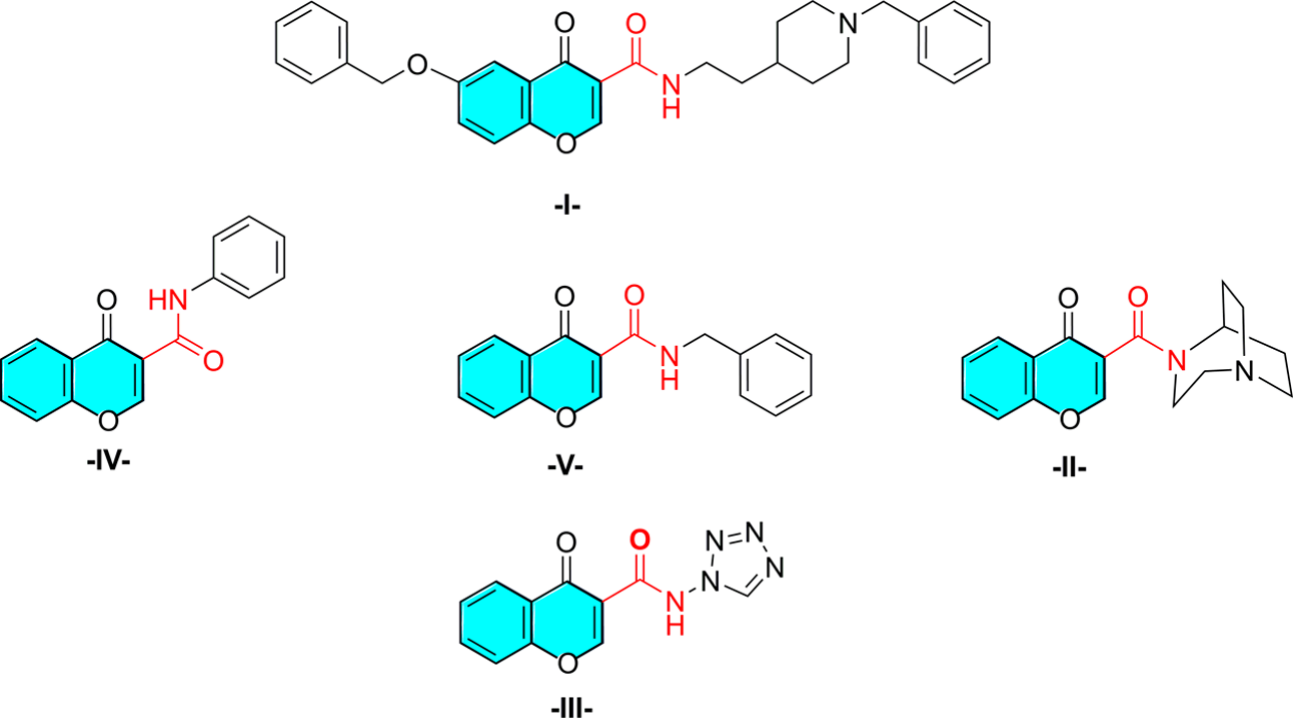
Important examples of secondary and tertiary chromone-3-carboxamide pharmaceuticals.

Analogously, with a superior inhibitory activity toward human MAO-B (Monoamine oxidase B) compared to chromone-2-carboxamide counterparts, tremendous examples of chromone-3-carboxamides (Figure 1) have been described as potent inhibitors of human acetylcholinesterase **I**,^14^ and as activators of defective or malfunctioning nicotinic acetylcholine receptors (nAChR), especially of the brain **II**.^15^ Furthermore, different structural analogs have been documented as antiallergic **III** for the management of passive cutaneous anaphylaxis,^16^ as cytotoxic **IV**,^17^ and as anti-inflammatory agents **V**.^17^

On the other hand, transition metal-catalyzed carbonylative cross-coupling reactions have emerged as a captivating powerful tool to introduce one or two carbonyl motif into aryl-, heteroaryl-, alkenyl halides, and alternative activated substrates, yielding new functionalities such as amides, ketoamides, carbamates, aldehydes, ketones, carboxylic acids, etc.^18^ Particularly, palladium-catalyzed aminocarbonylation as a highly chemoselective one-step transformation showed tolerance for a wide range of nucleophiles and functionalities.

**Scheme 1:**
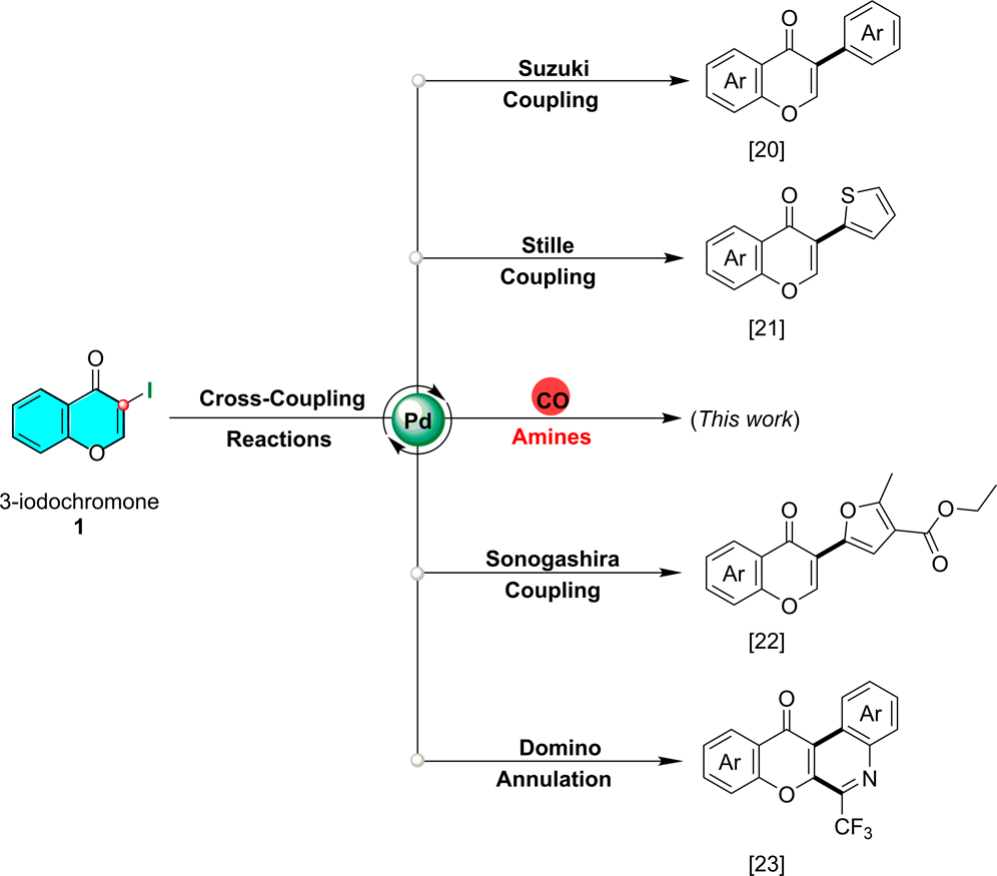
Previously reported Different Cross-Coupling Reactions Involving 3-Iodochromone As Partner

Peculiarly, only conventional amidations have been documented for the synthesis of chromone-3-carboxamide derivatives involving classic carboxylic acids or *in situ* generated acyl chlorides and appropriate amines. Such multistep protocols, which require harsh conditions and coupling reagents (POCl_3_, (phosphorus(V)oxychloride), DCC, (*N*,*N*′-dicyclohexylcarbodiimide), PyBOP, (benzotriazol-1-yloxy)tripyrrolidinophosphonium hexafluorophosphate), suffer from laborious purifications because of the presence of byproducts along with the intermediates.^19^ Notably, 3-iodochromone is an easily accessible and commercialized synthon. It is considered as the most reactive representative in the class of halogenated chromones and seems to be a tolerated partner for different varieties of palladium-mediated C–C couplings, such as Suzuki^20^ and Stille^21^ reactions, leading to isoflavones and 3-thiophenchromone (Scheme 1), Sonogashira C–C coupling and chromone domino-annulation under cooperative palladium/norbornene catalysis, recently published for preparing 3-furanochromones^22^ and chromone-fused heterocyclic compounds,^23^ respectively. Contrastingly, the use of 3-iodochromone in carbonylative cross-couplings has no literature precedent.

Based on our long-standing interest in carbonylation reactions,^24^ and on the screening of different readily available iodoheteroaromatic models, we envisioned the feasibility of aminocarbonylation reaction of this ideal substrate, in the presence of primary and secondary amines, as a new challenging task to build a library of structurally enriched chromone-3-carboxamides. We report herein the results of our investigations on the behavior of 3-iodochromone under palladium-catalyzed aminocarbonylation conditions.

## Results and Discussion

**Scheme 2:**
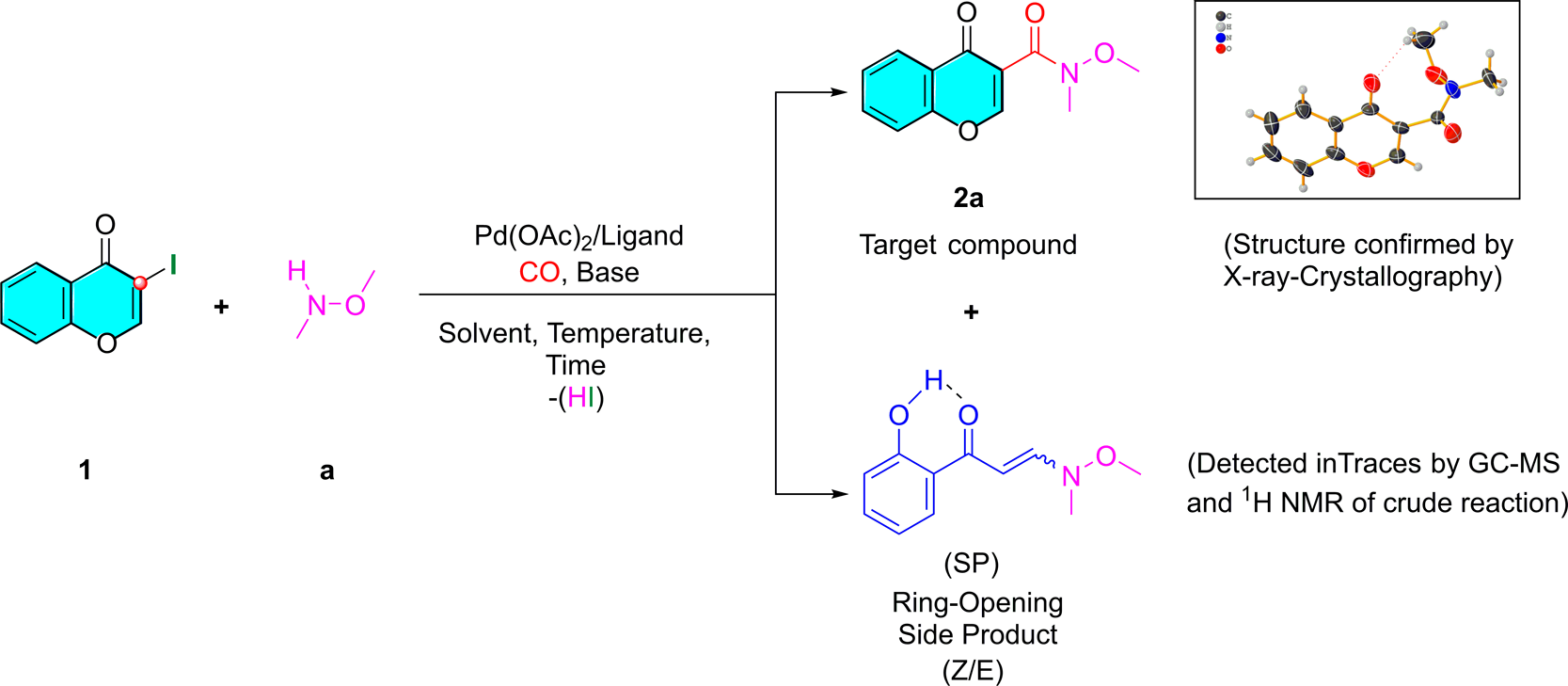
Palladium-Catalyzed Aminocarbonylation of 3-Iodochromone (**1**) with *N*,*O*-Dimethylhydroxylamine (**a**)

First, the focus of our early studies was to perform palladium-catalyzed aminocarbonylation of 3-iodochromone (**1**). As far as we know, this synthetic strategy is adopted to produce chromone-3-carboxamides. For this, *N,O*-dimethylhydroxylamine (**a**) was selected as an amine nucleophile and the reaction was carried out in the presence of a Pd(OAc)_2_/PPh_3_ catalyst under atmospheric carbon monoxide pressure at 50 °C by using Et_3_N (Scheme 2). The reaction was monitored by GC, and the results are given in Table 1.

**Table 1:** Optimization Study of Aminocarbonylation of 3-Iodochromone (**1**) with *N,O*-Dimethylhydroxylamine^a^

entry	base	ligand	solvent	temp (°C)	time (h)	conv.^b^^)^	yield^c^^)^
1	Et_3_N	PPh_3_	DMF	50	48	100	58
2^d^^)^	Et_3_N	PPh_3_	DMF	50	48	100	70
3	Et_3_N	PCy_3_	DMF	50	24	100	62
4	Et_3_N	dppp	DMF	50	24	0	–
5	Et_3_N	dppf	DMF	50	24	100	60
**6**	**Et_3_N**	**XantPhos**	**DMF**	**50**	**6**	**100**	**80**
7	Et_3_N	XantPhos	DMF	100	2	100	68
8	Et_3_N	XantPhos	toluene	50	24	78	–
9	Et_3_N	XantPhos	toluene	100	6	100	75
10	Et_3_N	XantPhos	dioxane	50	6	100	36
11	Et_3_N	XantPhos	dioxane	100	6	100	62
12	Et_3_N	XantPhos	ACN	50	6	100	70
13	Et_3_N	XantPhos	ACN	80	6	100	77
14	Et_3_N	XantPhos	THF	50	6	100	45
15	Cs_2_CO_3_	XantPhos	DMF	50	6	100	54

a Standard reaction conditions: 0.5 mmol of 3-iodochromone (**1**), 0.55 mmol of *N,O*-dimethylhydroxylamine hydrochloride (**a**), 0.025 mmol of Pd(OAc)_**2**_, 0.05 mmol of monodentate (PPh_3_, PCy_3_), or 0.025 mmol of bidentate (XantPhos, dppp, dppf) ligands, 0.5 mL of Et_3_N, or 0.75 mmol of Cs_2_CO_3_, 10 mL of solvents: DMF, toluene, dioxane, THF, or ACN (acetonitrile) at the mentioned temperature under atmospheric pressure of carbon monoxide.

b Determined by GC-MS and ^1^H NMR measurements of the crude reaction mixture.

c Isolated yield; (−) = not isolated.

d Reaction performed under 40 bar of carbon monoxide.

Expectedly, the use of Pd(OAc)_2_/2PPh_3_ catalyst^25^ was able to provide selectively the desired **2a** carboxamide, as main product, under atmospheric carbon monoxide pressure. Total conversion of **1** was achieved in 48 h (Table 1, entry 1).

Aiming to establish unequivocally the skeleton of carbonylated derivative **2a**, crystals suitable for X-ray analysis were grown from the purified compound and subjected to single crystal X-ray diffractometry. The refined structure, undoubtedly, was supported by the spectroscopic data (Scheme 2). The supplementary crystallographic data for this compound is deposited at Cambridge Crystallographic Data Centre under CCDC 2269045 number. (A more detailed crystallographic study can be found in the Supporting Information.)

The aminocarbonylation reaction, performed under the above-mentioned experimental conditions, showed high chemoselectivity toward chromone-3-carboxamide **2a**, isolated in 58%. It has to be noted, that the corresponding ring-opening product was observed by ^1^H NMR and GC-MS analysis of the crude reaction mixture, considering the tendency of 3-iodochromone (**1**) to undergo, in the presence of *N*-nucleophiles, an aza-Michael addition/ring-opening/deiodination process as a side reaction (Scheme 2).^26^

In the next step, detailed optimization study of our model reaction was performed. The effect of carbon monoxide pressure, the type of the ligand, and the influence of solvent, temperature, and base, on palladium-catalyzed carbonylative process were studied (Table 1).

The yield of the desired product **2a** was remarkably increased to 70% when applying high pressure carbon monoxide (40 bar) keeping the same experimental conditions (Table 1, entries 1 and 2). The long-time reaction (48 h), performed under 40 bar CO, was highly selective and no chromone-3-glyoxylamide was detected by ^1^H NMR measurement of the crude. Furthermore, considering the importance of the ligand in aminocarbonylation reaction, different phosphines were screened, looking for more efficient catalyst systems under atmospheric (1 bar) conditions (Table 1, entries 3–6).

By the use of more basic monodentate PCy_3_ or the bidentate phosphine dppf, the yields slightly decreased to 62% and 60% (Table 1, entries 3 and 5), respectively. No reaction was observed when dppp was introduced under the same conditions (Table 1, entry 4).

Promisingly, the use of Pd(OAc)_2_/XantPhos in 1:1 ratio, selectively, led to the target carboxamide **2a** within 6 h and important increase of yield (80%) was observed, compared to the reaction performed with triphenylphosphine (Table 1, entries 1 and 6).

With XantPhos as the ligand of choice, various solvents were explored and the influence of temperature was also investigated, using Et_3_N as a base. When the reaction was carried out in DMF at 100 °C, no increase in yield was shown (compare entries 6 and 7), while with the use of a nonpolar solvent such as THF at 50 °C, total conversion was accomplished after 6 h and the desired compound was given in only 45% isolated yield (entry 14). Conversely, only 78% of starting material **1** was consumed after 24 h reaction time in toluene at 50 °C (entry 8), and the reaction was shifted mainly toward the ring-opened side product (SP) formation as proven also by GC-MS measurement. Instead, complete conversion was detected (Table 1, entry 9) in toluene at 100 °C with a shortening of reaction time (6 h), and crucial improvement of isolated yield (75% of **2a**) was observed clearly. Similarly, the aminocarbonylation reaction, performed in acetonitrile and dioxane, seemed to be temperature-dependent as the best yields (62% and 77%, respectively) could be obtained, within 6 h, when higher temperatures (80 and 100 °C) were applied (Table 1, entries 10–11 and 12–13). A significant decrease in the yield (54%) was detected, when triethylamine (Et_3_N) was replaced by Cs_2_CO_3_ as an inorganic base (Table 1, compare entries 6 and 15).

To sum up, the above-discussed optimization experiments revealed that the use of XantPhos in DMF at 50 °C under atmospheric carbon monoxide pressure were the optimal conditions for the aminocarbonylation reaction of substrate **1**, providing successfully the final chromone-3-carboxamide (**2a**) in good yield.

In order to examine the scope of this reaction with various *N*-nucleophiles, we decided to check a set of secondary amines, applying the protocol model under optimized conditions. The results were promising as several amines (**a**–**h**) were able to produce the corresponding chromone-3-carboxamides (**2a**–**h**) in moderate to excellent yields. As can be seen in Table 2, an aliphatic amine such as diethylamine (**b**) successfully afforded the expected carbonylative compound **2b** in moderate yield (52%), within 2 h (Table 2, entry 2). With the use of l-proline methyl ester, the target carboxamide (**2c**) was provided in 61% (entry 3). While the *N*-methylbenzylamine (**d**) produced the corresponding **2d** product (Table 2, entry 4) in 68% of isolated yield, in the presence of bulky nortropinone (**e**), the **2e** chromon-3-carboxamide was synthesized only in 47% of yield (Table 2, entry 5). Moreover, the use of 4-hydroxy-*N*-methylaniline (**f)**, as an aromatic secondary amine, led to the expected amide (**2f**) in a moderate 45% of yield (entry 6). Furthermore, both picolylamine derivatives (**g**, **h**) were compatible and provided compounds **2g** and **2h** in good yields (Table 2, entries 7 and 8).

**Table 2:**
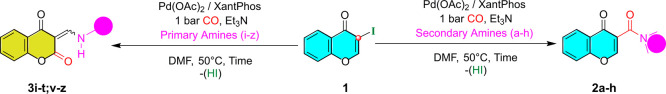
Scope of Primary and Secondary Amines^a^

				carboxamides (**2**) and chroman-2,4-diones (**3**)	
entry		amines	time (h)	ratio^b^ (**2**/**3**)	yield^c^ (**2**/**3**)
1	secondary amines	*N*,*O*-dimethylhydroxylamine (**a**)	6	(100/0)	(80/−)
2		diethylamine (**b**)	2	(100/0)	(52/−)
3		l-proline methyl ester (**c**)	6	(100/0)	(61/−)
4		*N*-methylbenzylamine (**d**)	24	(100/0)	(68/−)
5		nortropinone (**e**)	48	(100/0)	(47/−)
6		4-hydroxy-*N*-methylaniline (**f**)	6	(100/0)	(45/−)
7		(4-ethylaminomethyl)pyridine (**g**)	2	(100/0)	(72/−)
8		di-(2-picolyl)amine (**h**)	6	(100/0)	(51/−)
9	primary amines	*O*-methylhydroxylamine (**i**)	6	(10/90)	(10/70)
10		*tert*-butylamine (**j**)	1	(0/100)	(−/40)
11		benzylamine (**k)**	2	(0/100)	(−/50)
12		phenethylamine (**l**)	4	(0/100)	(−/55)
13		cyclopentylamine (**m**)	4	(0/100)	(−/54)
14		glycine methyl ester (**n**)	4	(0/100)	(−/57)
15		l-alanine methyl ester (**o**)	2	(06/94)	(3/41)
16		l-valine methyl ester (**p**)	6	(03/97)	(−/60)
17		(*S*)-(+)-2-phenylglycine methyl ester (**q**)	4	(0/100)	(−/90)
18		aniline (**r**)	2	(03/98)	(4/80)
19		2-aminopyridine (**s**)	2	(04/96)	(−/82)
20		3-aminopyridine (**t**)	2	(03/97)	(−/90)
21		4-aminopyridine (**u**)	2	(99/01)	(85/−)
22		*ortho*-phenylenediamine (**v**)	2	(0/100)	(−/89)
23		*ortho*-aminophenol (**w**)	2	(0/100)	(−/92)
24		piperonylamine (**x**)	4	(0/100)	(−/60)
25		3,4-dihydroxybenzylamine (**y**)	6	(0/100)	(−/40)
26		diethyl-α-aminobenzylphosphonate (**z**)	2	(0/100)	(−/44)
27		D/L-noradrenaline (**a**′)	6	(0/100)	(−/45)

a Experimental protocol: 0.5 mmol of 3-iodochromone (**1**), primary and secondary amine nucleophile: 0.55 mmol of solid amines (or 1.5 mmol of *tert-*butylamine or 0.75 mmol of other liquid amines), 0.025 mmol of Pd(OAc)_2_, 0.025 mmol of XantPhos, 0.5 mL of Et_3_N, 10 mL of dry DMF, at 50 °C, under 1 bar of carbon monoxide.

b Ratio determined based on GC and GC-MS measurements, supported by crude reaction ^1^H NMR analysis.

c Isolated yield; (−): not isolated.

**Scheme 3:**
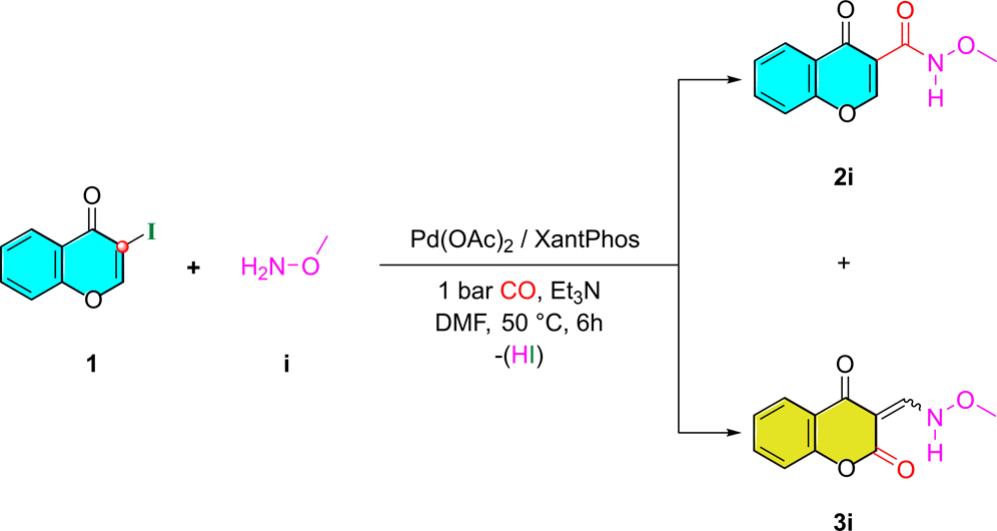
Aminocarbonylation Reaction of 3-Iodochromone **1** with *O*-Methylhydroxylamine (**i**) under Optimized Conditions

In the next part of our study, we turned our attention to the use of primary amines in order to check the applicability of the optimized aminocarbonylation protocol and extend the chromone-3-carboxamides series. Initially, the investigation was begun with *O*-methylhydroxylamine (**i**) (Scheme 3, Table 2, entry 9) providing, unexpectedly, a mixture of two types of carbonylated compounds in a ratio of 10:90 (**2i**:**3i**). The GC-MS analysis of the obtained mixture and the detailed NMR comparison of the isolated compounds (**2i**:**3i**), based on the literature,^27^ revealed the presence of the corresponding chromone-3-carboxamide (**2i**) as well as the unexpected 3-functionalized chromane-2,4-dione (**3i**).

On the basis of the above-mentioned results in the reaction of substrate **1** with **i**, it has to be concluded that under palladium-catalyzed aminocarbonylation conditions, 3-iodochromone (**1**) could undergo an uncommon carbonylative transformation in the presence of primary amines. Moreover, the chromane-2,4-dione derivative (**3i**) was selectively provided instead of the chromone-3-carboxamide counterpart (**2i**). It could be postulated that the formation of the **3** isomer is directly connected to the pronounced tendency of chromone framework to undergo an ANRORC (an acronym standing for addition of the nucleophile, ring opening, and ring closure) rearrangement with various external and internal nucleophiles.^28^ Presumably, the unexpected chroman-2,4-dione (**3i**) is the ANRORC product resulting from a late-stage carbonyl insertion, concomitantly to the favored ring-closure aryloxycarbonylation step during the ANRORC process. This reasonable pathway seems to be intriguing and provides an explanation for this unusual carbonylative transformation under palladium-catalyzed aminocarbonylation.

To examine this behavior of our starting iodo-heteroarene compound (**1**), a bunch of primary amines (**j**–**z**) was selected and tested in the aminocarbonylation of **1** under optimized conditions (Table 2 and Table 3).

**Table 3:**
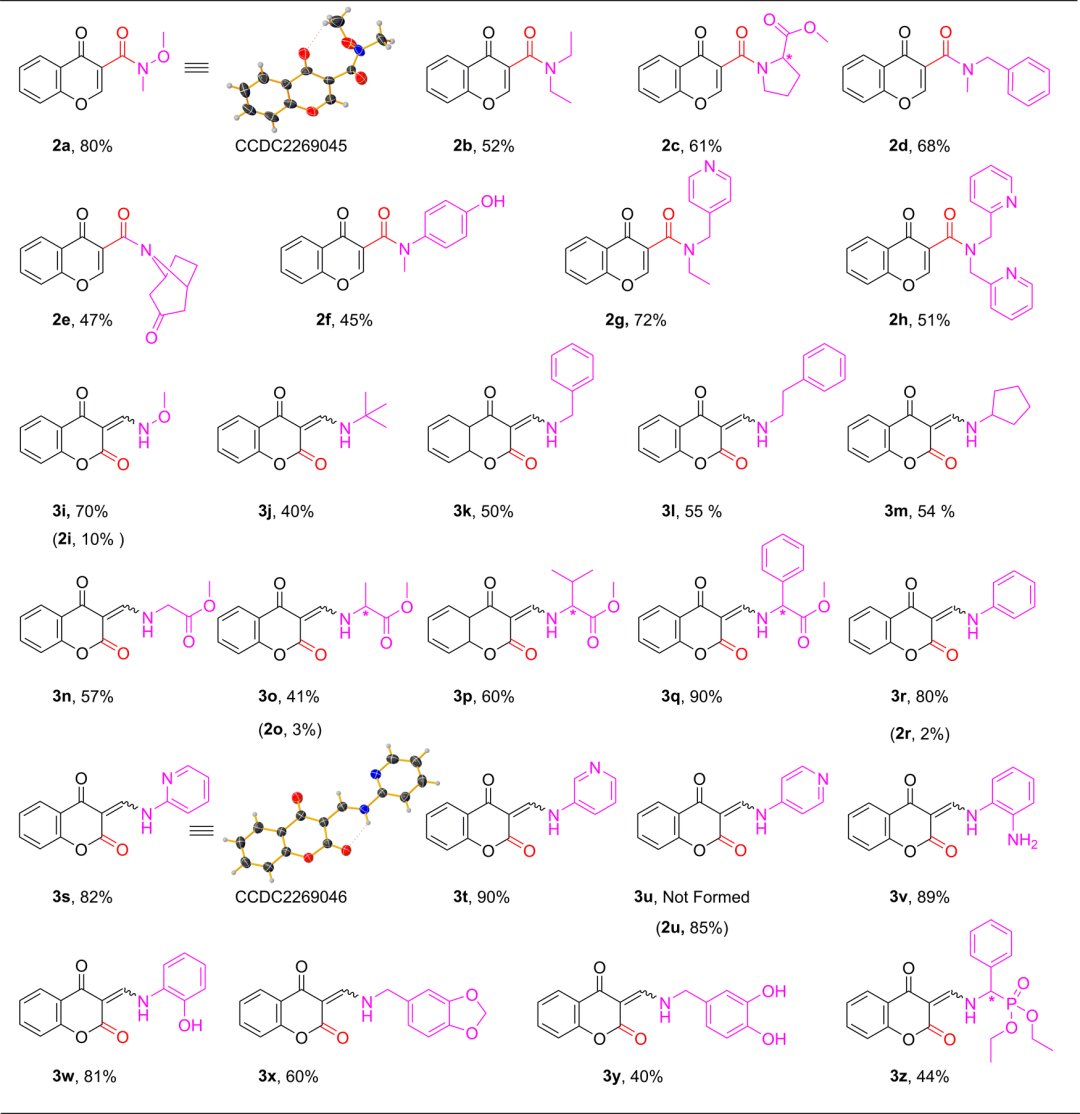
Summary of Prepared Chromone-3-carboxamides (**2a**–**h**) and Chroman-2,4-diones (**3i–z)**

The reaction with *tert*-butylamine (**j**) gave exclusively the corresponding 3-*tert*-butylaminomethylidene-chroman-2,4-dione (**3j**) in 40% of isolated yield. The NMR spectra of **3j** exhibit a double set of signals that points out the existence of a mixture of **Z/E** isomers, with one form predominating, as previously reported in the literature for this compound family.^29^ The double signals also occur for almost all prepared chroman-2,4-diones (except for **3i**), isolated as main products (see spectra in the Supporting Information), which further confirm the high selectivity of the reaction toward the ANRORC type compound family **3**.

The screening of aliphatic primary amines (**k**–**m**) led selectively to the corresponding chroman-2,4-diones (**3k**–**m**) in moderate to good yields (50–55%) (Table 2, entries 11–13).

The selected α-amino acid methyl esters (**n**–**q**) and diethyl α-aminobenzylphosphonate (**z**) were well-tolerated for producing desired products (**3n**–**3q**) in 44% to 90% of yields (Table 2, entries 14–17, 26). When the reaction was performed with alanine methyl ester (**o**), the corresponding carboxamide form (**2o**) was also isolated in traces (Table 2, entry 15). On the other hand, aromatic amines such as aniline (**r**), 2- and 3-aminopyridine (**s**, **t**) showed perfect compatibility to the reaction furnishing the expected chroman-2,4-diones (**3r**, **3t**) in excellent yields (Table 2, entries 18–20). Exceptionally, 4-aminopyridine (**u**) failed to give the corresponding chroman-2,4-dione (**3u**). Instead, only the chromone-3-carboxamide (**2u**) was isolated in 85% yield (Table 2, entry 21). The use of *ortho*-phenylenediamine (**v**) and *ortho*-aminophenol (**w**) gave excellent selectivity providing 89% to 92% isolated yields, respectively. It is worth mentioning that no side product formation was observed as the free amino- and hydroxyl groups remained untouched (Table 2, entries 22–23). Finally, coumarine-based piperonylamine (**3x**) and 3,4-dihydroxybenzylamine-containing hybrid (**3y**) were isolated in 60% and 40% yields, respectively (Table 2, entries 24–25). We examined the applicability of the present protocol to access a coumarin-based catecholamine hybrid of biological importance.^30^ In this way, the chroman-2,4-dione-noradrenaline (**3a**′) derivative was successfully prepared in 45% of yields (Table 2, entry 27), which exhibits a strong yellow-orange fluorescence under 365 nm UV irradiation, that makes **3a**′ a promising candidate to be used as a potential visual prodrug marker.

The molecular structure of chroman-2,4-dione **3s** was unambiguously elucidated by single-crystal X-ray diffraction analysis (Deposition Number 2269046). Only *Z*-isomer has been observed in solid state because of the fixed conformation of enaminone moiety, probably, because of requirements of the crystal lattice. Further details are discussed in the crystallographic study part found in the Supporting Information.

## Proposed Catalytic Cycles

Since the chromone-3-carboxamides (**2a**–**h**) and chroman-2,4-diones (**3i**–**z**) were formed from the same starting material under the same experimental conditions, the unprecedented behavior of 3-iodochromone (**1**) to dissimilarly undergo CO-insertion processes in the presence of primary and secondary amines seems to be mechanistically interesting.

From the gathered data, the identification of chromone-3-carboxamide form, as main product in the case of all subjected secondary amines, leads to the conclusion that palladium-catalyzed aminocarbonylation of 3-iodochromone is successfully favored in a selective manner. The target carboxamides (**2a**–**2h**) were formed according to the well-known catalytic cycle of the palladium-catalyzed aminocarbonylation.^31^

On the other hand, in an unexpected way, the aminocarbonylation reaction of 3-iodochromone (**1**) carried out in the presence of primary amines selectively provided the chroman-2,4-diones. The chromone-3-carboxamide counterpart was detected by GC-MS, only in a few cases of primary amines, and corresponding derivatives (**2i**, **2o**, and **2r**) were isolated in vestigial amounts (except in case of 4-aminopyridine, where the main product was the chromone-3-carboxamide form (**2u**)).

As a first assumption, the higher chemoselectivity observed toward chroman-2,4-diones could be explained by the stability of the six-membered lactone formed. Moreover, the coumarin-enamine form (chroman-2,4-dione) is further stabilized by resonance-assisted intramolecular hydrogen bonds (RAHB) existing across the planar β-enaminone fragments in (*Z*) and (*E*) isomers that gives rise to a continuous π-electron delocalization across the [···O=C—C=C—NH···] pseudo ring system and consequently establishes an extended planarity which means more stability for the structure (see Scheme S3).^32^ These interesting structural features have been proven by NMR and XRD analyses in solution as well as in solid state (see detailed crystallographic data in the Supporting Information). We could presume, thereby, that the RAHB pattern is the driving force for this elegant transformation favoring chroman-2,4-diones formation when primary amines were used.^33^

**Scheme 4:**
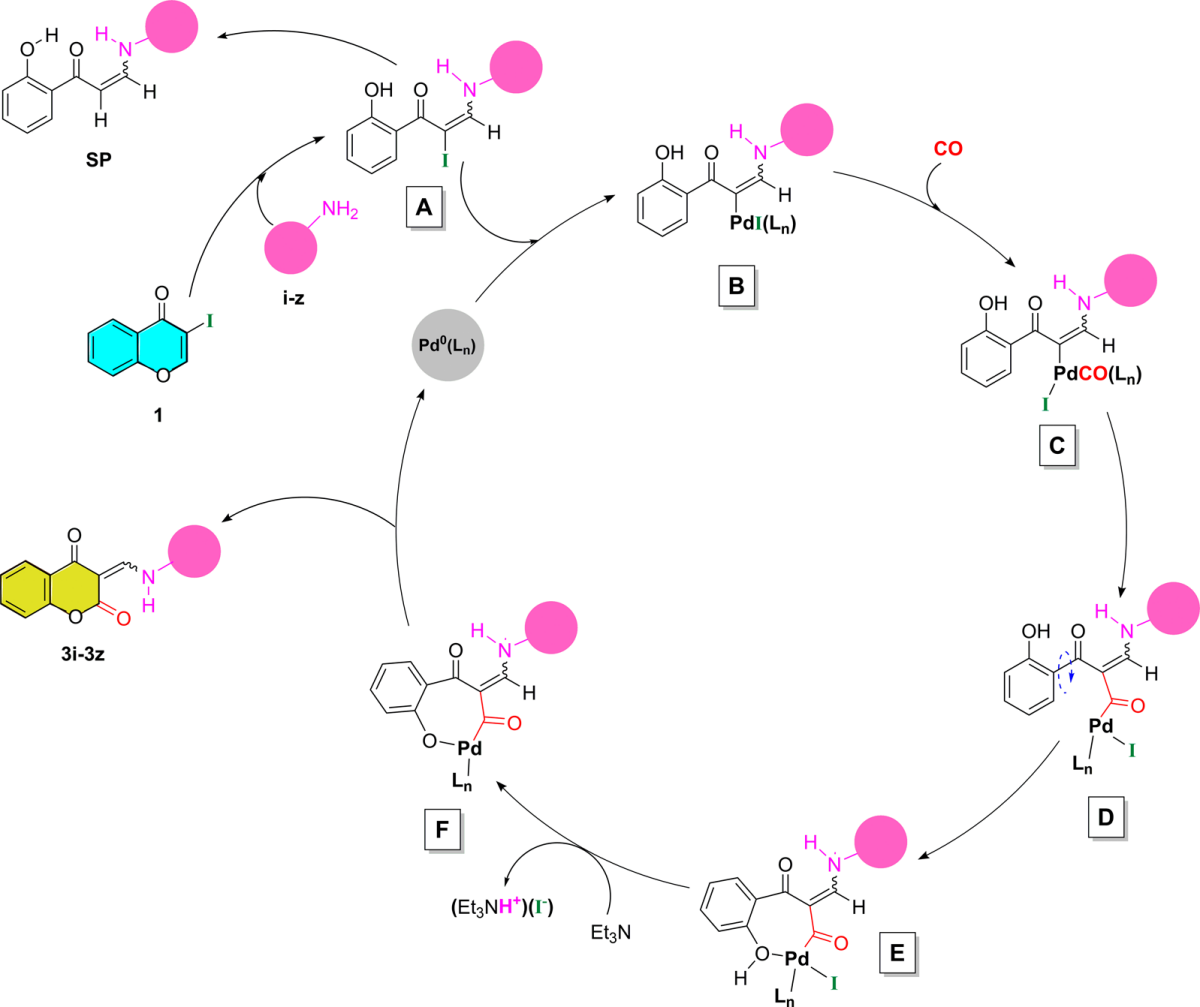
Plausible Mechanism for the Chroman-2,4-diones Formation

Mechanistically, as we mentioned above, the chromone ring is able to promote ANRORC rearrangement. These steps are, presumably, pivotal for a late-stage carbonyl insertion process, giving access to the final chromane-2,4-dione. Consequently, the 3-iodochromone (**1**) could undergo an intramolecular aryloxycarbonylation-cyclization ring-closure step, involving the *in situ* generated phenolic hydroxyl group. The plausible mechanism proposed for the chroman-2,4-dione formation, under palladium-catalyzed aminocarbonylation conditions, could be described as a hybrid version of the ANRORC rearrangement (Scheme 4). Hence, the reaction was carried out through an aza-Michael addition of amine at the C-2 position of the chromone framework with a subsequent pyrone ring cleavage to produce probably the iodinated key intermediate (**A**) *in situ*. Next, an oxidative addition on the carbon–iodine bond occurred, enabling the formation of species **B**. The terminal carbonyl species (**C**), resulting from the carbonyl-coordination to intermediate **B**, gave acylpalladium(II) species (**D**) *via* migratory insertion of the carbonyl ligand. Then, the coordination of the free phenolic hydroxyl group led to the aryloxypalladated cycle (**F**) upon a base-promoted proton’s extraction. Finally, the target product is delivered through a reductive elimination step with a concomitant cyclization process.

To shed light on the underlying mechanism, simple control experiments were carried out with substrate **1** in the presence of a primary amine in absence of carbon monoxide and/or catalyst. The results provided evidence for the *in situ* formation of the unstable intermediate (**A**), which could undergo a dehydroiodination process leading to the corresponding ring-opening side product (**SP**). (Further details are included in the Supporting Information).

## Conclusion

In conclusion, various practically important chromone-3-carboxamides and chroman-2,4-diones were prepared, starting from 3-iodochromone, under palladium-catalyzed aminocarbonylation conditions. The present study revealed that using Pd(OAc)_2_/XantPhos as catalyst and Et_3_N at 50 °C, under atmospheric conditions, the chemoselectivity of the reaction is governed by two different catalytic carbonylative processes. This protocol exclusively provides chromone-3-carboxamides in good yields when secondary amines are used. Instead, the same protocol offers a convenient and high-yield domino carbonylative transformation for selective synthesis of 3-substituted chromone-2,4-diones, tolerating a large variety of primary amines. The reaction conditions are simple and sufficiently mild to be applied as a promising alternative strategy for further functionalization of chromones and as a novel synthetic approach to access the chroman-2,4-dione framework. Additionally, a new plausible ANRORC rearrangement has been proposed for chroman-2,4-diones formation, involving intramolecular aryloxycarbonylation as the last ring-closure step.

## Data Availability

The data underlying this study are available in the published article and its Supporting Information.

## Supporting Information Available

Experimantal processes; XRD study of compounds **2a** and **3s**; characterization and copies of NMR spectra (^1^H, ^13^C) of the prepared derivatives (PDF)
