# Supplementary material for: Synthesis of Chroman-2,4-diones via Ring-Opening/Ring-Closing Reaction Involving Palladium-Catalyzed Intramolecular Aryloxycarbonylation
Source: J Org Chem. 2024 Jan 9;89(2):1175–83. doi: 10.1021/acs.joc.3c02337 (PMC10804402; doi:10.1021/acs.joc.3c02337)
Supplement: Supplementary file 1 — jo3c02337_si_001.pdf [file jo3c02337_si_001.pdf]

Supporting Information

Synthesis of Chroman-2,4-diones *via* Ring-Opening/Ring-Closing Reaction Involving Palladium-Catalyzed Intramolecular Aryloxycarbonylation

Sami Chniti,[a] Péter Pongrácz,[a] László Kollár,[a,b,c] Attila Bényei,[d] Ágnes Dörnyei,[e] Attila Takács*,[b,c]

[a] Department of General and Inorganic Chemistry, Faculty of Sciences, University of Pécs, Ifjúság u. 6., Pécs, H-7624, Hungary.

[b] János Szentágothai Research Centre, University of Pécs, Ifjúság u. 20., Pécs, H-7624, Hungary.

[c] HUN-REN-PTE Research Group for Selective Chemical Syntheses, Ifjúság u. 6., Pécs, H-7624, Hungary.

[d] Department of Physical Chemistry, University of Debrecen, Egyetem tér 1., Debrecen, H-4032, Hungary

[e] Department of Analytical and Environmental Chemistry, Faculty of Sciences, University of Pécs, Ifjúság u. 6., Pécs, H-7624, Hungary.

*e-mail Attila Takács: takacsattila@gamma.ttk.pte.hu

Table of Contents

1. Experimental Section S1.
2. Preliminary Mechanistic Studies of Chroman-2,4-dione's Formation. S3.
3. X-ray Crystallographic Study S5.
4. Characterization of the synthesized compounds S14.
5. Copies of the ^{1}H and ^{13}C{^{1}H} NMR spectra of the synthesized compounds S28.

1. Experimental Section

The $Pd(OAc)_2$, the ligands (PPh_3 (triphenylphosphine), PCy_3 (tricyclohexylphosphine), dppf (1,1'-bis(diphenylphosphino)ferrocene), dppp (1,3-bis(diphenylphosphino)propane) and XantPhos (4,5-bis(diphenylphosphino)-9,9-dimethylxanthene)), the bases (Et_3N, Cs_2CO_3), the solvents, and the amine nucleophiles (**a-z**) were purchased from Sigma-Aldrich (St. Louis, MO, USA) and were used without any further purification. Precoated silica gel $60F_{254}$ plates were used for thin layer chromatography (TLC) and were also purchased from Sigma-Aldrich. Column chromatography was performed with 0.063-0.200 mm mesh silica gels. ^1H, ^{13}C and ^{31}P NMR spectra were recorded in $CDCl_3$ or DMSO-d_6 on a Bruker Avance III 500 spectrometer (Bruker BioSpin Corp., Karlsruhe, Germany) at 500, 125 and 202 MHz, respectively. Chemical shifts δ are reported in ppm relative to $CDCl_3$ (7.26 and 77.00 ppm for ^1H and ^{13}C, respectively) or DMSO-d_6 (2.50 and 39.50 ppm for ^1H and ^{13}C, respectively). The FT-IR spectra were taken in KBr pellets using a Nicolet IMPACT 400 spectrometer (Thermo Fisher Scientific, Waltham, MA, USA) applying a DTGS detector in the region of 400-4000 cm^{-1}, resolution was 4 cm^{-1}.
The GC measurements have been performed with a Shimadzu GC-2030 gas-chromatograph (Shimadzu, Tokyo, Japan) fitted with a capillary column (DB-1) (injector temp. 250 °C; oven: starting temp. 50 °C (hold-time 1 min), heating rate 15 °C min^{-1}, final temp. 320 °C (hold-time 11 min); detector temp. 280 °C; carrier gas: helium (rate: 1 mL min^{-1})).
Mass spectrometry data were recorded using a GC–MS-2020 system (Shimadzu, Tokyo, Japan) operated in EI mode (70 eV). The data are given for the corresponding compounds as mass unit per charge (m/z) and intensities are given in brackets in the Supplementary file. High-resolution mass spectra were acquired on a 6530 Accurate-Mass Quadrupole Time-of-Flight (Q-TOF) LC/MS system (Agilent Technologies, Singapore) equipped with an Agilent Jet Stream electrospray ionization (ESI) source.
The starting material (**1**) was prepared according to the reported procedure[1]. Compounds **2b**[2], **3k**[3], **3l**[3], **3r**[3], **3s**[3], **3v**[4], **3w**[4] have been described in the literature. These compounds were also purified, characterized and the spectral data were in accordance with the literature data.

Synthesis of 3-iodochromone (1)

The 3-iodochromone **1** was an easily accessible substrate that could be prepared following a modified two-step protocol previously reported, which involves commercially available starting materials. Thus, the first key synthon was prepared *via* methylidenation reaction : a mixture of 2-hydroxyacetophenone (1.22 mL, 10 mmol) was treated with DMF-DMA (*N*,*N*-dimethyformamide dimethyl acetal) (2.65 mL, 20 mmol) and heated at 90 °C overnight in dry

toluene (5 mL), and allowed to cool. The reaction was worked up by removing solvent using vacuum evaporation. The crude reaction was then subjected to column chromatography using a mixture of hexane/ethyl acetate as eluant, providing 3-(dimethylamino)-1-(2-hydroxyphenyl)propenone in good yield (76%).

Then, the isolated enaminone was subjected to an I_2-mediated oxydative cyclisation procedure to provide the final 1,4-benzopyrone ring **1** with an iodine atom at C3-position. For this a mixture of 3- (dimethylamino)-1-(2-hydroxyphenyl) propenone (5 mmol) in $CHCl_3$ (75 ml) and iodine (10 mmol, 2 mol equiv), followed by stirring the mixture at 25°C for 18 h. The formation of products was confirmed by thin-layer chromatography (TLC) in ethyl acetate:hexane (3:7) solvent system. The solution was washed with saturated $Na_2S_2O_3$ (15 ml), and the organic phase was collected. The solvent was removed using rotary evaporator. The synthesized chromone was purified by column chromatography to afford the pure chromone **1** in 86% yield.

General method for the synthesis of chromone-3-carboxamides (2) and chroman-2,4-diones (3) under atmospheric conditions.

In a typical experiment, $Pd(OAc)_2$ (5.6 mg, 0.025 mmol), XantPhos (14.4 mg, 0.025 mmol), 3-iodochromone (**1**) substrate (0.5 mmol, 136 mg), amine nucleophiles ((**a**-**z**), amount of the amines are given in Table 1), and triethylamine (0.5 mL), were dissolved in DMF (10 mL) under argon in a 100 mL three-necked flask equipped with reflux condenser connected to a balloon filled with argon. The reaction vessel was flushed with argon. The atmosphere was then changed to carbon monoxide. (Caution: Carbon monoxide atmosphere should only be used with adequate ventilation (hood) using CO sensors as well.) The reaction was conducted for the given reaction time upon stirring at 50-100 °C by using a heat-on block (Table 1) and analyzed by GC and GC-MS. The cooled reaction mixture was then concentrated and evaporated to dryness under reduced pressure. The residue was dissolved in chloroform (20 mL) and washed twice with water (20 mL). The organic phase was dried over Na_2SO_4, filtered, and evaporated under reduced pressure to give a solid material. All compounds were subjected to column chromatography (Silicagel 60 (Sigma), 0.063-0.200 mm), using different eluent mixtures (the exact eluents (content, ratio) are specified in Characterization Part found in the Supplementary File).

General method of a high pressure aminocarbonylation process.

In a typical experiment, $Pd(OAc)_2$ (5.6 mg, 0.025 mmol), PPh_3 (13.1 mg, 0.05 mmol), 3-iodochromone (**1**) substrate (0.5 mmol, 136 mg), *N*,*O*-dimethylhydroxylamine hydrochloride (0.55 mmol, 53.6 mg), and triethylamine (0.5 mL), were dissolved in DMF (10 mL) under argon in a 100 mL stainless steel autoclave. The atmosphere was changed to carbon monoxide and the autoclave was pressurized to the given pressure with carbon monoxide. (Caution: High pressure carbon monoxide should only be used with adequate ventilation (hood) using CO sensors as well.) The reaction was conducted for 48 h reaction time upon stirring at 50 $^{\circ}$C in an oil bath. After the given reaction time the reaction mixture was cooled to room temperature and the autoclave was carefully depressurized in a well-ventilated hood. The product mixture was analyzed by GC and GC-MS.

Gramm-Scale synthesis of chroman-2,4-dione 3w:

To demonstrate the potential utility of our protocol, the representative chroman-2,4-dione **3w** was scaled up to gramm-scale and reasonable yield (75 %) was obtained (Scheme S1). The above-mentioned experimental procedure was followed to produce chroman-2,4-diones **3w**, starting from 1.36 g of 3-iodochromone **1** using only 2.5 mol % of catalyst.

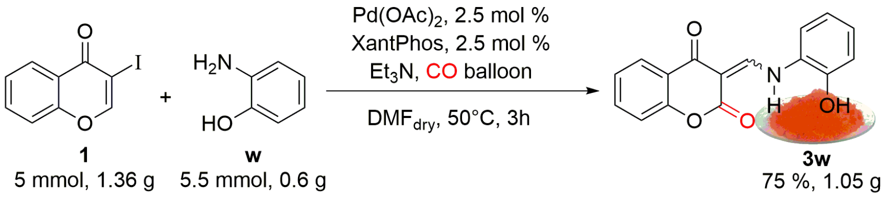

Scheme S1. Larger scale reaction.

2. Preliminary Mechanistic Studies of Chroman-2,4-dione's Formation.

To gain more insights into this unusual carbonylation mechanism, and to acquire more evidences to explain the selective formation of chroman-2,4-diones **3**, two simple control experiments were carried out (Scheme S2) using benzylamine (**w**) as primary amine model. According to ^{1}H NMR measurements of crudes of carbon monoxide-free reaction **(a)** and carbon monoxide/catalyst-free reaction **(b)**, the iodinated ring-opening product (**A**) resulting from the nucleophilic attack of benzylamine at the C-2 position of chromone ring was not detected. Instead, only corresponding enaminone (**SP**) was generated *in situ* (Figure S1), probably arising from the hydrodeiodination process (a reductive cleavage of the carbon-iodine bond) of the unstable intermediate (**A**). The latter enaminone (**SP**) was isolated from crudes by

column chromatography on silica gel and characterized by ^{1}H and ^{13}C NMR as (**Z**) isomer as compared to litterature data (Figure S1, see also NMR spectra section: Figure S65/S66).[5]

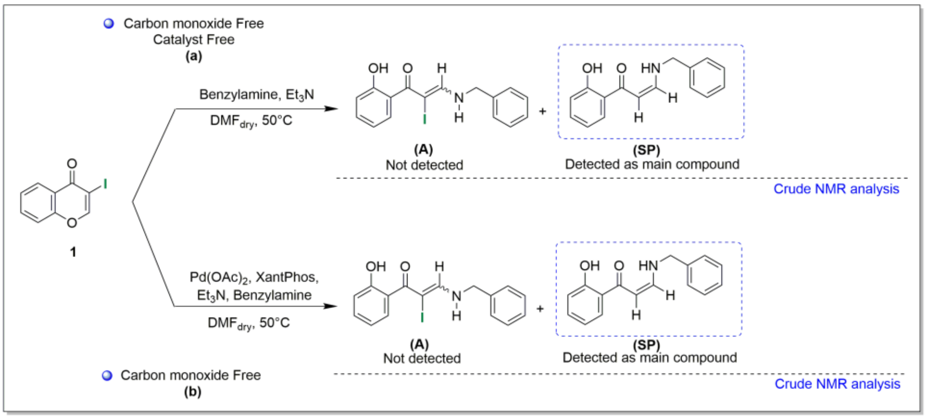

Scheme S2. Control Experiments.

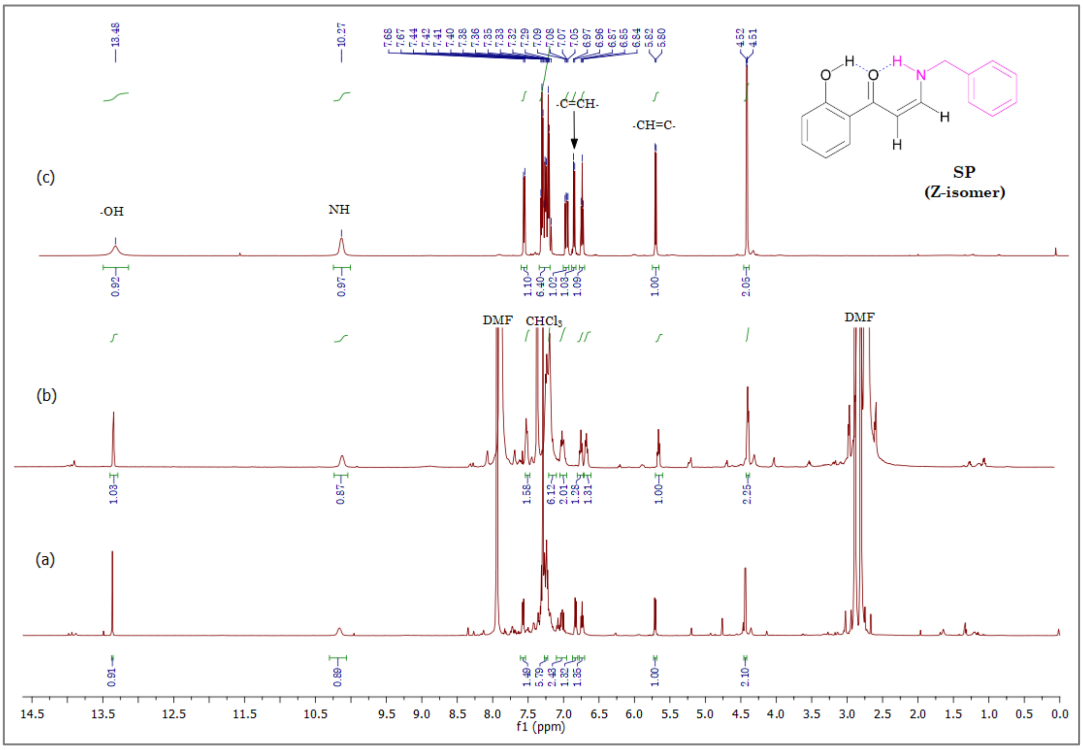

Figure S1. Comparison of ^{1}H NMR (500 MHz, $CDCl_3$) spectra of: carbon monoxide/catalyst free crude reaction **(a)**, carbon monoxide free crude reaction **(b)**, isolated ring-opening product **SP (c)**.

These results are in accordance with previously reported data dealing with the tendency of 2-unsubstituted-3-iodochromone to undergo a free-metal aza-Michael addition/ring-opening/deiodination process in the presence of *N*-nucleophiles. Furthermore, the control experiments herein could support the proposed mechanism for chroman-2,4-diones **3** where a ring opening step takes place before the starting of catalytic cycle, producing intermediate A which should be involved later in the catalytic carbonylation cycle.[6]

3. X-ray Crystallographic Study

The unambiguous molecular structures of **2a** and **3s** have been established by X-ray diffraction analysis. X-ray quality crystals of both compounds were grown from the slow evaporation of concentrated solutions of chloroform or deuterated chloroform, upon standing storing at 4–5 °C. A properly chosen suitable single crystal was, then, fixed under a microscope onto a Mitegen loop using high-density oil. Diffraction Intensity data was collected at ambient temperature (294 K) on a Bruker-D8 Venture diffractometer (Bruker AXS GmbH, Karlsruhe, Germany) equipped with INCOATEC IμS 3.0 (Incoatec GmbH, Geesthacht, Germany) dual (Cu and Mo) sealed tube micro sources and a Photon II Charge-Integrating Pixel Array detector (Bruker AXS GmbH, Karlsruhe, Germany) using Mo Kα (λ = 0.71073 Å) radiation.

High-multiplicity data collection and integration were performed using APEX3 (version 2017.3-0, Bruker AXS Inc., 2017, Madison, WI, USA) software. Data reduction and multiscan absorption correction were performed using SAINT (version 8.38A, Bruker AXS Inc., 2017, Madison, WI, USA). The structure was solved using direct methods and refined on F^2 using the SHELXL program[7] incorporated into the APEX3 suite. Refinement was performed anisotropically for all non-hydrogen atoms. Hydrogen atoms were placed in idealized positions on parent atoms in the final refinement.

The CIF file was manually merged using publCIF software,[8] while graphics were designed using the ***Olex2*** program.[9] The results for the X-ray diffraction structure determinations were in accordance with the Checkcif functionality of PLATON software (Utrecht University, Utrecht, the Netherlands),[10] and structural parameters, such as bond length and angle data, are in the expected range. Details of the crystal parameters, data collection, and structure refinement are given in Table S1. Selected geometric parameters for these structures are given in Table S2. The supplementary crystallographic data for each compound can be obtained free of charge from the Cambridge Crystallographic Data Centre *via* http://www.ccdc.cam.ac.uk/data_request/cif using reference deposition numbers: 2269045 for **2a**, and 2269046 for **3s**.

Table S1. Crystallographic parameters and refinement details for (**2a**), and (**3s**).

**Compounds**	**2a**	**3s**
**Chemical formula**	$C_{12}H_{11}NO_4$	$C_{15}H_{10}N_2O_3$
**M_r**	233.22	266.25
**Crystal system**	Triclinic	Monoclinic
**Space group**	P-1	$P2_1/n$
**Temperature (K)**	300	293
**a, b, c (Å)**	6.7870 (7), 7.4840 (6), 11.2710 (11)	4.9887 (4), 8.8428 (6), 27.0506 (18)
**α, β, γ (°)**	90.160 (5), 93.006 (5), 106.962 (5)	90, 91.110 (3), 90
**V ($Å^3$)**	546.74 (9)	1193.09 (15)
**Z**	2	4
**D (g cm^{-3})**	1.417	1.482
**Radiation type**	Mo Kα	
**μ (mm^{-1})**	0.11	
**Crystal size(mm)**	0.49 × 0.06 × 0.05	0.34 × 0.12 × 0.07
	**Data collection**	
**Diffractometer**	Bruker D8 VENTURE	
**Absorption correction**	Multi-scan SADABS2016/2 - Bruker AXS area detector scaling and absorption correction	
**T_{min}, T_{max}**	0.48, 0.99	0.54, 0.74
**No. of measured, independent and observed [I > 2σ(I)] reflections**	11949, 2075, 1521	16835, 2254, 1682
**R_{int}**	0.132	0.076
**$(\sin θ/λ)_{max}$ ($Å^{-1}$)**	0.611	0.609
**Refinement**		
**R[F^2 > 2s(F^2)], wR(F^2), S**	0.129, 0.329, 1.18	0.052, 0.130, 1.05
**No. of reflections**	2075	2254
**No. of parameters**	157	185
**H-atom treatment**	H atoms are treated by a mixture of independent and constrained refinement	
**$Δ>_{max}$, $Δ>_{min}$ (e $Å^{-3}$)**	0.58, -0.45	0.18, -0.15

As illustrated in Figure S2 title compound *N-Methoxy-N-methyl-4-oxo-4H-chromene-3-carboxamide* **2a** crystallized in the triclinic space group P-1. The crystal structure of **2a** is solved and refined with two symmetry-independent molecules.

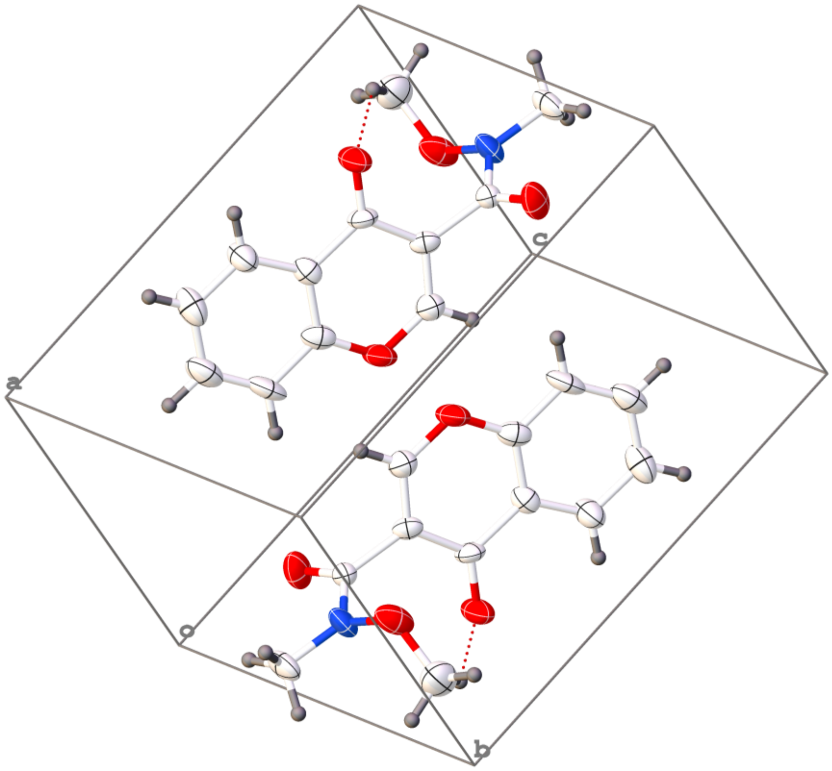

Figure S2. View of asymmetric unit of **2a**, [View normal to (110)], showing thermal displacement ellipsoids, drawn at the 50% probability level. Graphics were designed using ***Olex2*** program.

An inspection of the crystal lattice content showed that all molecules exhibit the unique *anti* conformation and no rotation around the amide bond has been detected in solid state as in case of solution based on NMR analysis (Figure S2). This constrained conformation with a *trans*-amide orientation encloses a network of inter- and intramolecular hydrogen bonds that stabilizes the final crystal packing of **2a** (Figure S3, Top).

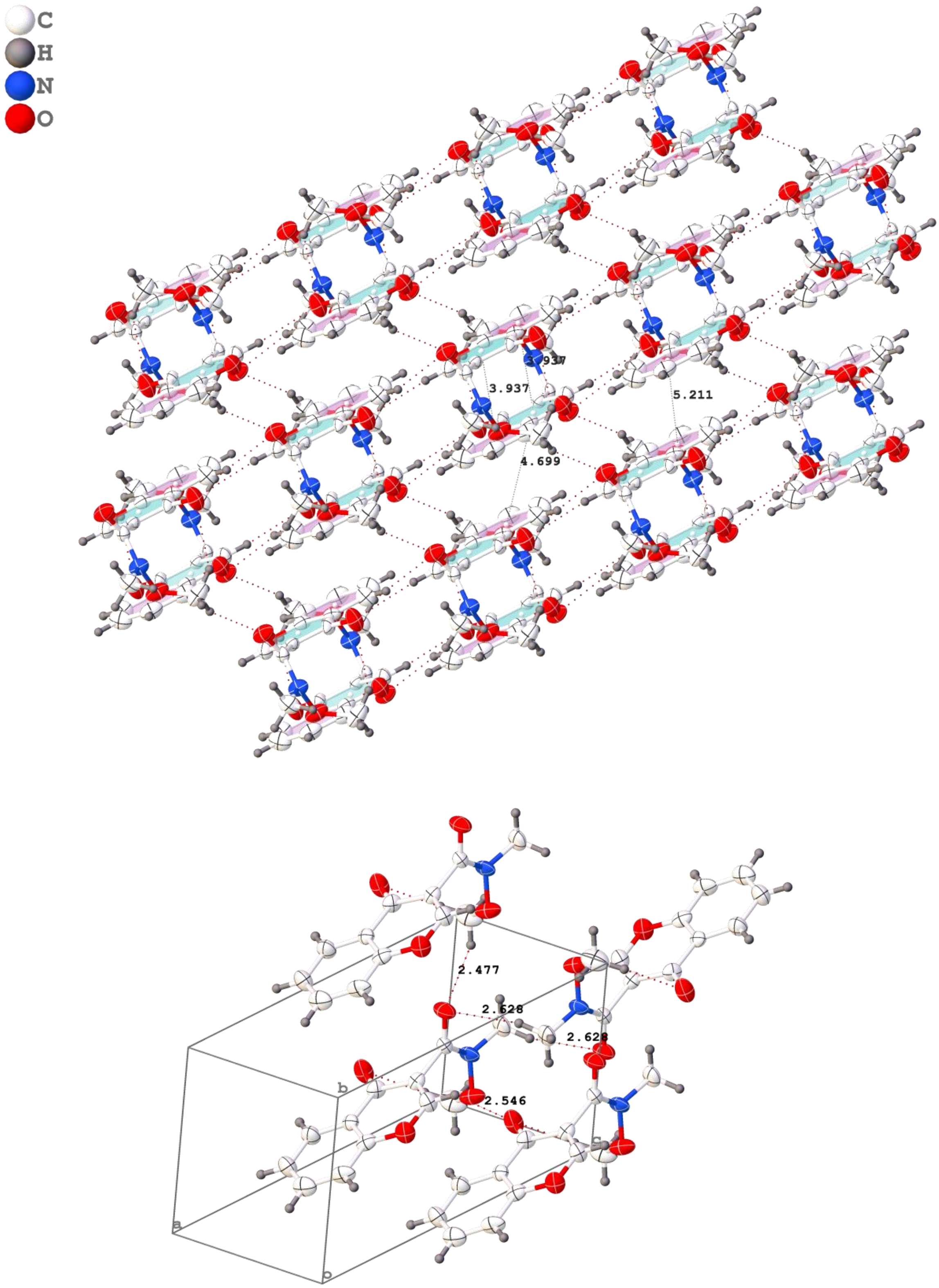

Figure S3. (Top): Crystal packing [View normal to (111)], (Bottom): fragment of crystal packing of (**2a**), showing thermal displacement ellipsoids, drawn at the 50% probability level. Red dashed lines highlight the inter-/intramolecular hydrogen-bonds. Black stippled lines indicate *face-to-face* (π···π) aromatic stackings. The indicated values showed the interplanar (*centroide-to-centroide*) distances.

The supramolecular structure of **2a** revealed perfect tapes of chromones, and inversion-related molecules connected primarily *via* π···π interactions with an interplanar distance (*centroid-to-centroid*) of 3.937 Å (Table S2) that enables '*face-to-face*' π···π stackings between the aromatic platforms. [11] Conversely, there was any evidence of CH···**π** stacking interactions. The arrangement of the rings is shown in Figure S3. Interestingly, the molecules are linked by weak intermolecular hydrogen bonds (2.54-2.62 Å) which are propagated along the *a*-, *b*-, and *c*-axis (Figure S3, Bottom) providing the final distorted chequerboard-like pattern.

Table S2. Geometric parameters for some selected inter/intramolecular hydrogen bonds and short interactions (Å, °) for (**2a**), and (**3s**).

Crystals	*Inter/Intramolecular hydrogen bonds*					*Short interactions* (Å)	
	A…H—D	D—H	H…A	D…A	∠D—H…A	π···π	CH···π
(2a)	CO)^{i}···H-CH_2)^{i} Chromo	0.960	2.586	3.398	142.49	3.937-4.699	-
	CO)^{ii}···H-CH_2)^{i} Chromo	0.960	2.477	3.371	154.86		
(3s)	CO)^{i} ···HN)^{i} Enaminone	0.927	2.075	3.747	128.20	3.617-3.991	3.228-3.695
	CO)^{ii} ···HN)^{i} Enaminone	0.927	2.449	3.310	154.52		

Symmetry codes : **2a** [(i) 3−x, −y, 1−z; (ii) 3−x, 1−y, 1−z]; **3s** [(i)-1/2+x, 1/2−y, 1/2+z; (ii) 3/2−x, -1/2+y, 3/2−z].

On the other hand, as depicted in Figure S5, title compound *3-((pyridin-2-ylamino)methylene)chromane-2,4-dione* **3s** crystallized in the monoclinic space group $P2_1/n$. The crystal structure of **3s** is solved and refined with four coumarin-enamine molecules, adopting their thermodynamically stable **Z**-ketoenamine isomer, and no stereoisomer counterpart (**E**) has been observed. The moderate intramolecular hydrogen-bonding interaction across the enaminone fragment ($d_{(C=O\cdots H-N)} \approx$ 2.07 Å, (Table S2), that could be seen at a glance, is a crucial hydrogen-bonded bridge between both planar parts: chroman-2,4-dione and pyridine ring, which was previously found in similar structures.[12]

The formed pseudo-six-membered-ring system, stabilized by the presence of a resonance-assisted hydrogen-bonding (RAHB), as it is confirmed by the shortening distances of involved bonds, gives rise to a continuous π-electron delocalization across [···O=C−C=C−NH···] motif which consequently establishes an extended planarity for the structure, (Scheme S3).[13]

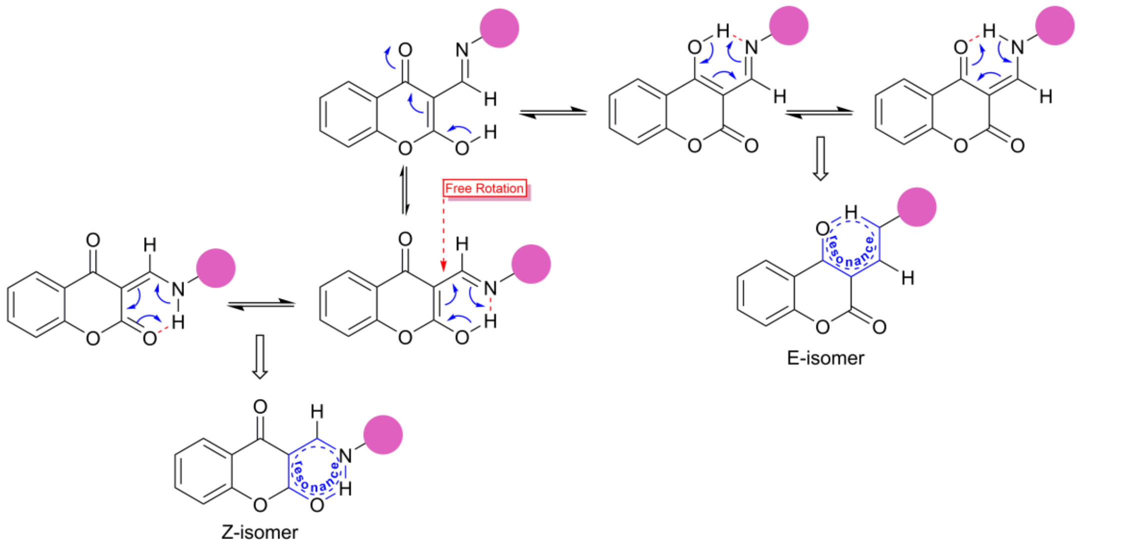

Scheme S3. Resonance-assisted hydrogen bonding (RAHB) pattern in chroman-2,4-diones **3**.

Further, the high dihedral angle observed in solid state between the protons in the =CH-NH fragment (∠CH-NH ~ 178.73°), is in accordance with the one estimated by ^{1}H NMR in solution using the measured coupling constant ($^{3}J_{=CH-NH}$ = 12.8 Hz) and Karplus type equation for vicinal coupling.[14]

Moreover, coumarin-enamine **3s** exhibits interesting supramolecular features including intermolecular hydrogen-bonding network (Figure S5), involving two related rings that are of a '*syn*' form, and a π-stackings offset between aromatic platforms (Figure S4, Table S2) leading to longer and rigid pseudo-γ-structure. Indeed, the overall crystal packing is stabilized by a set of bifurcated weak hydrogen-bonding interactions between neighbouring molecules: C_{Ar}–H···O$)_{Coumarin}$ of 2.56 Å; C_{Ar}–H···O=C$)_{Coumarin}$ of 2.62 Å; and NH)···O=C$)_{Coumarin}$ of 2.44 Å. Furthermore, the crystal packing shows '*face-to-face*' π···π interactions connecting parallel molecules separated by 3.617-3.991 Å, and '*edge-to-face*' CH···π contacts between two adjacent rings in a zipper-type fashion, with an interplanar distance of (3.228-3.695 Å).[15]

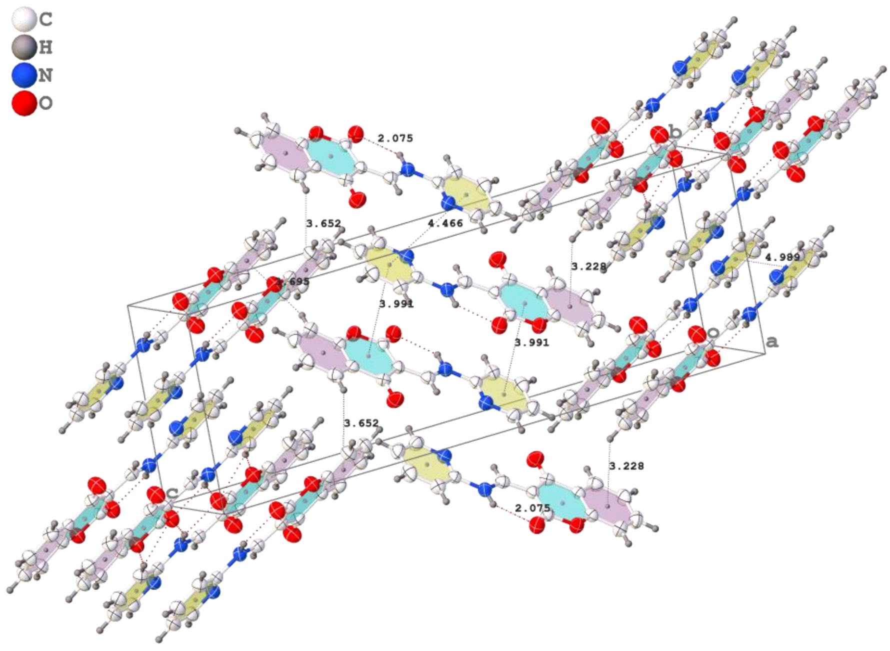

Figure S4. Fragment of Crystal Packing of (**3s**), [View normal to (011)]; showing thermal displacement ellipsoids, drawn at the 50% probability level. Red dashed lines indicate the inter-/intramolecular hydrogen-bonds. Black stippled lines indicate *face-to-face* (π···π) and *edge-to-face* (CH···π) aromatic stackings. The indicated values showed the interplanar (*centroide-to-centroide*) and intramolecular hydrogen-bonding distances. (The structure of the **3s** has been described in the literature: F.Borges, L.R.Gomes, J.N.Low (2014), CSD Communication (Private Communication)).

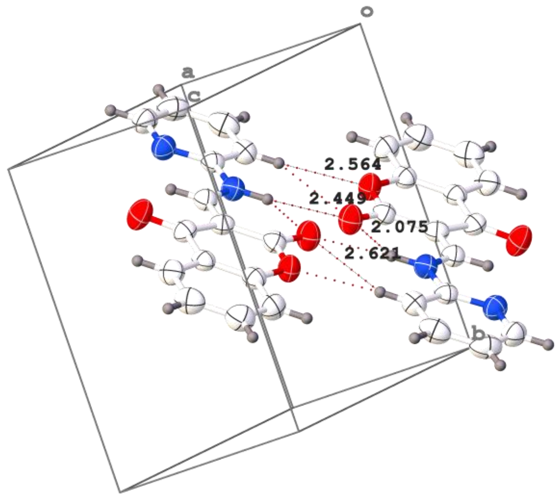

Figure S5. ORTEP view of dimer of **3s** [View normal to (111)]; showing hydrogen bonding array highlighting the intramolecular motif, and the bifurcated intermolecular interactions. Thermal displacement ellipsoids are drawn at the 50% probability level. The indicated values showed the intermolecular hydrogen-bonds distances.

References

(1) Kaushik, P.; Shakil, N. A.; Rana, V. S. Synthesis, Biological Evaluation, and QSAR Studies of 3-Iodochromone Derivatives as Potential Fungicides. *Front Chem* **2021**, *9*. DOI: http://dx.doi.org/10.3389/fchem.2021.636882.

(2) Schroder, N.; Lied, F.; Glorius, F. Dual Role of Rh(III) Catalyst Enables Regioselective Halogenation of (Electron-Rich) Heterocycles. *J Am Chem Soc* **2015**, *137* (4), 1448-1451. DOI: http://dx.doi.org/10.1021/jacs.5b00283.

(3) Mpitimpiti, A.; Petzer, J.; Petzer, A.; Jordaan, J.; Lourens, A. Synthesis and evaluation of chromone derivatives as inhibitors of monoamine oxidase. *Mol Divers* **2019**, *23* (4), 897-913. DOI: http://dx.doi.org/10.1007/s11030-019-09917-8.

(4) Ibrahim, M. Ring Transformation of Chromone-3-Carboxamide under Nucleophilic Conditions. *J Braz Chem Soc* **2013**, *24* (11), 1754-1763. DOI: http://dx.doi.org/10.5935/0103-5053.20130220.

(5) Neo, A.; Díaz, J.; Marcaccini, S.; Marcos, C. Conjugate addition of isocyanides to chromone 3-carboxylic acid: an efficient one-pot synthesis of chroman-4-one 2-carboxamides. *Organic & Biomolecular Chemistry* **2012**, *10* (17), 3406-3416. DOI: http://dx.doi.org/10.1039/c2ob07011a.

(6) Sosnovskikh, V. Y. New data on the reactivity of 2-unsubstituted 3-halochromones. *Chem Heterocycl Compd* **2020**, *56* (3), 243-254. DOI: http://dx.doi.org/10.1007/s10593-020-02653-0.

(7) Sheldrick, G. A short history of SHELX. *Acta Crystallogr. A* **2008**, *64*, 112-122. DOI: http://dx.doi.org/10.1107/S0108767307043930.

(8) Westrip, S. publCIF: software for editing, validating and formatting crystallographic information files. *J. Appl. Crystallogr.* **2010**, *43*, 920-925. DOI: http://dx.doi.org/10.1107/S0021889810022120.

(9) Dolomanov, O.; Bourhis, L.; Gildea, R.; Howard, J.; Puschmann, H. OLEX2: a complete structure solution, refinement and analysis program. *J. Appl. Crystallogr.* **2009**, *42,* 339-341. DOI: http://dx.doi.org/10.1107/S0021889808042726.

(10) Spek, A. Single-crystal structure validation with the program PLATON. *J. Appl. Crystallogr.* **2003**, *36*, 7-13. DOI: http://dx.doi.org/10.1107/S0021889802022112.

(11) (a) Riwar, L.; Trapp, N.; Kuhn, B.; Diederich, F. Substituent Effects in Parallel- Displaced pi-pi Stacking Interactions: Distance Matters. *Angew. Chem. Int. Ed.* **2017**, *56* (37), 11252-11257. DOI: http://dx.doi.org/10.1002/anie.201703744. (b) Janiak, C. A critical account on pi-

pi stacking in metal complexes with aromatic nitrogen-containing ligands. *J. Chem. Soc. Dalton Trans.* **2000**, (21), 3885-3896. DOI: http://dx.doi.org/10.1039/b003010o.

(12) Dimic, D.; Kaluderovic, G.; Avdovic, E.; Milenkovic, D.; Aivanovic, M.; Potocnak, I.; Samolova, E.; Dimitrijevic, M.; Saso, L.; Markovic, Z.; et al. Synthesis, Crystallographic, Quantum Chemical, Antitumor, and Molecular Docking/Dynamic Studies of 4-Hydroxycoumarin-Neurotransmitter Derivatives. *Int J Mol Sci* **2022**, *23* (2), 1001. DOI: http://dx.doi.org/10.3390/ijms23021001.

(13) Mahmudov, K. T.; Pombeiro, A. J. L. Resonance-Assisted Hydrogen Bonding as a Driving Force in Synthesis and a Synthon in the Design of Materials. *Chem Eur J* **2016**, *22* (46), 16356-16398. DOI: https://dx.doi.org/10.1002/chem.201601766.

(14) Karplus, M. Vicinal Proton Coupling in Nuclear Magnetic Resonance. *J Am Chem Soc* **1963**, *85* (18), 2870-2871. DOI: https://dx.doi.org/10.1021/ja00901a059.

(15) Davis, A.; Ihde, M.; Busenlehner, A.; Davis, D.; Mia, R.; Panella, J.; Fronczek, F.; Bonizzoni, M.; Wallace, K. Structural Features of a Family of Coumarin-Enamine Fluorescent Chemodosimeters for Ion Pairs. *Inorg Chem* **2021**, *60* (18), 14238-14252. DOI: http://dx.doi.org/10.1021/acs.inorgchem.1c01734.

4. Characterization of the synthesized compounds

N-Methoxy-N-methyl-4-oxo-4H-chromene-3-carboxamide (**2a**).

Yield: 93 mg (80 %); Pale yellow solid, m.p: 120-125 °C; R_f (95 % $CHCl_3$, 5 % EtOAc) 0.5. ^{1}H NMR (500 MHz, $CDCl_3$) δ 8.27 (d, 1H, *J* = 8.0 Hz), 8.14 (br s, 1H), 7.73 (t, 1H, *J* = 7.8 Hz), 7.51 (d, 1H, *J* = 8.4 Hz), 7.47 (t, 1H, *J* = 7.5 Hz), 3.72 (br s, 3H), 3.36 (s, 3H). ^{13}C{^{1}H} NMR (125 MHz, $CDCl_3$) δ 173.6, 156.1, 155.2, 155.0, 134.1, 126.3, 125.8, 124.5, 122.4, 118.2, 61.5, 32.6. ***Crystal data***: moiety formula: $C_{12}H_{11}NO_4$, M_r = 233.22 g/mol, Triclinic, *a* = 6.7870 (7) Å, *b* = 7.4840 (6) Å, *c* = 11.2710 (11) Å, *V* = 546.74 (9) Å^3, α = 90.160° (5), β =93.006° (5), γ = 106.962°(5), space group: P-1, *Z* =2, D_{calc} = 1.417 g/cm^3, no. of reflections measured 2075, $2\theta_{max}$ = 50.9°, the refinement converged at *R* = 0.129 and R_w = 0.329 for all data (**CCDC 2269045**). IR (KBr, ν (cm^{-1})): 3062 *w*, 3002 *w*, 2974 *w*, 2948 *w*, 1654 *vs*, 1635 *vs*, 1474 *s*, 1385 *m*, 1062 *w*, 844 *w*, 761 *m*.MS (EI): *m/z* (rel. int, %): 233 (1, [M$^+$]), 207 (6), 173 (100), 145 (5), 121 (50), 89 (8), 53 (13), 44 (3). HRMS (ESI-Q-TOF) m/z calcd for $C_{12}H_{11}NO_4$ [M+H]$^+$: 234.0761; found: 234.0764

N,N-Diethyl-4-oxo-4H-chromene-3-carboxamide (**2b**).

Yield: 64 mg (52 %); yellow solid, m.p: 82-85 °C; R_f (95 % $CHCl_3$, 5 % EtOAc) 0.3. ^{1}H NMR (500 MHz, $CDCl_3$) δ 8.26 (dd, 1H, *J* = 8.0, 1.1 Hz), 8.09 (s, 1H), 7.72 (dd, 1H, *J* = 7.75, 1.5 Hz), 7.50 (d, 1H, *J* = 8.4 Hz), 7.46 (t, 1H, *J* = 7.5 Hz), 3.58 (q, 2H, *J* = 7.1 Hz), 3.31 (q, 2H, *J* = 7.1 Hz), 1.28 (t, 3H, *J* = 7.1 Hz), 1.18 (t, 3H, *J* = 7.1 Hz). ^{13}C{^{1}H} NMR (125 MHz, $CDCl_3$) δ 174.1, 163.5, 156.2, 154.7, 134.1, 126.2, 125.7, 124.4, 124.1, 118.20, 43.3, 39.6, 14.3, 12.9. IR (KBr, ν (cm^{-1})): 3067 *w*, 2992 *w*, 2972 *w*, 2930 *w*, 1656 *vs*, 1621 *s*, 1466 *s*, 1388 *m*, 1093 *w*, 830 *w*, 766 *w*. MS (EI): *m/z* (rel. int, %): 245 (7, [M$^+$]), 207 (25), 173 (50), 151 (85), 146 (25), 121 (70), 97 (45), 72 (100), 57 (50), 44 (85). HRMS (ESI-Q-TOF) m/z calcd for $C_{14}H_{15}NO_3$ [M+H]$^+$: 246.1125; found: 246.1125.

Methyl (4-oxo-4H-chromene-3-carbonyl)prolinate (**2c**).

Yield: 92 mg (61 %); yellowish oil; R_f (50 % $CHCl_3$, 50 % EtOAc) 0.3. (***Rotameric Mixture***: **3/1**); ***Major Rotamer*:** ^{1}H NMR (500 MHz, $CDCl_3$) δ 8.24 (s, *overlapped*, 1H), 8.27 – 8.21 (m, *overlapped*, 1H), 7.72 (t, 1H, *J* = 7.8 Hz), 7.51 (d, 1H, *J* = 8.7 Hz), 7.46 (t, 1H, *J* = 7.6 Hz), 4.66 (dd, 1H, *J* = 8.7, 3.2 Hz), 3.79 (*s*, 3H), 3.70 (m, 1H), 3.58 (m, *overlapped*, 1H), 2.42 – 2.27 (m, *overlapped*, 1H), 2.11 – 2.17 (m, 1H), 2.00 – 2.08 (m, *overlapped*, 1H), 1.99 – 1.89 (m, *overlapped*, 1H). ^{13}C{^{1}H} NMR (126 MHz, $CDCl_3$) δ 173.4, 172.5, 163.2, 157.2, 156.1, 134.2,

126.2, 125.9, 124.6, 123.4, 118.3, 59.7, 52.3, 46.9, 31.1, 22.7. ***Minor Rotamer*****:** ^{1}H NMR (500 MHz, $CDCl_3$) δ 8.27 – 8.21 (m, *overlapped,* 1H), 8.15 (s, 1H), 7.72 (t, 1H, *J* = 7.8 Hz), 7.51 (d, 1H, *J* = 8.7 Hz), 7.46 (t, 1H, *J* = 7.6 Hz), 4.55 (dd, 1H, *J* = 8.3, 2.6 Hz), 3.79 (m, 1H, *overlapped,* 1H), 3.58 (s, *overlapped,* 3H), 3.58 (m, *overlapped,* 1H), 2.42 – 2.27 (m, *overlapped,* 1H), 2.24 – 2.10 (m, 1H), 2.00 – 2.08 (m, *overlapped*, 1H), 1.99 – 1.89 (m, *overlapped*, 1H). ^{13}C{^{1}H} NMR (125 MHz, $CDCl_3$) δ 173.7, 172.9, 163.2, 157.3, 155.9, 134.2, 126.2, 125.9, 124.4, 123.4, 118.3, 59.3, 52.3, 47.9, 29.4, 24.6. IR (KBr, ν (cm^{-1})): 3069 *w*, 2992 *w*, 2955 *w*, 2892 *w*, 1746 *m*, 1652 *m*, 1650 *vs*, 1648 *vs*, 1646 *vs*, 1644 *vs*, 1461 *m*, 1432 *m*, 1344 *w*, 1132 *w,* 764 *w*. MS (EI): *m/z* (rel. int, %): 301 (4, [M^+]), 242 (14), 207 (6), 173 (75), 128 (100), 121 (40), 96 (8), 68 (10), 53 (10). HRMS (ESI-Q-TOF) m/z calcd for $C_{16}H_{15}NO_5$ [M+H]$^+$: 302.1023; found: 302.1029.

N-Benzyl-N-methyl-4-oxo-4H-chromene-3-carboxamide (**2d**).

Yield: 100 mg (68 %); Yellow solid; m.p: 78-80 °C; R_f (99 % $CHCl_3$, 1 % EtOAc) 0.3. (***Rotameric Mixture***: **3/2**); ***Major Rotamer:*** ^{1}H NMR (500 MHz, $CDCl_3$) δ 8.29 (dd, *overlapped*, 1H, *J* = 7.9, 1.4 Hz), 8.24 (s, 1H), 7.74 (m, *overlapped*, 1H), 7.39 (m, *overlapped,* 6H), 7.23 (d, *overlapped*, 1H, *J* = 7.3 Hz), 4.82 (s, 2H), 2.95 (s, 3H). ^{13}C{^{1}H} NMR (126 MHz, $CDCl_3$) δ 173.8, 164.8, 156.2, 155.7, 136.4, 134.2, 128.9, 128.7(2xC_{Ar}), 127.9(2xC_{Ar}), 126.2, 125.9, 124.4, 123.5, 118.3, 51.1, 36.1. ***Minor Rotamer*****:** ^{1}H NMR (500 MHz, $CDCl_3$) δ 8.29 (dd, *overlapped,* 3H, *J* = 7.9, 1.4 Hz), 8.19 (s, 1H), 7.74 (m, *overlapped*, 1H), 7.39 (m, *overlapped,* 6H), 7.23 (d, *overlapped*, 1H, *J* = 7.3 Hz), 4.82 (s, 2H), 3.05 (s, 3H). ^{13}C{^{1}H} NMR (125 MHz, $CDCl_3$) δ 174.1, 164.8, 156.2, 156.1, 136.4, 134.3, 128.7, 128.9, 127.8, 127.4, 127.1, 126.2, 125.9, 124.4, 123.4, 118.2, 54.8, 33.2. IR (KBr, ν (cm^{-1})): 3066 *w*, 3024 *w*, 2914 *w*, 1646 *vs*, 1641 *vs*, 1616 *m*, 1461 *s*, 1388 *m*, 1074 *w*, 850 *w*, 774 *w*. MS (EI): *m/z* (rel. int, %): 293 (2, [M^+]), 267 (5), 207 (10), 199 (15), 173 (15), 146 (10), 120 (100), 91 (25), 65 (10), 42 (10). HRMS (ESI-Q-TOF) m/z calcd for $C_{18}H_{15}NO_3$ [M+H]$^+$: 294,1125; found: 294.1131.

8-(4-Oxo-4H-chromene-3-carbonyl)-8-azabicyclo[3.2.1]octan-3-one (**2e**).

Yield: 70 mg (47 %); yellowish oil; R_f (95 % $CHCl_3$, 5 % EtOAc) 0.3. ^{1}H NMR (500 MHz, $CDCl_3$) δ 8.34 (*s*, 1H), 8.27 (d, 1H *J* = 7.9 Hz), 7.77 (t, 1H, *J* = 7.8 Hz), 7.55 (d, 1H, *J* = 8.4 Hz), 7.51 (t, 1H, *J* = 7.6 Hz), 5.13 (s, 1H), 4.28 (s, 1H), 3.00 – 2.89 (td, 2H, *J* = 7.9, 3.5 Hz), 2.51 (d, 1H, *J* = 16.0 Hz), 2.39 (d, 1H, *J* = 16.0 Hz), 2.29 – 2.17 (m, 2H), 1.79 (d, 2H, *J* = 6.9 Hz).^{13}C{^{1}H} NMR (125 MHz, $CDCl_3$) δ 207.6, 174.1, 160.5, 157.7, 156.1, 134.4, 126.3, 126.1,

124.5, 122.5, 118.3, 55.55, 51.40, 49.97, 49.07, 29.87, 28.09. IR (KBr, ν (cm^{-1})): 3097 *w*, 2992 *w*, 2977 *w*, 2961 *w*, 1717 *m*, 1638 *vs*, 1613 *s*, 1461 *m*, 1445 *m*, 1128 *m*, 821 *w*, 758 *m*. MS (EI): *m/z* (rel. int, %): 297 (20, [M^+]), 269 (10), 207 (4), 173 (100), 121 (50), 96 (12), 82 (10), 53 (12), 44 (3). HRMS (ESI-Q-TOF) m/z calcd for $C_{17}H_{15}NO_4$ $[M+H]^+$: 298.1074; found: 298.1074.

N-(4-Hydroxyphenyl)-N-methyl-4-oxo-4H-chromene-3-carboxamide (**2f**).

Yield: 66 mg (45 %); yellow solid; m.p: 213-215 °C; R_f (94 % $CHCl_3$, 5 % EtOAc, 1 % MeOH) 0.3. ^{1}H NMR (500 MHz, DMSO-d_6) δ 9.48 (s, 1H), 8.42 (s, 1H), 7.94 (d, 1H, *J* = 7.9 Hz), 7.77 (t, 1H, *J* = 7.7 Hz), 7.58 (d, 1H, *J* = 8.4 Hz), 7.45 (t, 1H, *J* = 7.5 Hz), 7.06 (d, 2H, AA'BB, *J* = 8.6 Hz), 6.60 (d, 2H, AA'BB, *J* = 8.6 Hz), 3.28 (*s*, 3H). ^{13}C{^{1}H} NMR (125 MHz, DMSO-d_6) δ 173.3, 163.7, 156.8, 155.9, 155.8, 135.1, 134.99, 128.7($C_{AA'BB'}$), 126.3, 125.5, 123.9, 123.8, 118.9, 116.2, 115.8 ($C_{AA'BB'}$ + C_{Ar}), 37.4. IR (KBr, ν (cm^{-1}): 3444 *br w*, 3246 *m*, 3067 *w*, 3052 *w*, 2972 *w*, 1653 *vs*, 1627 *vs*, 1615 *s*, 1600 *s*, 1516 *vs*, 1469 *vs*, 1390 *m*, 1349 *m*, 1276 *m*, 1171 *m*, 920 *w*, 856 *w*, 754 *w*. MS (EI): *m/z* (rel. int, %): 295 (8, [M^+]), 281 (50), 253 (25), 221 (5), 207 (100), 191 (12), 173 (10), 96 (30), 73 (40), 57 (22), 43 (10). HRMS (ESI-Q-TOF) m/z calcd for $C_{17}H_{13}NO_4$ $[M+H]^+$: 296.0917; found: 296.0919.

N-Ethyl-4-oxo-N-(pyridin-4-ylmethyl)-4H-chromene-3-carboxamide (**2g**).

Yield: 111 mg (72 %); Pink solid, m.p: 135-140 °C; R_f (69 % $CHCl_3$, 30 % EtOAc, 1 % MeOH) 0.65. (***Rotameric Mixture***: **3/1**); ***<u>Major Rotamer</u>***: ^{1}H NMR (500 MHz, $CDCl_3$) δ 8.65 (d, 2H, AA'BB', *J* = 5.6 Hz), 8.30 (d, 1H, *J* = 7.9 Hz), 8.21 (s, 1H), 7.77 (t, 1H, *J* = 7.8 Hz), 7.55 (d, 1H, *J* = 8.4 Hz), 7.52 (d, *overlapped*, 2H, AA'BB', *J* = 4.9 Hz), 7.22 (d, 1H, *J* = 5.2 Hz), 4.84 (s, 2H), 3.36 (q, 2H, *J* = 7.1 Hz), 1.17 (t, 3H, *J* = 7.1 Hz). ^{13}C{^{1}H} NMR (125 MHz, $CDCl_3$) δ 174.2, 164.8, 156.24, 155.2, 148.99(2x$C_{AA'BB'}$), 147.5, 134.5, 126.1 (2x$C_{AA'BB'}$), 126.1, 123.2, 122.5, 122.5, 118.4, 46.9, 43.8, 14.1. ***<u>Minor Rotamer</u>***: ^{1}H NMR (500 MHz, $CDCl_3$) δ 8.59 (d, 2H, AA'BB', *J* = 5.3 Hz), 8.24 (d, 1H, *J* = 7.8 Hz), 8.15 (s, 1H), 7.73 (t, 1H, *J* = 7.4 Hz), 7.52 (d, *overlapped*, 2H, AA'BB', *J* = 4.9 Hz), 7.49 (d, 1H, *J* = 9.5 Hz), 7.46 (d, 1H, *J* = 7.5 Hz), 4.54 (s, 2H), 3.55 (q, 2H, *J* = 7.1 Hz), 1.26 (t, 3H, *J* = 7.1 Hz). ^{13}C{^{1}H} NMR (126 MHz, $CDCl_3$) δ 174.1, 164.7, 156.1, 155.99, 149.96(2x$C_{AA'BB'}$), 146.6, 134.5, 134.4, 126.0, 124.28(2x$C_{AA'BB'}$), 122.2, 118.4, 118.3, 51.2, 40.6, 12.3. IR (KBr, ν (cm^{-1}): 3044 *w*, 2987 *w*, 2982 *w*, 2930 *w*, 1654 *vs*, 1629 *vs*, 1464 *s*, 1103 *m*, 774 *s*. MS (EI): *m/z* (rel. int, %): 308 (10, [M^+]), 253 (20), 214 (20), 207 (70), 173 (35), 135 (100), 121 (30), 73 (27), 57 (10), 44 (10). HRMS (ESI-Q-TOF) m/z calcd for $C_{18}H_{16}N_2O_3$ $[M+H]^+$: 309.1234; found: 309.1236.

4-Oxo-N,N-bis(pyridin-2-ylmethyl)-4H-chromene-3-carboxamide (**2h**).

Yield: 95 mg (51 %); Magenta waxy solid, R_f (68 % $CHCl_3$, 30 % EtOAc, 2 % MeOH) 0.3. ^1H NMR (500 MHz, $CDCl_3$) δ 8.53 (t, 2H, *J* = 5.5 Hz), 8.37 (s, 1H), 8.25 (d, 1H, *J* = 7.7 Hz), 7.82 (s, 2H), 7.70 (t, 1H, *J* = 7.5 Hz), 7.64 (t, 1H, *J* = 7.5 Hz), 7.46 (d, 1H, *J* = 8.0 Hz), 7.44 (d, 1H, *J* = 7.5 Hz), 7.18-7.24 (m, 3H), 4.92 (s, 2H), 4.65 (s, 2H). $^{13}C\{^1H\}$ NMR (125 MHz, $CDCl_3$) δ 174.6, 165.9, 156.3, 156.1, 155.8, 149.7, 148.4, 137.9, 136.9, 134.3, 126.0, 125.9, 124.3, 122.8, 122.7, 122.5, 122.3, 122.2, 118.3, 53.9, 50.2. IR (KBr, ν (cm^{-1})): 3067 *w*, 2992 *w*, 2972 *w*, 2930 *w*, 1654 *s*, 1629 *vs*, 1467 *s*, 1388 *m*, 1128 *w*, 758 *w*.MS (EI): *m/z* (rel. int, %): 371 (1, [M^+]), 341(20), 281(50), 253 (25), 207 (100), 173 (20), 135 (20), 93 (15), 73 (50), 44 (20). HRMS (ESI-Q-TOF) m/z calcd for $C_{22}H_{17}N_3O_3$ $[M+H]^+$: 372.1343; found: 372.1330.

N-Methoxy-4-oxo-4H-chromene-3-carboxamide (**2i**).

Yield: 11 mg (10 %); Off-white solid, m.p: 114-116 °C; R_f (99 % $CHCl_3$, 1 % EtOAc) 0.2. ^1H NMR (500 MHz, $CDCl_3$) δ 11.55 (br s, 1H), 9.00 (s, 1H), 8.30 (d, 1H, *J* = 7.9 Hz), 7.80 (t, 1H, *J* = 7.5 Hz), 7.60 (d, 1H, *J* = 8.5 Hz), 7.54 (t, 1H, *J* = 7.6 Hz), 3.92 (s, 3H). $^{13}C\{^1H\}$ NMR (125 MHz, $CDCl_3$) δ 176.3, 162.2, 160.9, 156.1, 134.9, 126.5, 126.3, 123.9, 118.4, 115.5, 64.7. IR (KBr, ν (cm^{-1})): 3492 *br w*, 3278 *m*, 3066 *w*, 2942 *w*, 1679 *vs*, 1616 *s*, 1464 *vs*, 1394 *m*, 1045 *w*, 859 *w*, 777 *w*. MS (EI): *m/z* (rel. int, %): 219 (1, [M^+]), 207 (8), 189 (100), 121 (50), 92 (30), 64 (10), 44 (20). HRMS (ESI-Q-TOF) m/z calcd for $C_{11}H_9NO_4$ $[M+H]^+$: 220.0604; found: 220.0604.

Methyl (4-oxo-4H-chromene-3-carbonyl)alaninate (**2o**).

Yield: 5 mg (3 %); Off-white solid, m.p: 100-102 °C; R_f (94 % $CHCl_3$, 6 % EtOAc) 0.4. ^1H NMR (500 MHz, $CDCl_3$) δ 9.81 (d, 1H, *J* = 6.0 Hz), 8.99 (s, 1H), 8.34 (dd, 1H, *J* = 8.0, 1.4 Hz), 7.82 – 7.76 (m, 1H), 7.59 (d, 1H, *J* = 8.4 Hz), 7.54 (t, 1H, *J* = 7.6 Hz), 4.80 (p, 1H, *J* = 7.2 Hz), 3.81 (s, 3H), 1.59 (d, 3H, *J* = 7.2 Hz). $^{13}C\{^1H\}$ NMR (125 MHz, $CDCl_3$) δ 177.1, 172.9, 162.47, 162.44, 156.1, 134.6, 126.4, 126.3, 124.3, 118.4, 115.5, 52.4, 48.2, 18.2. IR (KBr, ν (cm^{-1})): 3465 *br w*, 3292 *w*, 3246 *w*, 2954 *w*, 2919 *w*, 1749 *s*, 1674 *vs*, 1618 *s*, 1539 *m*, 1469 *s*, 1303 *m*, 1215 *m*, 1180 *w*, 774 *w*. MS (EI): *m/z* (rel. int, %): 275 (1, [M^+]), 216 (75), 207 (5), 173 (100), 121 (30), 97 (7), 57 (8), 44 (10). HRMS (ESI-Q-TOF) m/z calcd for $C_{14}H_{13}NO_5$ $[M+H]^+$: 276.0866; found: 276.0864.

4-Oxo-N-phenyl-4H-chromene-3-carboxamide (**2r**).

Yield: 5 mg (4 %); Pale yellow solid, m.p: 164-166 °C; R_f (99 % $CHCl_3$, 1 % EtOAc) 0.5. ^1H NMR (500 MHz, $CDCl_3$) δ 11.44 (br s, 1H), 9.12 (s, 1H), 8.37 (d, 1H, *J* = 6.8 Hz), 7.83 (t, 1H, *J* = 8.5 Hz), 7.78 (d, 2H, *J* = 7.8 Hz), 7.63 (d, 1H, *J* = 8.4 Hz), 7.58 (t, 1H, *J* = 7.6 Hz), 7.40 (t, 2H, *J* = 7.9 Hz), 7.18 (t, 1H, *J* = 7.4 Hz). $^{13}C\{^1H\}$ NMR (125 MHz, $CDCl_3$) δ 177.4, 162.9, 160.7, 156.2, 138.0, 134.8, 129.0, 126.5, 126.3, 124.5, 124.1, 120.6, 118.5, 116.1. IR (KBr, ν (cm^{-1})): 3475 *br w*, 3234 *w*, 3196 *w*, 3088 *w*, 2924 *w*, 2854*w*, 1683 *vs*, 1619 *m*, 1584 *m*, 1552 *s*, 1438 *m*, 1311 *m*, 1118 *w*, 760 *s*. MS (EI): *m/z* (rel. int, %): 265 (58, [M^+]), 236 (1), 207 (6), 173 (100), 145 (5), 121 (50), 89 (10), 65 (10), 53 (15). HRMS (ESI-Q-TOF) m/z calcd for $C_{16}H_{11}NO_3$ $[M+H]^+$: 266.0812; found: 266.0813.

4-Oxo-N-(pyridin-4-yl)-4H-chromene-3-carboxamide (**2u**).

Yield: 113 mg (85 %); dark pink solid; m.p: 205-210 °C; R_f (70 % $CHCl_3$, 30 % EtOAc) 0.3. ^1H NMR (500 MHz, $CDCl_3$) δ 11.70 (s, 1H), 9.10 (s, 1H), 8.58 (d, 2H, AA'BB', *J* = 4.5 Hz), 8.37 (d, 1H, *J* = 8.0 Hz), 7.85 (t, 1H, *J* = 7.8 Hz), 7.70 (d, 2H, AA'BB', *J* = 5.6 Hz), 7.65 (d, 1H, *J* = 8.4 Hz), 7.60 (t, 1H, *J* = 7.6 Hz). $^{13}C\{^1H\}$ NMR (125 MHz, $CDCl_3$) δ 177.3, 163.3, 161.6, 156.2(2x$C_{AA'BB'}$), 150.7, 144.9, 135.2, 126.8, 126.3, 123.98, 118.6, 115.5, 114.5(2x$C_{AA'BB'}$). IR (KBr, ν (cm^{-1})): 3443 *br m*, 3091 *w*, 3041 *w*, 2914 *w*, 2854*w*, 1689 *vs*, 1619 *vs*, 1603 *vs*, 1575 *s*, 1546 *m*, 1464 *m*, 1391 *m*, 1115 *w*, 821 *w*, 755 *m*. MS (EI): *m/z* (rel. int, %): 266 (60, [M^+]), 207 (90), 173 (100), 121 (45), 73 (40), 53 (12), 44 (75). HRMS (ESI-Q-TOF) m/z calcd for $C_{15}H_{10}N_2O_3$ $[M+H]^+$: 267.0764; found: 267.0763.

*(**Z** or **E**)-3-((Methoxyamino)methylene)chromane-2,4-dione* (**3i**).

Yield: 77 mg (70 %); yellow solid, m.p: 122-124 °C; R_f (99 % $CHCl_3$, 1 % EtOAc) 0.7. (**100 % of *Z*** or ***E***): ^1H NMR (500 MHz, $CDCl_3$) δ 12.53 (br s, 1H), 8.46 (s, 1H), 7.94 (d, 1H, *J* = 7.8 Hz), 7.60 (t, 1H, *J* = 7.8 Hz), 7.33 (dd, 2H, *J* = 13.1, 8.0 Hz), 4.00 (s, 3H). $^{13}C\{^1H\}$ NMR (125 MHz, $CDCl_3$) δ 166.3, 161.2, 153.4, 148.2, 133.6, 124.4, 124.1, 116.9, 115.3, 96.2, 62.8. IR (KBr, ν (cm^{-1})): 3085 *w*, 3005 *w*, 2980 *w*, 2936 *w*, 2822 *w*, 1725 *vs*, 1711 *vs*, 1631 *s*, 1556 *m*, 1496 *w*, 1458 *w*, 1217 *w*, 1036 *s*, 910 *w*, 756 *w*. MS (EI): *m/z* (rel. int, %): 219 (1, [M^+]), 203 (45), 173 (45), 121 (70), 109 (100), 97 (70), 57 (85), 44 (65). HRMS (ESI-Q-TOF) m/z calcd for $C_{11}H_9NO_4$ $[M+H]^+$: 220.0604; found: 220.0598.

(Z/E)-3-((tert-Butylamino)methylene)chromane-2,4-dione (**3j**).

Yield: 49 mg (40 %); Pale yellow solid, m.p: 72-74 °C; R_f (97 % $CHCl_3$, 3 % EtOAc) 0.4. (***Mixture of Z/E*: 3/1**) ***Major isomer*:** ^{1}H NMR (500 MHz, $CDCl_3$) δ 12.35 (br s, 1H), 8.50 (d, 1H, *J* = 14.5 Hz), 8.03 (dd, 1H, *J* = 7.8, 1.5 Hz), 7.68 – 7.50 (m, *overlapped*, 1H), 7.36 – 7.12 (m, *overlapped*, 2H + C<u>H</u>Cl_3 *residual peak*), 1.50 (s, 9H). ^{13}C{^{1}H} NMR (126 MHz, $CDCl_3$) δ 181.2, 164.1, 158.11, 154.9, 134.1, 125.5, 123.9, 120.7, 117.2, 96.6, 55.5, 29.5. ***Minor isomer*:** ^{1}H NMR (500 MHz, $CDCl_3$) δ 10.59 (br s, 1H), 8.68 (d, 1H, *J* = 15.4 Hz), 8.12 (dd, 1H, *J* = 7.8, 1.5 Hz), 7.68 – 7.50 (m, *overlapped*, 1H), 7.36 – 7.12 (m, *overlapped*, 2H + C<u>H</u>Cl_3 *residual peak*), 1.50 (s, 9H). ^{13}C{^{1}H} NMR (125 MHz, $CDCl_3$) δ 178.5, 165.2, 156.4, 154.7, 134.1, 126.4, 124.1, 120.9, 117.2, 96.6, 55.6, 29.6. IR (KBr, ν (cm^{-1})): 3075 *w*, 2977 *w*, 2980 *w*, 2936 *w*, 2822 *w*, 1714 *m*, 1695 *m*, 1622 *s*, 1613 *m*, 1464 *m*, 1436 *w*, 1356 *w*, 1198 *w*, 761 *w*. MS (EI): *m/z* (rel. int, %): 245 (45, [M$^+$]), 231 (30), 207 (70), 188 (35), 136 (50), 121 (40), 73 (25), 57 (30), 44 (100). HRMS (ESI-Q-TOF) m/z calcd for $C_{14}H_{15}NO_3$ [M+H]$^+$: 246.1125; found: 246.1120.

(Z/E)-3-((Benzylamino)methylene)chromane-2,4-dione (**3k**).

Yield: 70 mg (50 %); Yellow solid, m.p: 165-167 °C; R_f (98 % $CHCl_3$, 2 % EtOAc) 0.5. (***Mixture of Z/E*: 3/1**) ***Major isomer*:** ^{1}H NMR (500 MHz, $CDCl_3$) δ 12.17 (s, 1H), 8.52 (d, 1H, *J* = 13.7 Hz), 8.03 (d, 1H, *J* = 7.0 Hz), 7.59 (t, *overlapped*, 1H, *J* = 7.1 Hz), 7.42 (m, *overlapped*, 3H), 7.33 (d, *overlapped*, 2H, *J* = 7.0 Hz), 7.30 – 7.25 (t, *overlapped*, 2H + C<u>H</u>Cl_3 *residual peak*), 4.73 (d, 2H, *J* = 4.3 Hz). ^{13}C{^{1}H} NMR (126 MHz, $CDCl_3$) δ 181.5, 163.9, 162.3, 154.9, 138.5, 134.4, 129.35(2xC_{Ar}), 128.9, 127.76(2xC_{Ar}), 125.7, 124.1, 120.6, 117.3, 97.3, 54.6. ***Minor isomer*:** ^{1}H NMR (500 MHz, $CDCl_3$) δ 10.51 (s, 1H), 8.70 (d, 1H, *J* = 13.2 Hz), 8.12 (br s, 1H), 7.59 (t, *overlapped*, 1H, *J* = 7.1 Hz), 7.42 (m, *overlapped*, 3H), 7.33 (d, *overlapped*, 2H, *J* = 7.0 Hz), 7.30 – 7.25 (m, *overlapped*, 2H + C<u>H</u>Cl_3 *residual peak*), 4.73 (d, 2H, *J* = 4.3 Hz). ^{13}C{^{1}H} NMR (125 MHz, $CDCl_3$) δ 176.2, 160.8, 159.4, 154.8, 137.5, 134.5, 129.3, 128.9, 127.7, 125.9, 124.2, 120.6, 117.1, 102.8, 54.6. IR (KBr, ν (cm^{-1})): 3256 *br w*, 3037 *w*, 2936 *w*, 1717 *s*, 1635 *vs*, 1467 *vs*, 1435 *m*, 1391 *m*, 1198 *m*, 1081 *w*, 751 *w*. MS (EI): *m/z* (rel. int, %): **279** (90, [M$^+$]), 262 (25), 207 (70), 188 (25), 142 (30), 121 (25), 91 (100), 73 (25), 65 (25), 44 (25). HRMS (ESI-Q-TOF) m/z calcd for $C_{17}H_{13}NO_3$ [M+H]$^+$: 280.0968; found: 280.0968.

(Z/E) 3-((Phenethylamino)methylene)chromane-2,4-dione (**3l**).

Yield: 80 mg (55 %); Peach solid, m.p: 175-178 °C; R_f (98 % $CHCl_3$, 2 % EtOAc) 0.4. (***Mixture of Z/E*: 7/3**) ***Major isomer*:** ^{1}H NMR (500 MHz, $CDCl_3$) δ 11.92 (br s, 1H), 8.28 (d, 1H, *J* =

13.9 Hz), 8.04 (d, 1H, *overlapped,* *J* = 7.6 Hz), 7.58 (t, *overlapped,* 1H, *J* = 7.2 Hz), 7.37 (t, *overlapped,* 2H, *J* = 7.4 Hz), 7.21-7.31 (m, *overlapped,* 5H), 3.81 (m, *overlapped,* 2H), 3.04 (t, *overlapped,* 2H, *J* = 7.0 Hz). $^{13}C\{^1H\}$ NMR (126 MHz, $CDCl_3$) δ 181.3, 163.8, 162.4, 154.9, 136.5, 134.3, 129.1(2xC_{Ar}), 128.7(2xC_{Ar}), 127.4, 125.7, 123.9, 120.6, 117.3, 96.9, 52.4, 36.7. ***Minor isomer*: ** ^1H NMR (500 MHz, $CDCl_3$) δ 10.31 (s, 1H), 8.48 (d, 1H, *J* = 14.6 Hz), 8.11 (d, 1H, *J* = 7.2 Hz), 7.58 (t, *overlapped,* 1H, *J* = 7.2 Hz), 7.37 (t, *overlapped,* 2H, *J* = 7.4 Hz), 7.21-7.31 (m, *overlapped,* 5H), 3.81 (m, *overlapped,* 2H), 3.04 (t, *overlapped,* 2H, *J* = 7.0 Hz). $^{13}C\{^1H\}$ NMR (125 MHz, $CDCl_3$) δ 178.5, 165.2, 160.8, 154.9, 136.5, 134.3, 129.1(2xC_{Ar}), 128.7(2xC_{Ar}), 127.4, 126.4, 124.3, 123.9, 120.6, 96.9, 52.4, 36.7. IR (KBr, ν (cm^{-1})): 3177 *w*, 3063 *w*, 3021 *w*, 2949 *w*, 1714 *s*, 1632 *vs*, 1461 *vs*, 1432 *m*, 1381 *w*, 1188 *w*, 1074 *w*, 568 *w*. MS (EI): *m/z* (rel. int, %): 293 (25, [M^+]), 281 (50), 253 (25), 207 (100), 203 (80), 175 (75), 135 (20), 121 (35), 73 (40), 57 (25), 44 (20). HRMS (ESI-Q-TOF) m/z calcd for $C_{18}H_{15}NO_3$ $[M+H]^+$: 294.1125; found: 294.1119.

*(**Z/E**)-3-((Cyclopentylamino)methylene)chromane-2,4-dione* (**3m**).

Yield: 69 mg (54 %); Off-white solid, m.p: 115-117 °C; R_f (99 % $CHCl_3$, 1 % EtOAc) 0.5. (***Mixture of Z/E*: 3/1**) ***Major isomer*:** ^1H NMR (500 MHz, $CDCl_3$) δ 12.02 (s, 1H), 8.46 (d, 1H, *J* = 13.9 Hz), 8.12 (d, 1H, *J* = 6.7 Hz), 7.58 (t, *overlapped*, 1H, *J* = 7.5 Hz), 7.27 (m, *overlapped*, 1H + C<u>H</u>Cl_3 *residual peak*), 4.01 (br s, *overlapped*, 1H), 2.15 (br s, *overlapped*, 2H), 1.92 – 1.83 (m, *overlapped*, 2H), 1.83 – 1.65 (m, *overlapped*, 4H). $^{13}C\{^1H\}$ NMR (126 MHz, $CDCl_3$) δ 181.3, 164.1, 160.6, 154.8, 134.2, 125.6, 120.6, 117.3(2xC_{Ar}), 96.6, 62.1, 33.7, 23.6. ***Minor isomer*:** ^1H NMR (500 MHz, $CDCl_3$) δ 10.32 (s, 1H), 8.65 (d, 1H, *J* = 14.8 Hz), 8.03 (d, 1H, *J* = 6.0 Hz), 7.58 (t, *overlapped*, 1H, *J* = 7.5 Hz), 7.27 (m, *overlapped*, 2H + C<u>H</u>Cl_3 *residual peak*), 4.01 (br s, *overlapped*, 1H), 2.15 (br s, *overlapped*, 2H), 1.92 – 1.83 (m, *overlapped*, 2H), 1.83 – 1.65 (m, *overlapped*, 4H). $^{13}C\{^1H\}$ NMR (125 MHz, $CDCl_3$) δ 181.3, 164.1, 159.3, 154.8, 134.2, 123.9, 120.6, 117.3(2xC_{Ar}), 96.6, 62.1, 33.7, 23.6. IR (KBr, ν (cm^{-1})): 3453 *w*, 3059 *w*, 2957 *w*, 2872 *w*, 1706 *s*, 1627 *vs*, 1612 *s*, 1460 *s*, 1353 *w*, 1180 *m*, 766 *m*. MS (EI): *m/z* (rel. int, %): 257 (100, [M^+]), 228 (10), 207 (85), 175 (35), 133 (20), 121 (75), 83 (75), 44 (50). HRMS (ESI-Q-TOF) m/z calcd for $C_{15}H_{15}NO_3$ $[M+H]^+$: 258.1125; found: 258.1120.

*Methyl (**Z/E**)-((2,4-dioxochroman-3-ylidene)methyl)glycinate* (**3n**).

Yield: 74 mg (57 %); Off-white solid, m.p: 175-177 °C; R_f (70 % $CHCl_3$, 30 % EtOAc) 0.5. (***Mixture of Z/E*: 2/1**) ***Major isomer*:** ^1H NMR (500 MHz, $CDCl_3$) δ 12.03 (br d, 2H, *J* = 7.4 Hz), 8.39 (d, 1H, *J* = 13.7 Hz), 8.07 (d, 1H, *J* = 7.8 Hz), 7.64 – 7.56 (m, *overlapped*, 1H), 7.34

– 7.19 (m, *overlapped*, 2H+ CHCl$_3$), 4.33 (d, 2H, *J* = 6.1 Hz), 3.87 (s, 3H). $^{13}C\{^1H\}$ NMR (126 MHz, CDCl$_3$) δ 181.7, 167.3, 163.6, 163.1, 154.9, 134.6, 125.9, 124.1, 120.4, 117.4, 98.2, 53.1, 50.7. ***Minor isomer*:** ^{1}H NMR (500 MHz, CDCl$_3$) δ 10.38 (br d, 1H, *J* = 8.0 Hz), 8.53 (d, 1H, *J* = 14.6 Hz), 8.12 (d, 1H, *J* = 7.8 Hz), 7.64 – 7.56 (m, *overlapped*, 1H), 7.34 – 7.19 (m, *overlapped*, 2H+ CHCl$_3$ *residual peak*), 4.37 (d, 2H, *J* = 6.1 Hz), 3.87 (s, 3H). $^{13}C\{^1H\}$ NMR (125 MHz, CDCl$_3$) δ 178.6, 167.4, 164.9, 161.5, 154.8, 134.6, 126.5, 124.3, 120.6, 120.4, 98.2, 50.6, 30.9. IR (KBr, ν (cm^{-1})): 3183 *w*, 3003 *w*, 2952 *w*, 2950 *w*, 1740 *s*, 1707 *s*, 1638 *v*s, 1470 *s*, 1432 *w*, 1319 *w*, 1198 *s*, 1094 *s*, 780 *m*. MS (EI): *m/z* (rel. int, %): 261 (100, [M$^+$]), 207 (14), 201 (70), 188 (95), 175 (63), 121 (80), 107 (20), 92 (18), 53 (20), 44 (28). HRMS (ESI-Q-TOF) m/z calcd for $C_{13}H_{11}NO_5$ [M+H]$^+$: 262.0710; found: 262.0702.

*Methyl (**Z/E**)-((2,4-dioxochroman-3-ylidene)methyl)alaninate* (**3o**).

Yield: 57 mg (41 %); Off-white solid, m.p: 138-140 °C; R$_f$ (94 % CHCl$_3$, 6 % EtOAc) 0.4. (***Mixture of Z**/**E***: **3**/**1**) ***Major isomer*:** ^{1}H NMR (500 MHz, CDCl$_3$) δ 12.18 (br s, 1H), 8.43 (d, 1H, *J* = 13.9 Hz), 8.04 (dd, 1H, *J* = 7.8, 1.4 Hz), 7.62 – 7.55 (m, 1H), 7.30 – 7.21 (*p*, *J* = 7.2 Hz, 2H + CHCl$_3$ *residual peak*), 3.85 (s, 3H), 1.69 (d, *overlapped*, 6H, *J* = 7.5 Hz). $^{13}C\{^1H\}$ NMR (126 MHz, CDCl$_3$) δ 181.5, 170.2, 163.7, 161.1, 154.9, 134.5, 125.8, 124.1, 120.5, 117.3, 97.8, 57.7, 53.2, 18.8. ***Minor isomer*:** ^{1}H NMR (500 MHz, CDCl$_3$) δ 10.50 (br s, 1H), 8.58 (d, 1H, *J* = 14.7 Hz), 8.10 (dd, 1H, *J* = 7.8, 1.4 Hz), 7.62 – 7.55 (m, 1H), 7.30 – 7.21 (m, 2 H), 4.42 (p, 1H, *J* = 7.1 Hz), 3.84 (s, 3H), 1.71 (d, 6H, *overlapped*, *J* = 7.0 Hz). $^{13}C\{^1H\}$ NMR (125 MHz, CDCl$_3$) δ 178.6, 170.3, 164.9, 159.5, 154.8, 128.1, 126.4, 124.2, 120.7, 117.3, 97.8, 57.6, 53.2, 18.9. IR (KBr, ν (cm^{-1})): 3007 *w*, 2960 *w*, 1735 *s*, 1706 *vs*, 1633 *vs*, 1620 *s*, 1463 *vs*, 1434 *m*, 1221 *w*, 1127 *m*, 757 *w*. MS (EI): *m/z* (rel. int, %): 275 (55, [M$^+$]), 216 (100), 207 (25), 189 (25), 175 (35), 173 (18), 121 (85), 96 (20), 53 (18), 44 (30). HRMS (ESI-Q-TOF) m/z calcd for $C_{14}H_{13}NO_5$ [M+H]$^+$: 276.0866; found: 276.0861.

*Methyl (**Z/E**)-((2,4-dioxochroman-3-ylidene)methyl)valinate* (**3p**).

Yield: 91 mg (60 %); White-waxy solid; R$_f$ (95 % CHCl$_3$, 5 % EtOAc) 0.4. (***Mixture of Z**/**E***: **3**/**1**) ***Major isomer*:** ^{1}H NMR (500 MHz, CDCl$_3$) δ 12.34 – 12.04 (m, 1H), 8.34 (d, 1H, *J* = 13.8 Hz), 8.08 (d, 1H, *J* = 7.8 Hz), 7.61 (t, *overlapped*, 1H *J* = 7.5 Hz), 7.39–7.18 (m, *overlapped*, 2H+ CHCl$_3$ *residual peak*), 4.00 (dd, 1H, *J* = 9.5, 5.1 Hz), 3.86 (s, *overlapped*, 3H), 2.53 – 2.27 (m, *overlapped*, 1H), 1.06 (t, *overlapped*, 6H, *J* = 6.9 Hz). $^{13}C\{^1H\}$ NMR (125 MHz, CDCl$_3$) δ 181.7, 169.3, 163.8, 161.9, 154.9, 134.5, 125.8, 124.1, 120.5, 117.3, 97.8, 69.1, 52.9, 31.9, 19.1, 17.3. ***Minor isomer*:** ^{1}H NMR (500 MHz, CDCl$_3$) δ 10.60–10.37 (m, 1H), 8.47 (d, 1H *J* = 14.6

Hz), 8.12 (d, 1H *J* = 7.7 Hz), 7.61 (t, *overlapped*, 1H, *J* = 7.5 Hz), 7.39–7.18 (m, *overlapped*, 2H + C<u>H</u>Cl_3 *residual peak*), 4.06 (dd, 1H *J* = 9.5, 5.2 Hz), 3.86 (s, *overlapped*, 3H), 2.53 – 2.27 (m, *overlapped*, 1H), 1.06 (t, *overlapped*, 6H *J* = 6.9 Hz). $^{13}C\{^1H\}$ NMR (125 MHz, $CDCl_3$) δ 178.7, 169.4, 165.0, 160.2, 154.8, 134.6, 126.5, 124.2, 120.7, 117.4, 97.8, 68.8, 52.9, 31.9, 18.9, 17.4. IR (KBr, ν (cm^{-1})): 3495 *w*, 2961 *w*, 2958 *w*, 2936 *w*, 1750 *m*, 1711 *m*, 1625 *vs*, 1603 *m*, 1467 *s*, 1448 *w*, 1204 *w*, 765 *w*. MS (EI): *m/z* (rel. int, %): 303 (35, [M^+]), 288 (10), 260 (10), 244 (10), 200 (10), 175 (15), 121 (30), 70 (25), 44 (5). HRMS (ESI-Q-TOF) m/z calcd for $C_{16}H_{17}NO_5$ $[M+H]^+$: 304.1179; found: 304.1177.

*Methyl (**Z/E**)-2-(((2,4-dioxochroman-3-ylidene)methyl)amino)-2-phenylacetate* (**3q**).

Yield: 152 mg (90 %); White solid, m.p: 188-190 °C; R_f (95 % $CHCl_3$, 5 % EtOAc) 0.4. (***Mixture of Z/E*: 5/1**) ***<u>Major isomer</u>*:** ^1H NMR (500 MHz, $CDCl_3$) δ 12.71 (dd, 1H, *J* = 12.4, 5.6 Hz), 8.35 (d, 1H, *J* = 13.9 Hz), 8.08 (d, *overlapped*, 1H, *J* = 6.8 Hz), 7.59 (t, 1H, *overlapped*, *J* = 7.6 Hz), 7.46 (m, *overlapped*, 3H), 7.41 (d, *overlapped*, 2H, *J* = 7.2 Hz), 7.27 (m, *overlapped*, 2H), 5.31 (d, 1H, *J* = 6.8 Hz), 3.85 (s, 3H). $^{13}C\{^1H\}$ NMR (125 MHz, $CDCl_3$) δ 181.6, 168.7, 163.6, 161.1, 154.9, 134.6, 134.3, 129.7, 129.69 (2x$\underline{C}_{Ar}$), 129.67, 127.38 (2x$\underline{C}_{Ar}$), 125.9, 124.1, 120.5, 117.3, 98.3, 65.4, 53.5. ***<u>Minor isomer</u>*:** ^1H NMR (500 MHz, $CDCl_3$) δ 11.02 (dd, 1H, *J* = 13.8, 5.5 Hz), 8.49 (d, 1H, *J* = 14.7 Hz), 8.06 (d, *overlapped*, 1H, *J* = 6.8 Hz), 7.59 (t, *overlapped*, 1H, *J* = 7.6 Hz), 7.46 (m, *overlapped*, 3H), 7.41 (d, *overlapped*, 2H, *J* = 7.2 Hz), 7.27 (m, *overlapped*, 2H), 5.36 (d, *J* = 6.9 Hz, 1H), 3.85 (s, 3H). $^{13}C\{^1H\}$ NMR (125 MHz, $CDCl_3$) δ 178.6, 168.8, 164.8, 159.5, 154.8, 134.6, 134.4, 129.8, 129.69 (2x$\underline{C}_{Ar}$), 129.6, 127.38 (2x$\underline{C}_{Ar}$), 126.4, 124.2, 120.6, 117.4, 98.4, 65.2, 53.5. IR (KBr, ν (cm^{-1})): 3040 *w*, 2945 *w*, 1746 *m*, 1730 *vs*, 1629 *s*, 1588 *m*, 1474 *m*, 1445 *w*, 1249 *w*, 1106 *w*, 748 *w*. MS (EI): *m/z* (rel. int, %): 337 (8, [M^+]), 278 (100), 253 (20), 207 (70), 175 (25), 158 (20), 135 (15), 121 (30), 73 (30), 44 (35). HRMS (ESI-Q-TOF) m/z calcd for $C_{19}H_{15}NO_5$ $[M+H]^+$: 338.1023; found: 338.1026.

*(**Z/E**)-3-((Phenylamino)methylene)chromane-2,4-dione* (**3r**).

Yield: 106 mg (80 %); White solid, m.p: 154-156 °C; R_f (99 % $CHCl_3$, 1 % EtOAc) 0.5. (***Mixture of Z/E*: 2/1**) ***<u>Major isomer</u>*:** ^1H NMR (500 MHz, $CDCl_3$) δ 13.72 (d, 1H, *J* = 12.7 Hz), 8.93 (d, 1H, *J* = 13.6 Hz), 8.10 (dd, 1H, *J* = 7.8, 1.5 Hz), 7.68 – 7.59 (m, *overlapped*, 1H), 7.50 (t, *overlapped*, 2H, *J* = 7.8 Hz), 7.43 – 7.29 (m, *overlapped*, 5H). $^{13}C\{^1H\}$ NMR (125 MHz, $CDCl_3$) δ 181.9, 163.6, 154.9, 153.5, 137.7, 134.8, 130.2, 127.5, 125.9, 124.2, 120.4, 118.5, 117.4, 98.8. ***<u>Minor isomer</u>*:** ^1H NMR (500 MHz, $CDCl_3$) δ 11.96 (d, 1H, *J* = 12.9 Hz), 9.07 (d,

1H, J = 14.5 Hz), 8.16 (dd, 1H, J = 7.8, 1.6 Hz), 7.68 – 7.59 (m, *overlapped*, 1H), 7.50 (t, *overlapped*, 2H, J = 7.8 Hz), 7.43 – 7.29 (m, *overlapped*, 5H). $^{13}C\{^1H\}$ NMR (125 MHz, $CDCl_3$) δ 178.7, 165.2, 155.0, 154.7, 137.7, 134.7, 130.2, 127.4, 126.6, 124.4, 120.7, 118.5, 117.4, 98.8. IR (KBr, ν (cm^{-1})): 3164 *w*, 3005 *w*, 2980 *w*, 2936 *w*, 1683 *vs*, 1642 *vs*, 1581 *s*, 1464 *vs*, 1429 *vs*, 1318 *s*, 1299 *s*, 1286 *m*, 1207 *m*, 1116 *s*, 1001 *w*, 827 *w*, 761 *s*. MS (EI): *m/z* (rel. int, %): 265 (60, [M^+]), 236 (6), 207 (40), 173 (100), 144 (15), 121 (35), 117 (42), 77 (25), 44 (15). HRMS (ESI-Q-TOF) m/z calcd for $C_{16}H_{11}NO_3$ $[M+H]^+$: 266.0812; found: 266.0807.

(Z/E)-3-((Pyridin-2-ylamino)methylene)chromane-2,4-dione (**3s**).

Yield:109 mg (82 %); Pale yellow solid; m.p: 213-215 °C; R_f (95 % $CHCl_3$, 5 % EtOAc) 0.5. (***Mixture of Z/E*: 3/1**). ***Major isomer*:** ^1H NMR (500 MHz, $CDCl_3$) δ 13.61 (d, 1H, J = 11.1 Hz), 9.65 (d, 1H, J = 12.8 Hz,), 8.49 (1H, d, J = 4.6 Hz), 8.09 (d, 1H, J = 7.8 Hz), 7.81 (t, 1H, J = 7.7 Hz), 7.63 (t, 1H, J = 7.7 Hz), 7.31 (m, 2H), 7.24 (t, 1H, J = 6 Hz), 7.14 (d, 1H, J = 8.1 Hz). $^{13}C\{^1H\}$ NMR (125 MHz, $CDCl_3$) δ 182.4, 163.3, 155.1, 154.4, 149.4, 149.4, 139.1, 135.1, 126.1, 124.2, 122.1, 120.3, 117.5, 113.4, 99.8. ***Minor isomer*:** ^1H NMR (500 MHz, $CDCl_3$) δ 11.96 (d, 1H, J = 12.4 Hz), 9.75 (d, 1H, J = 13.7 Hz), 8.49 (d, 1H, J = 4.6 Hz), 8.17 (d, 1H, J = 7.8 Hz), 7.81 (t, 1H, J = 7.7 Hz), 7.63 (t, 1H, J = 7.7 Hz), 7.31 (m, 2H), 7.24 (t, 1H, J = 6 Hz), 7.14 (d, 1H, J = 8.1 Hz). $^{13}C\{^1H\}$ NMR (125 MHz, $CDCl_3$) δ 181.5, 163.0, 157.3, 152.8, 149.3, 149.2, 139.1, 134.9, 126.7, 124.5, 121.9, 120.7, 117.4, 113.2, 99.4. ***Crystal data*:** moiety formula: $C_{15}H_{10}N_2O_3$, M_r = 266.25 g/mol, Monoclinic, a = 4.9887 (4) Å, b = 8.8428 (6) Å, c = 27.0506 (18) Å, V = 1193.09(15) Å^3, α = 90° , β =91.110° (3), γ = 90°, space group: P 2_1/n, Z = *4*, D_{calc} = 1.485 g/cm^3, no. of reflections measured 2254, $2\theta_{max}$ = 50.9°, the refinement converged at R = 0.052 and R_w = 0.130 for all data (CCDC 2269046). IR (KBr, ν (cm^{-1})): 3461 *br w*, 3277 *w*, 1692 *s*, 1648 *vs*, 1607 *s*, 1562 *s*, 1464 *vs*, 1417 *vs*, 1321 *s*, 1290 *s*, 818 *w*, 761 *s*. MS (EI): *m/z* (rel. int, %): 266 (30, [M^+]), 238 (25), 207 (50), 173 (5), 121 (15), 79 (100), 73 (15), 44 (15). HRMS (ESI-Q-TOF) m/z calcd for $C_{15}H_{10}N_2O_3$ $[M+H]^+$: 267.0764; found: 267.0762.

(Z/E)-3-((Pyridin-3-ylamino)methylene)chromane-2,4-dione (**3t**).

Yield: 120 mg (90 %); pink solid; R_f (100 % EtOAc) 0.5. (***Mixture of Z/E*: 3/1**) ***Major isomer*:** ^1H NMR (500 MHz, $CDCl_3$) δ 13.75 (d, 1H, J = 12.1 Hz), 8.91 (d, 1H, J = 13.2 Hz), 8.71 (d, *overlapped*, 1H, J = 2.4 Hz), 8.60 (d, *overlapped*, 1H, J = 4.5 Hz), 8.08 (dd, 1H, J = 7.8, 1.2 Hz), 7.79 – 7.71 (m, 1H), 7.67 – 7.59 (td, 1H, J = 7.75, 1.5 Hz), 7.46 (dd, 1H, J = 8.2, 4.7 Hz), 7.37 – 7.24 (m, *overlapped*, 2H + C<u>H</u>Cl_3 *residuel peak*). $^{13}C\{^1H\}$ NMR (125 MHz, $CDCl_3$) δ

182.3, 163.2, 155.3, 155.1, 148.4, 141.1, 135.2, 126.7, 126.0, 125.0, 124.4, 124.4, 120.1, 117.5, 99.8. ***Minor isomer***: ^{1}H NMR (500 MHz, $CDCl_3$) δ 11.94 (d, 1H, *J* = 13.5 Hz), 9.03 (d, 1H, *J* = 14.2 Hz), 8.71 (d, 1H, *J* = 2.4 Hz), 8.60 (d, 1H, *J* = 4.5 Hz), 8.14 (dd, 1H, *J* = 7.8, 1.3 Hz), 7.79 – 7.71 (m, 1H), 7.67 – 7.59 (td, 1H, *J* = 7.75, 1.5 Hz), 7.46 (dd, 1H, *J* = 8.2, 4.7 Hz), 7.37 – 7.24 (m, *overlapped*, 2H + C<u>H</u>Cl_3 *residuel peak*). ^{13}C{^{1}H} NMR (125 MHz, $CDCl_3$) δ 178.6, 165.2, 154.7, 153.7, 148.3, 141.1, 135.1, 134.5, 125.9, 126.3, 125.2, 124.6, 120.5, 117.5, 99.9. IR (KBr, ν (cm^{-1})): 3452 *w*, 3205 *w*, 3063 *w*, 2927 *w*, 1689 *vs*, 1651 *vs*, 1607 *vs*, 1571 *m*, 1467 *vs*, 1306 *s*, 764 *w*. MS (EI): *m/z* (rel. int, %): 266 (100, [M^+]), 249 (25), 237 (15), 207 (75), 173 (95), 145 (15), 121 (50), 73 (20), 51 (14), 44 (20). HRMS (ESI-Q-TOF) m/z calcd for $C_{15}H_{10}N_2O_3$ $[M+H]^+$: 267.0764; found: 267.0767.

*(**Z/E**)-3-(((2-Aminophenyl)amino)methylene)chromane-2,4-dione* (**3v**).

Yield: 128 mg (89 %); yellow-orange solid; m.p: 218-220 °C, R_f (95 % $CHCl_3$, 5 % MeOH) 0.5. (***Mixture of Z/E: 3/1***) ***Major isomer:*** ^{1}H NMR (500 MHz, DMSO-d_6) δ 13.46 (d, 1H, *J* = 12.7 Hz), 8.75 (d, *overlapped*, 1H, *J* = 12.3 Hz), 8.02 (d, *overlapped*, 1H, *J* = 6.5 Hz), 7.71 (t, *overlapped*, 1H, *J* = 6.9 Hz), 7.50 (d, *overlapped*, 1H, *J* = 7.9 Hz), 7.45 – 7.32 (m, *overlapped*, 2H), 7.12 (t, 1H, *J* = 7.6 Hz), 6.94 (d, *overlapped*, 1H, *J* = 7.4 Hz), 6.78 (t, *overlapped*, 1H, *J* = 7.5 Hz), 5.31 (br s, 2H). ^{13}C{^{1}H} NMR (125 MHz, DMSO-d_6) δ 180.4, 162.8, 156.5, 154.8, 141.1, 135.2, 128.7, 126.1, 125.9, 124.7, 120.6, 120.4, 118.9, 118.2, 117.6, 98.4. ***Minor isomer:*** ^{1}H NMR (500 MHz, DMSO-d_6) δ 11.68 (br s, 1H), 8.75 (d, *overlapped*, 1H, *J* = 12.3 Hz), 8.02 (d, *overlapped*, 1H, *J* = 6.5 Hz), 7.71 (t, *overlapped*, 1H, *J* = 6.9 Hz), 7.50 (d, *overlapped*, 1H, *J* = 7.9 Hz), 7.45 – 7.32 (m, *overlapped*, 2H), 7.12 (t, *overlapped*, 1H, *J* = 7.6 Hz), 6.94 (d, *overlapped*, 1H, *J* = 7.4 Hz), 6.78 (t, *overlapped*, 1H, *J* = 7.5 Hz), 5.31 (br s, 2H). ^{13}C{^{1}H} NMR (125 MHz, DMSO-d_6) δ 177.6, 164.1, 155.6, 154.6, 141.4, 135.2, 128.7, 126.4, 126.3, 124.8, 121.1, 120.8, 118.7, 118.2, 117.7, 98.3. IR (KBr, ν (cm^{-1})): 3405 *w*, 3350 *w*, 3096 *w*, 2936 *w*, 2822 *w*, 1720 *vs*, 1625 *s*, 1606 *s*, 1562 *m*, 1474 *s*, 1436 *m*, 1201 *w*, 745 *w*. HRMS (ESI-Q-TOF) *m/z* calcd for $C_{16}H_{12}N_2O_3$ $[M+H]^+$: 281.0921; found: 281.0921.

*(**Z/E**)-3-(((2-Hydroxyphenyl)amino)methylene)chromane-2,4-dione* (**3w**).

Yield: 129 mg (92 %); orange solid, m.p: 290-292 °C, (*Recrystallized from* DMF); R_f (98 % $CHCl_3$, 2 % MeOH) 0.3. (***Mixture of Z/E*: 4/1**), ***Major isomer***: ^{1}H NMR (500 MHz, DMSO-d_6) δ(ppm) 13.75 (d, 1H, *J* = 12.7 Hz), 10.78 (br s, 1H), 8.96 (d, 1H, *J* = 12.8 Hz), 8.00 (dd, 1H, *J* = 7.7, 1.2 Hz), 7.77 (d, 1H, *J* = 7.6 Hz), 7.69 (t, 1H, *J* = 7.6 Hz), 7.36 (d, 1H, *J* = 7.4 Hz), 7.36 (d, *overlapped*, 1H, *J* = 7.5 Hz), 7.34 (t, *overlapped*, 1H, *J* = 9.5 Hz), 7.16 (t, 1H, *J* = 7.4 Hz),

7.03 (d, 1H, J = 7.9 Hz), 6.94 (t, 1H, J = 7.6 Hz). $^{13}C\{^1H\}$ NMR (125 MHz, DMSO-d_6) δ 180.7, 162.8, 154.7, 153.8, 148.2, 135.24(2x$\underline{C}_{Ar}$), 128.2, 125.9, 124.6, 120.55(2x$\underline{C}_{Ar}$), 117.5, 117.5, 116.5, 98.4. ***Minor isomer*:** ^1H NMR (500 MHz, DMSO-d_6) δ 12.11 (br s, 1H), 10.78 (br s, 1H), 9.04 (br s, 1H), 8.00 (dd, 1H, J = 7.7, 1.2 Hz), 7.77 (d, 1H, J = 7.6 Hz), 7.69 (t, 1H, J = 7.6 Hz), 7.36 (d, 1H, J = 7.4 Hz), 7.36 (d, *overlapped*, 1H, J = 7.5 Hz), 7.34 (t, *overlapped*, 1H, J = 9.5 Hz), 7.16 (t, 1H, J = 7.4 Hz), 7.03 (d, 1H, J = 7.9 Hz), 6.94 (t, 1H, J = 7.6 Hz). $^{13}C\{^1H\}$ NMR (125 MHz, DMSO-d_6) δ 180.7, 162.8, 154.7, 153.8, 148.2, 135.24(2x$\underline{C}_{Ar}$), 128.2, 125.9, 124.6, 120.55(2x$\underline{C}_{Ar}$), 117.5, 117.5, 116.5, 98.4. IR (KBr, ν (cm^{-1})): 3170 *w*, 1720 *s*, 1632 *vs*, 1600 *m*, 1552 *w*, 1486 *m*, 1467 *vs*, 1436 *s*, 1340 *m*, 752 *w*. HRMS (ESI-Q-TOF) m/z calcd for $C_{16}H_{11}NO_4$ $[M+H]^+$: 282.0761; found: 282.0760.

*(**Z**/**E**)-3-(((Benzo[d][1,3]dioxol-5-ylmethyl)amino)methylene)chromane-2,4-dione* (**3x**).

Yield: 97 mg (60 %); White solid, m.p: 175-178 °C; R_f (98 % $CHCl_3$, 2 % EtOAc) 0.3. (***Mixture of Z*/*E*: 3/1**) ***Major isomer*:** ^1H NMR (500 MHz, $CDCl_3$) δ 12.11 (s, 1H), 8.48 (d, 1H, J = 13.8 Hz), 8.02 (d, 1H, J = 7.4 Hz), 7.59 (t, *overlapped,* 1H, J = 7.3 Hz), 7.27 (*m*, *overlapped*, 2H+ C$\underline{H}$$Cl_3$ *residuel peak*), 6.84 (d, *overlapped*, 1H, J = 8.0 Hz), 6.79 (d, *overlapped*, 2H, J = 7.7 Hz), 6.01 (s, 2H), 4.62 (d, 2H, J = 4.9 Hz). $^{13}C\{^1H\}$ NMR (125 MHz, $CDCl_3$) δ 181.5, 163.8, 161.9, 154.9, 148.5, 148.2, 134.4, 128.0, 125.7, 124.0, 121.7, 120.5, 117.3, 108.8, 108.2, 101.5, 97.30, 54.40. ***Minor isomer*:** ^1H NMR (500 MHz, $CDCl_3$) δ 10.45 (s, 1H), 8.65 (d, 1H, J = 13.9 Hz), 8.12 (d, 1H, J = 5.3 Hz), 7.59 (t, *overlapped,* 1H, J = 7.3 Hz), 7.27 (m, *overlapped*, 2H+ C$\underline{H}$$Cl_3$ *residuel peak*), 6.84 (d, *overlapped*, 1H, J = 8.0 Hz), 6.79 (d, *overlapped*, 2H, J = 7.7 Hz), 6.01 (s, 2H), 4.65 (br s, *overlapped*, 2H). $^{13}C\{^1H\}$ NMR (125 MHz, $CDCl_3$) δ 178.6, 165.2, 160.4, 154.7, 148.5, 148.2, 134.4, 128.0, 126.4, 124.2, 121.7, 120.7, 117.3, 108.8, 108.2, 101.5, 97.3, 54.2. IR (KBr, ν (cm^{-1})): 3044 *w*, 2949 *w*, 2895 *w*, 1724 *vs*, 1642 *vs*, 1492 *m*, 1467 *s*, 1438 *m*, 1381 *m*, 1255 *s*, 1040 *m*, 928 *w*, 758 *w*. MS (EI): *m/z* (rel. int, %): **323** (55, $[M^+]$), 306 (12), 207 (75), 186 (20), 131 (100), 121 (10), 73 (30), 54 (10), 44 (5). HRMS (ESI-Q-TOF) m/z calcd for $C_{18}H_{13}NO_5$ $[M+H]^+$: 324.0866; found: 324.0871.

*(**Z**/**E**)-3-(((3,4-Dihydroxybenzyl)amino)methylene)chromane-2,4-dione* (**3y**).

Yield: 63 mg (40 %); Yellow solid, m.p: 233-235 °C; R_f (95 % $CHCl_3$, 5 % MeOH) 0.3. (***Mixture of Z*/*E*: 2/1**) ***Major isomer* :** ^1H NMR (500 MHz, DMSO-d_6) δ 12.00 – 11.67 (m, 1H), 9.06 (s, 1H), 9.02 (s, 1H), 8.55 (d, 1H, J = 14.6 Hz), 7.92 (d, 1H, J = 7.6 Hz), 7.65 (t, *overlapped*, 1H, J = 7.2 Hz), 7.35 – 7.26 (m, *overlapped*, 1H), 6.81 (s, *overlapped*, 2H), 6.75 (d, *overlapped*, 1H, J = 8.0 Hz), 6.68 (*d*, *overlapped*, 1H, J = 7.9 Hz), 4.65 (d, *overlapped*, 2H, J = 5.8 Hz).

$^{13}C\{^1H\}$ NMR (125 MHz, DMSO-d$_6$) δ 179.9, 163.2, 162.1, 154.7, 145.9, 145.8, 134.8, 127.6, 125.7, 124.4, 120.7, 119.8, 117.4, 116.2, 116.1, 96.3, 53.35. ***Minor isomer*** **:** ^1H NMR (500 MHz, DMSO-d$_6$) δ 10.69 – 10.47 (m, 1H), 9.05 (s, 1H), 9.01 (s, 1H), 8.65 (d, 1H, *J* = 15.7 Hz), 7.95 (d, 1H, *J* = 7.5 Hz), 7.65 (t, *overlapped*, 1H, *J* = 7.2 Hz), 7.35 – 7.26 (m, *overlapped*, 1H), 6.81 (s, *overlapped*, 2H), 6.75 (d, *overlapped*, 1H, *J* = 8.0 Hz), 6.68 (d, *overlapped*, 1H, *J* = 7.9 Hz), 4.65 (d, *overlapped*, 1H, *J* = 5.8 Hz). $^{13}C\{^1H\}$ NMR (125 MHz, DMSO-d$_6$) δ 177.5, 163.7, 160.5, 154.7, 145.9, 145.8, 134.8, 127.8, 126.2, 124.5, 120.9, 119.8, 117.5, 116.2, 116.1, 96.1, 53.1. IR (KBr, ν (cm^{-1})): 3414 *w*, 3250 *w*, 1685 *m*, 1629 *vs*, 1619 *s*, 1467 *m*, 1448 *w*, 1353 *w*, 1226 *w*, 1110 *w*, 758 *w*. HRMS (ESI-Q-TOF) m/z calcd for $C_{17}H_{13}NO_5$ [M+H]$^+$: 312.0866; found: 312.0872.

*(**Z**/**E**)-Diethyl((((2,4-dioxochroman-3-ylidene)methyl)amino)(phenyl)methyl)phosphonate* (**3z**).

Yield: 91 mg (44 %); White solid, m.p: 138-140 °C; R$_f$ (95 % $CHCl_3$, 5 % EtOAc) 0.2. (***Mixture of Z***/***E***: **3**/**1**) ***Major isomer*****:** $^{31}P\{^1H,^{13}C\}$ NMR (202,48 MHz, $CDCl_3$) δ 16.83. ***Minor isomer*****:** $^{31}P\{^1H,^{13}C\}$ NMR (202,48 MHz, $CDCl_3$) δ 21.10. ***Major isomer*****:** ^1H NMR (500 MHz, $CDCl_3$) δ 12.71 (br s, 1H), 8.49 (d, 1H, *J* = 13.3 Hz), 8.09 (d, *overlapped*, 1H, *J* = 8.2 Hz), 7.61 (t, *overlapped*, 1H, *J* = 6.6 Hz), 7.55 – 7.42 (m, *overlapped*, 5H), 7.25-7.31 (m, *overlapped*, 2H + C<u>H</u>Cl$_3$ *residuel peak*), 4.88 ((-*C<u>H</u>-), dd, *overlapped*, 1H, $^2J_{(-*C\underline{H}-P)}$ = 19.3, $^3J_{(-*CH-NH-)}$ = 8.5 Hz), 4.21 – 4.06 (m, 2H), 4.04 – 3.92 (m, 2H), 1.33 (t, 3H, *J* = 7.0 Hz), 1.26 (t, 3H, *J* = 7.0 Hz). $^{13}C\{^1H\}$ NMR (125 MHz, $CDCl_3$) δ 181.7, 163.5, 162.3, 162.2, 154.9, 134.6, 131.94 ((C_{Ar}), d, *overlapped*, $^2J_{(-\underline{C}Ar-*CH-P)}$ = 12 Hz), 129.3, 129.3, 127.9, 127.9, 125.9, 124.1, 120.4, 117.4, 98.3, 64.1 ((-*<u>C</u>H-), *d*, *overlapped*, $J_{(-*\underline{C}H-P)}$ = 27 Hz), 62.97, 61.75, 16.32, 16.28. ***Minor isomer*****:** ^1H NMR (500 MHz, $CDCl_3$) δ 10.93 (br s, 1H), 8.62 (d, 1H, *J* = 14.2 Hz), 8.11 (d, 1H, *J* = 8.6 Hz), 7.61 (t, *overlapped*, 1H, *J* = 6.6 Hz), 7.55 – 7.42 (m, 5H, *overlapped*), 7.25-7.31 (m, *overlapped*, 2H + C<u>H</u>Cl$_3$ *residuel peak*), 4.94 ((-*<u>C</u>H-), dd, 1H, $J_{(-*C\underline{H}-P)}$ = 19.7, $^3J_{(-*CH-NH-)}$ = 8.6 Hz), 4.21 – 4.06 (m, 2H), 4.04 – 3.92 (m, 2H), 1.33 (t, 3H, *J* = 7.0 Hz), 1.26 (t, 3H, *J* = 7.0 Hz). $^{13}C\{^1H\}$ NMR (125 MHz, $CDCl_3$) δ 178.5, 165.2, 160.6, 160.5, 154.8, 134.6, 131.99 ((C_{Ar}), d, *overlapped*, $^2J_{(-CAr-*\underline{C}H-P)}$ = 11.5 Hz), 129.4, 129.4, 128.03, 128.01, 126.5, 124.3, 120.7, 117.4, 98.3, 64.1 ((-*<u>C</u>H-), d, *overlapped*, $J_{(-*\underline{C}H-P)}$ = 27 Hz), 62.6, 61.4, 16.4, 16.4. IR (KBr, ν (cm^{-1})): 3051 *w*, 2983 *w*, 2946 *w*, 2902 *w*, 1703 *vs*, 1630 *vs*, 1595 *w*, 1460 *s*, 1329 *w*, 1200 *w*, 1049 *w*, 973 *w*, 760 *w*. HRMS (ESI-Q-TOF) m/z calcd for $C_{21}H_{22}NO_6P$ [M+H]$^+$: 416.1258; found: 416.1268.

*(**Z/E**)-3-(((2-(3,4-Dihydroxyphenyl)-2-hydroxyethyl)amino)methylene)chromane-2,4-dione* (**3a'**).

Yield: 77 mg (45 %); yellow solid, m.p: 198-200 °C; R_f (95 % $CHCl_3$, 5 % MeOH) 0.3. (***Mixture of Z/E*: 2/1**) ***Major isomer*:** ^{1}H NMR (500 MHz, DMSO-d_6) δ 11.71 (d, 1H, *J* = 14.0 Hz), 8.85 (s, *overlapped*, 2H), 8.42 (d, *overlapped*, 1H, *J* = 14.3 Hz), 7.96 (d, *overlapped*, 1H, *J* = 6.5 Hz), 7.67 (t, *overlapped*, 1H, *J* = 8 Hz), 7.32 (t, *overlapped*, 1H, *J* = 7 Hz) ,7.29 (d, *overlapped*, 1H, *J* = 8.5 Hz), 6.82 (s, *overlapped*, 1H), 6.72 (d, *overlapped*, 2H, *J* = 8.0 Hz), 6.66 (dd, *overlapped*, 1H, *J* = 7.9, 1.5 *Hz*), 5.67 (d, 1H, *J* = 3.6 Hz), 4.65 – 4.66 (m, *overlapped*, 1H), 3.76 – 3.78 (m, *overlapped*, 1H), 3.56 – 3.60 (m, *overlapped*, 1H). $^{13}C\{^1H\}$ NMR (125 MHz, DMSO-d_6) δ 179.9, 163.4, 161.8, 154.7, 145.5, 145.1, 134.7, 133.7, 125.8, 124.4, 120.8, 117.4, 117.3, 115.7, 114.0, 96.1, 71.17, 57.8. ***Minor isomer*:** ^{1}H NMR (500 MHz, DMSO-d_6) δ 10.41 (d, *J* = 13.9 Hz, 1H), 8.85 (s, *overlapped*, 2H), 8.55 (d, 1H, *J* = 14.9 Hz), 7.96 (d, *overlapped*, 1H, *J* = 6.5 Hz), 7.67 (t, *overlapped*, 1H, *J* = 8 *Hz*), 7.32 (t, *overlapped*, 1H, *J* = 8.5 Hz), 7.29 (d, *overlapped*, 1H, *J* = 8.5 Hz), 6.82 (s, *overlapped*, 1H), 6.72 (d, *overlapped*, 1H, *J* = 8.0 Hz), 6.66 (dd, *overlapped*, 1H, *J* = 7.9, 1.5 Hz), 5.64 (d, 1H, *J* = 3.6 Hz), 4.65 – 4.66 (m, *overlapped*, 1H), 3.76 – 3.78 (m, *overlapped*, 1H), 3.56 – 3.60 (m, *overlapped*, 1H). $^{13}C\{^1H\}$ NMR (125 MHz, DMSO-d_6) δ 177.5, 163.9, 163.2, 154.6, 145.5, 145.1, 134.8, 133.7, 126.2, 124.5, 121.0, 117.5, 117.3, 115.7, 114.0, 95.9, 71.1, 57.8. IR (KBr, ν (cm^{-1})): 3478 *br w*, 3205 *br w*, 1695 *m*, 1641 *vs*, 1632 v*s*, 1556 *w*, 1464 *m*, 1445 *w*, 1356 *w*, 1198 *w*, 751 *w*. HRMS (ESI-Q-TOF) m/z calcd for $C_{18}H_{15}NO_6$ $[M+H]^+$: 342.0972; found: 342.0976.

5. Copies of the ^1H and $^{13}C\{^1H\}$ NMR spectra of the synthesized compounds

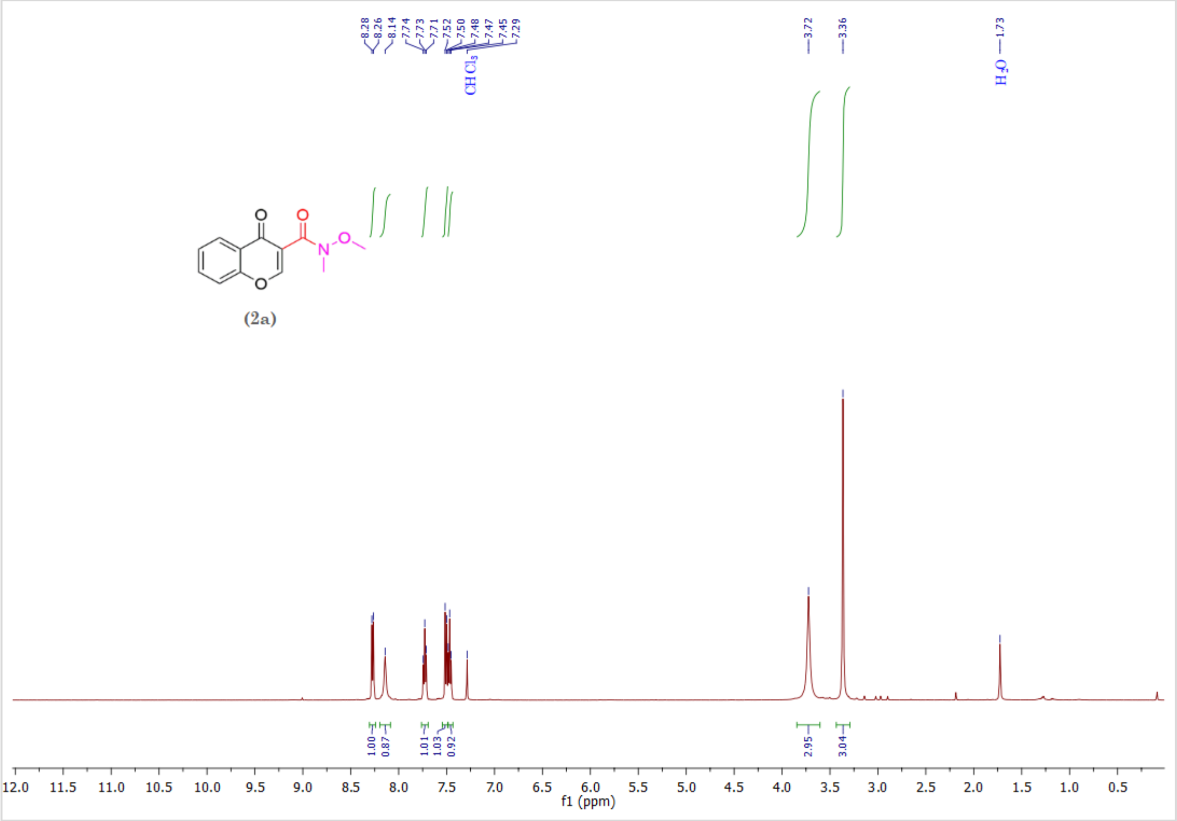

Figure S6. ^1H NMR spectrum of compound **2a** (500 MHz, $CDCl_3$)

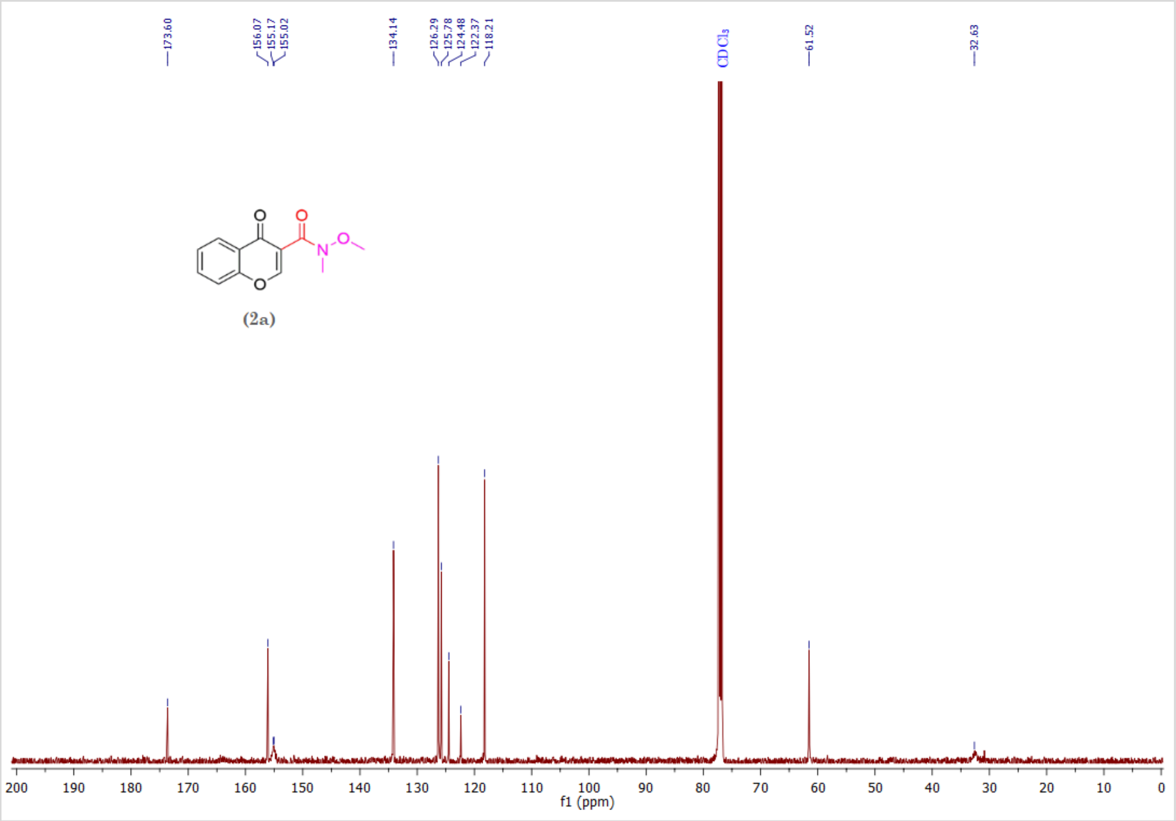

Figure S7. $^{13}C\{^1H\}$ NMR spectrum of compound **2a** (125 MHz, $CDCl_3$)

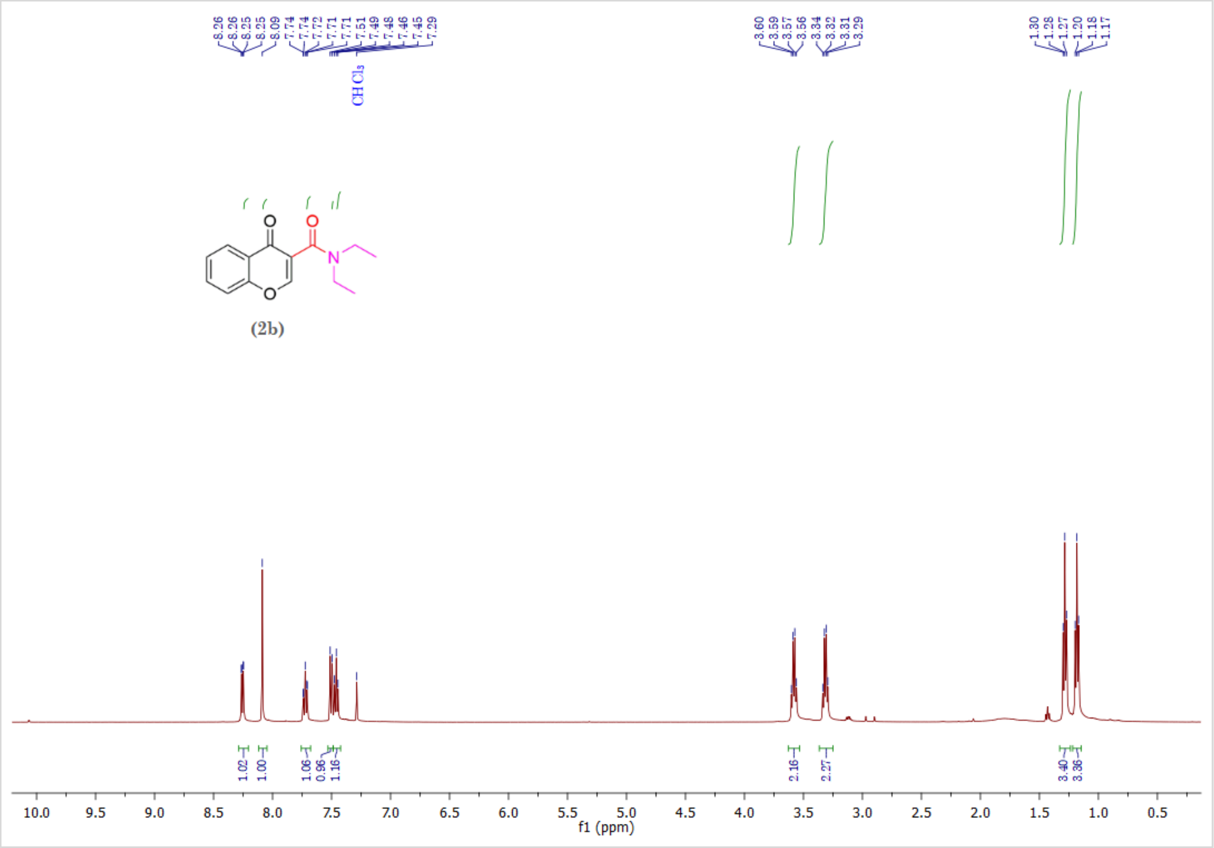

Figure S8. ^{1}H NMR spectrum of compound **2b** (500 MHz, $CDCl_3$)

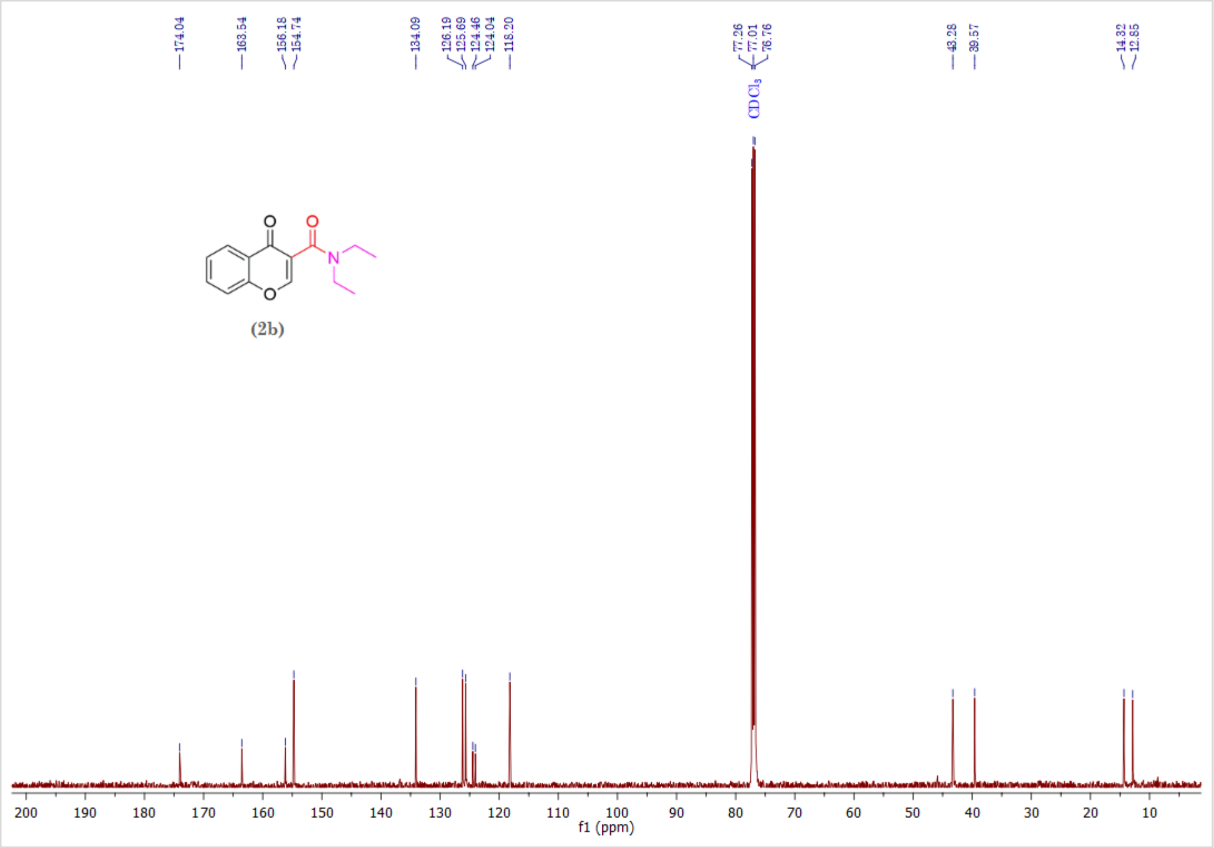

Figure S9. $^{13}C\{^1H\}$ NMR spectrum of compound **2b** (125 MHz, $CDCl_3$)

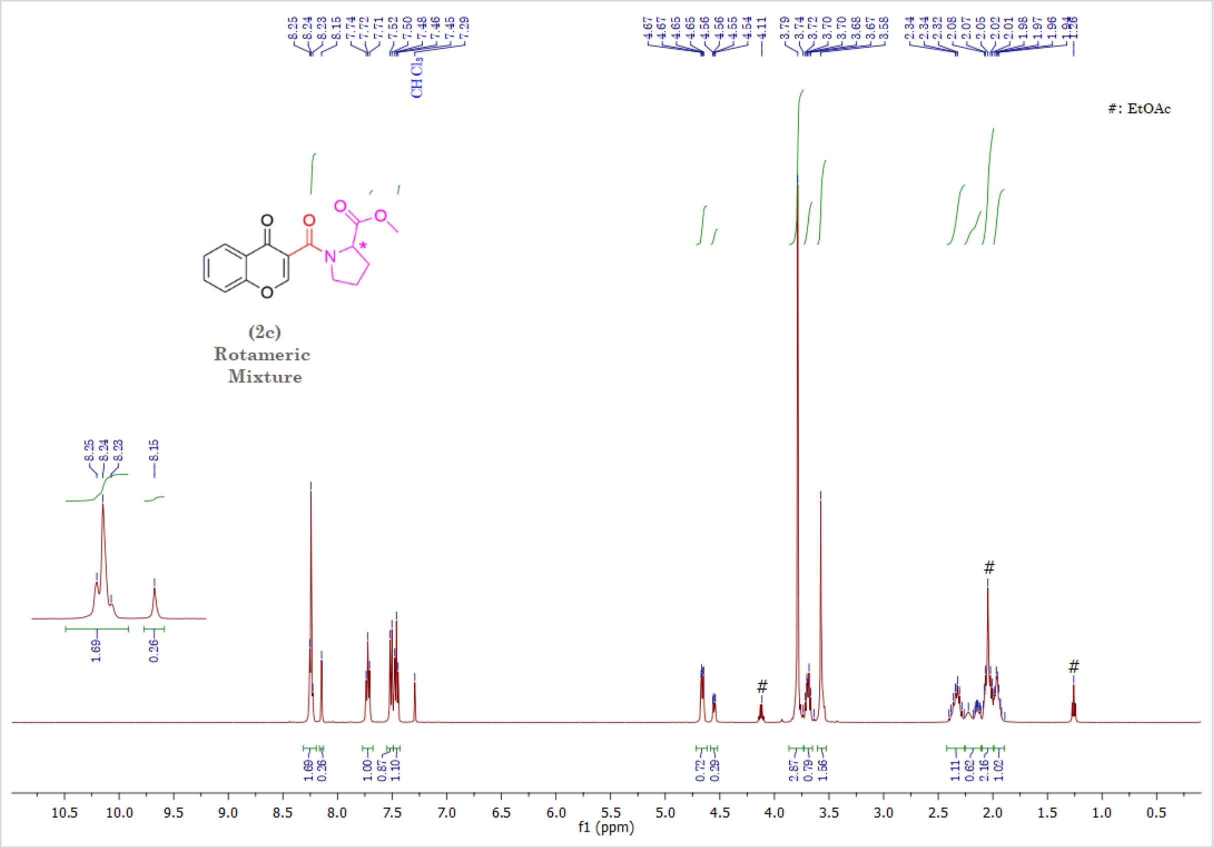

Figure S10. ^{1}H NMR spectrum of compound **2c** (500 MHz, $CDCl_3$)

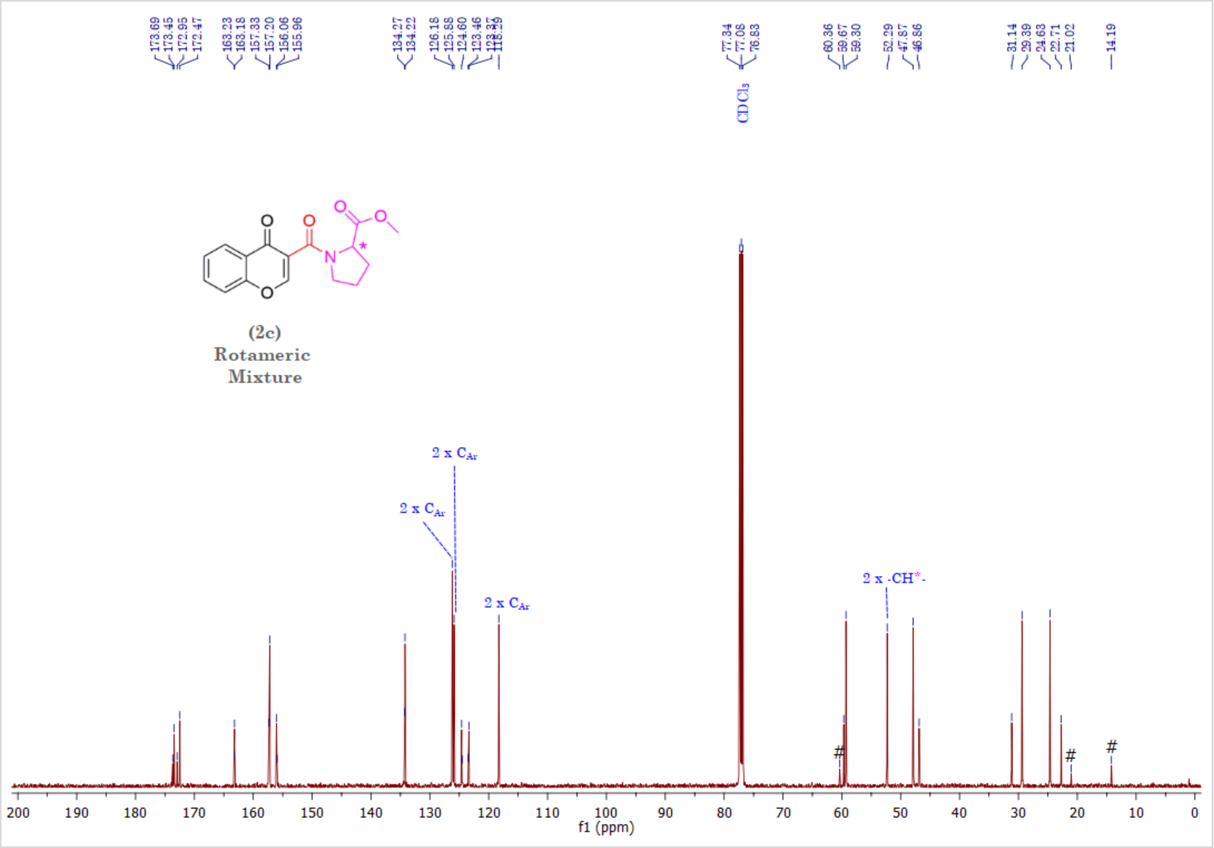

Figure S11. ^{13}C{^{1}H} NMR spectrum of compound **2c** (125 MHz, $CDCl_3$)

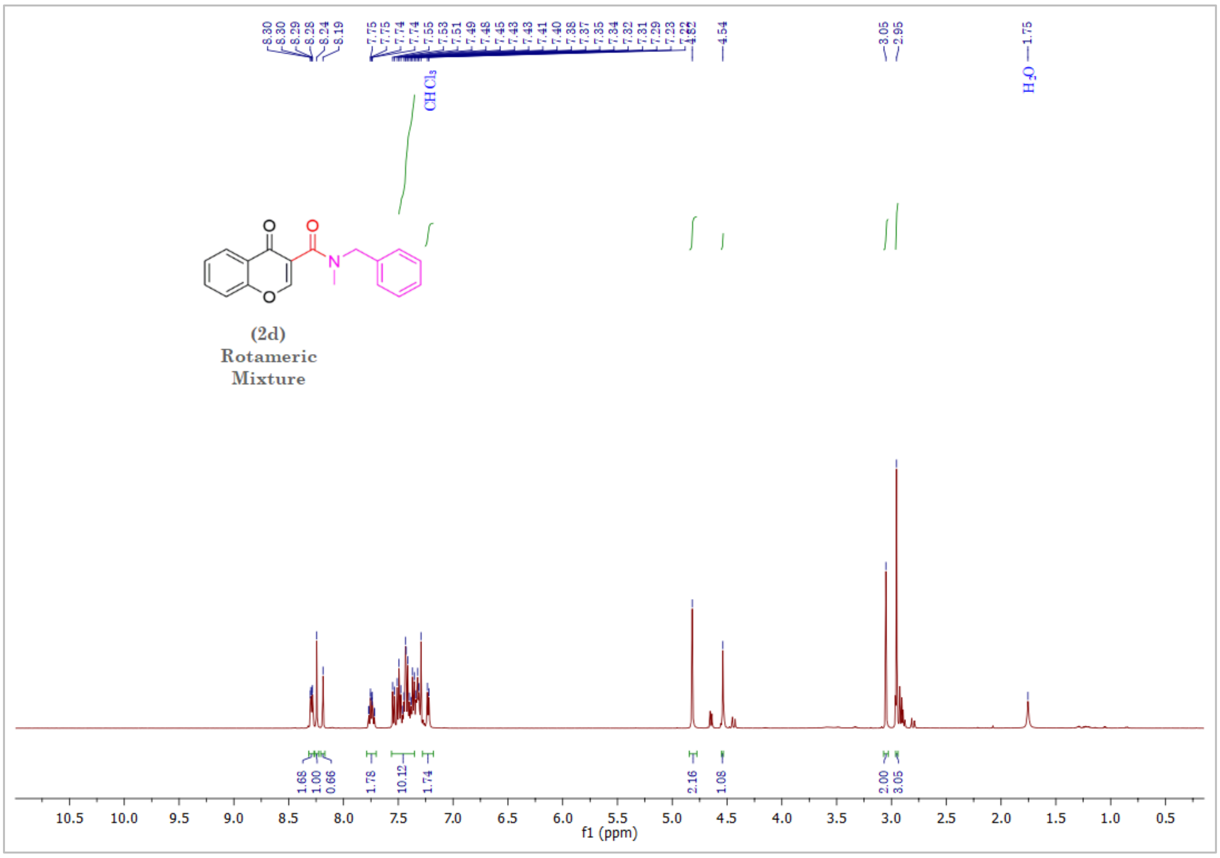

Figure S12. ^{1}H NMR spectrum of compound **2d** (500 MHz, $CDCl_3$)

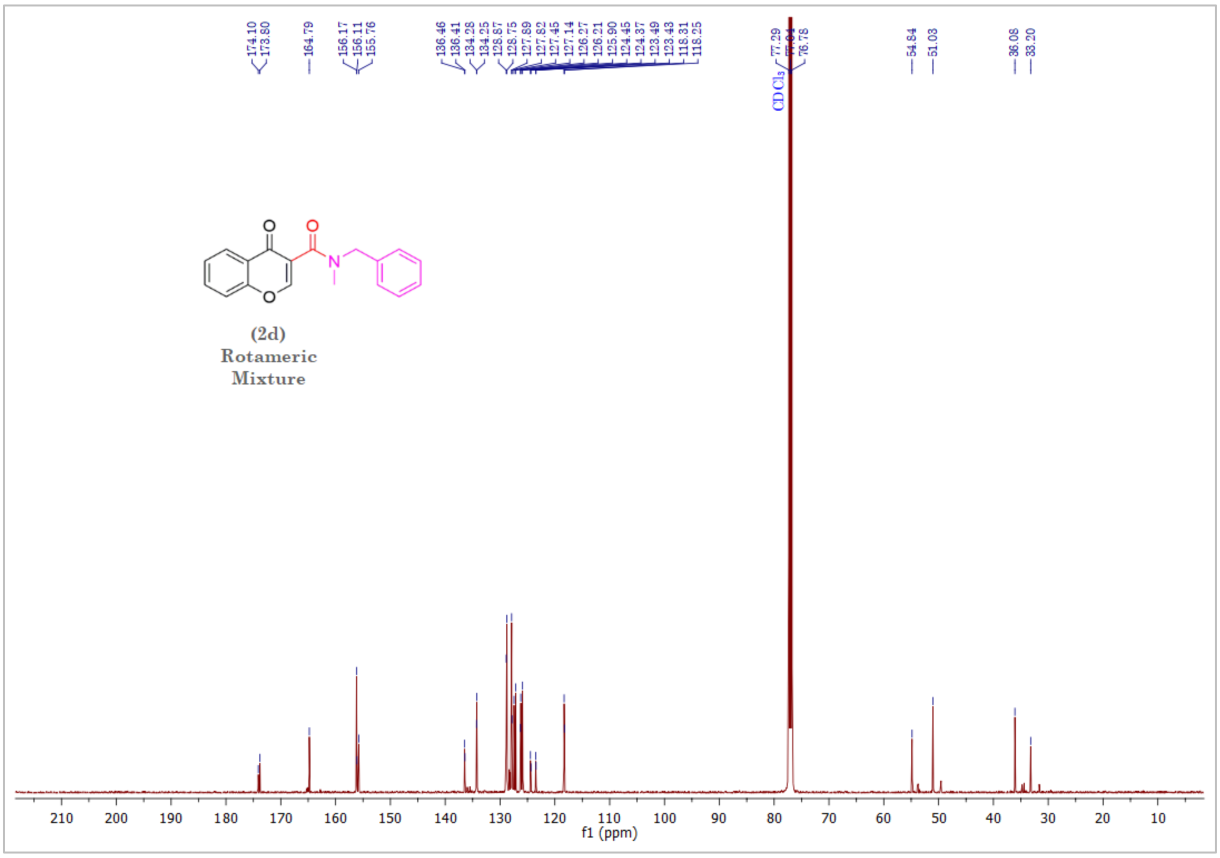

Figure S13. $^{13}C\{^{1}H\}$ NMR spectrum of compound **2d** (125 MHz, $CDCl_3$)

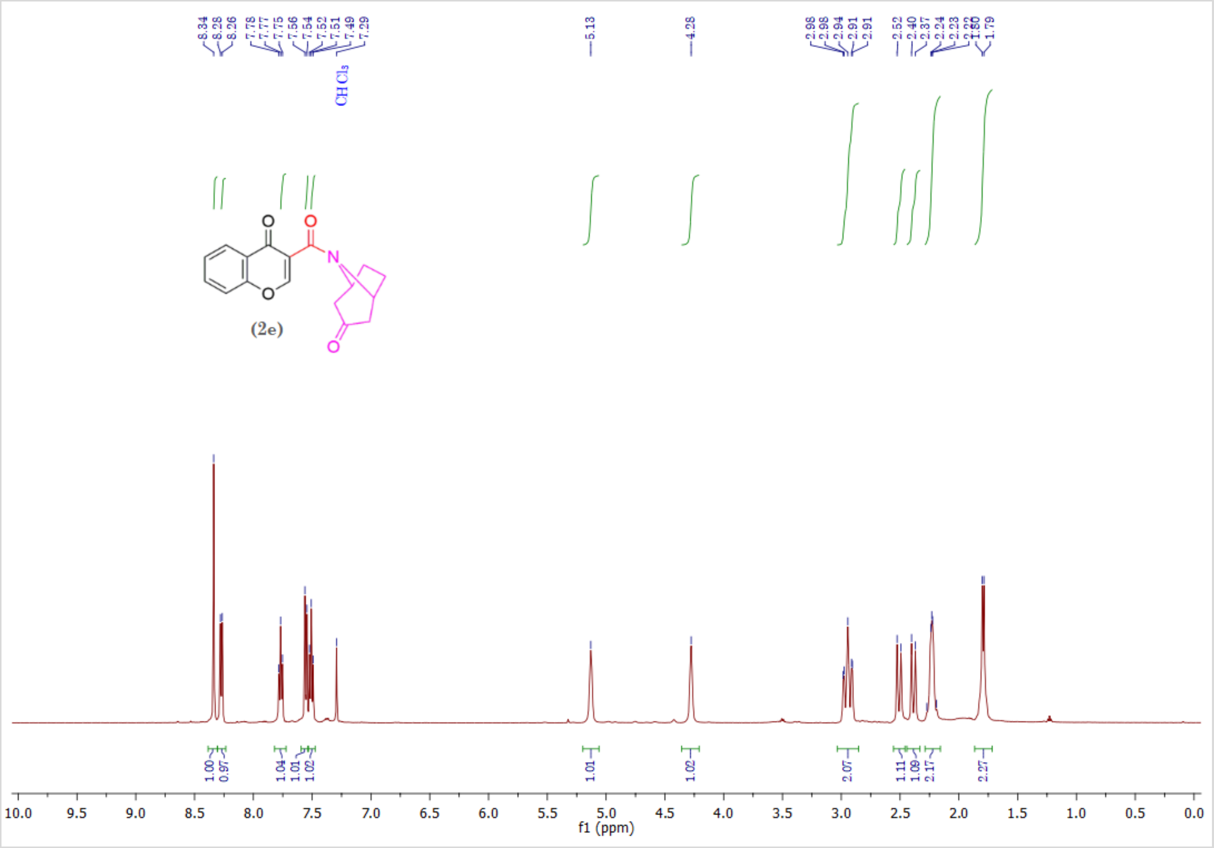

Figure S14. ^{1}H NMR spectrum of compound **2e** (500 MHz, $CDCl_3$)

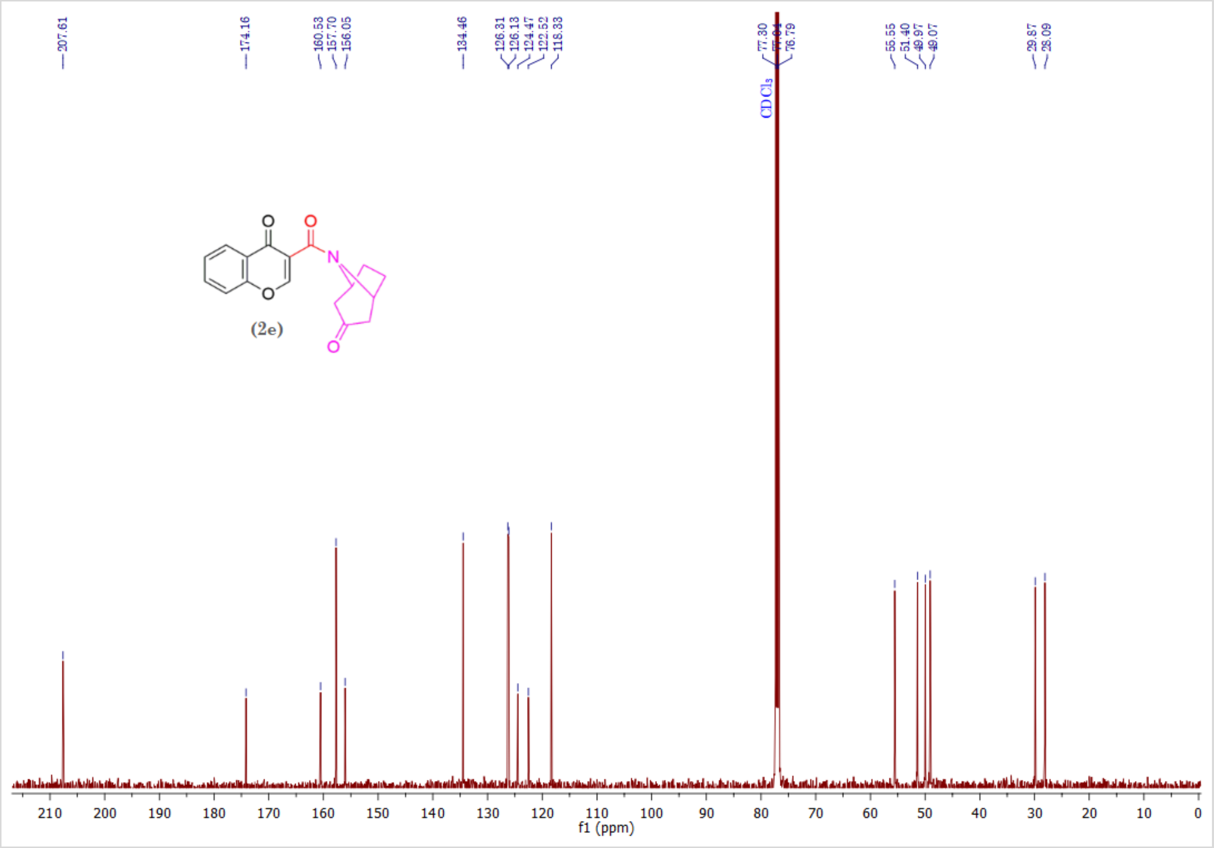

Figure S15. $^{13}C\{^{1}H\}$ NMR spectrum of compound **2e** (125 MHz, $CDCl_3$)

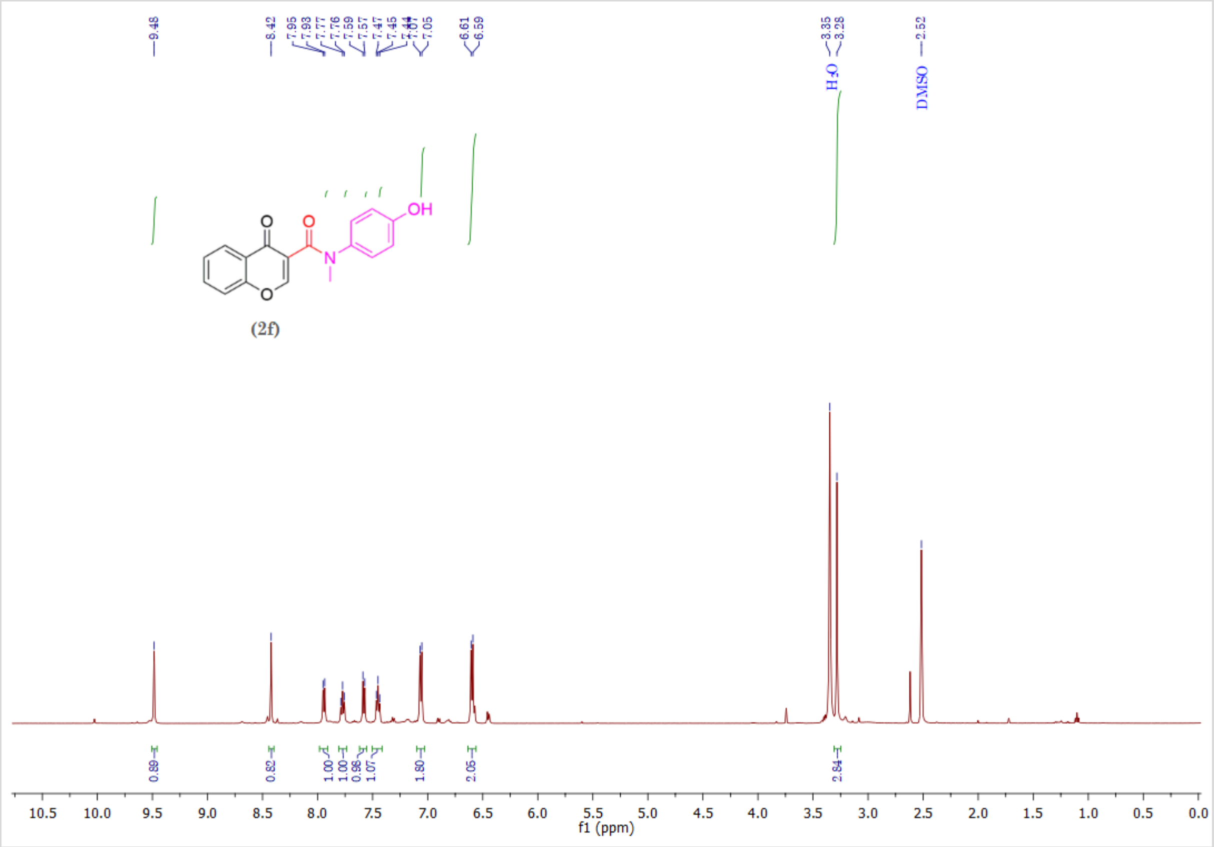

Figure S16. ^{1}H NMR spectrum of compound **2f** (500 MHz, DMSO-d$_6$)

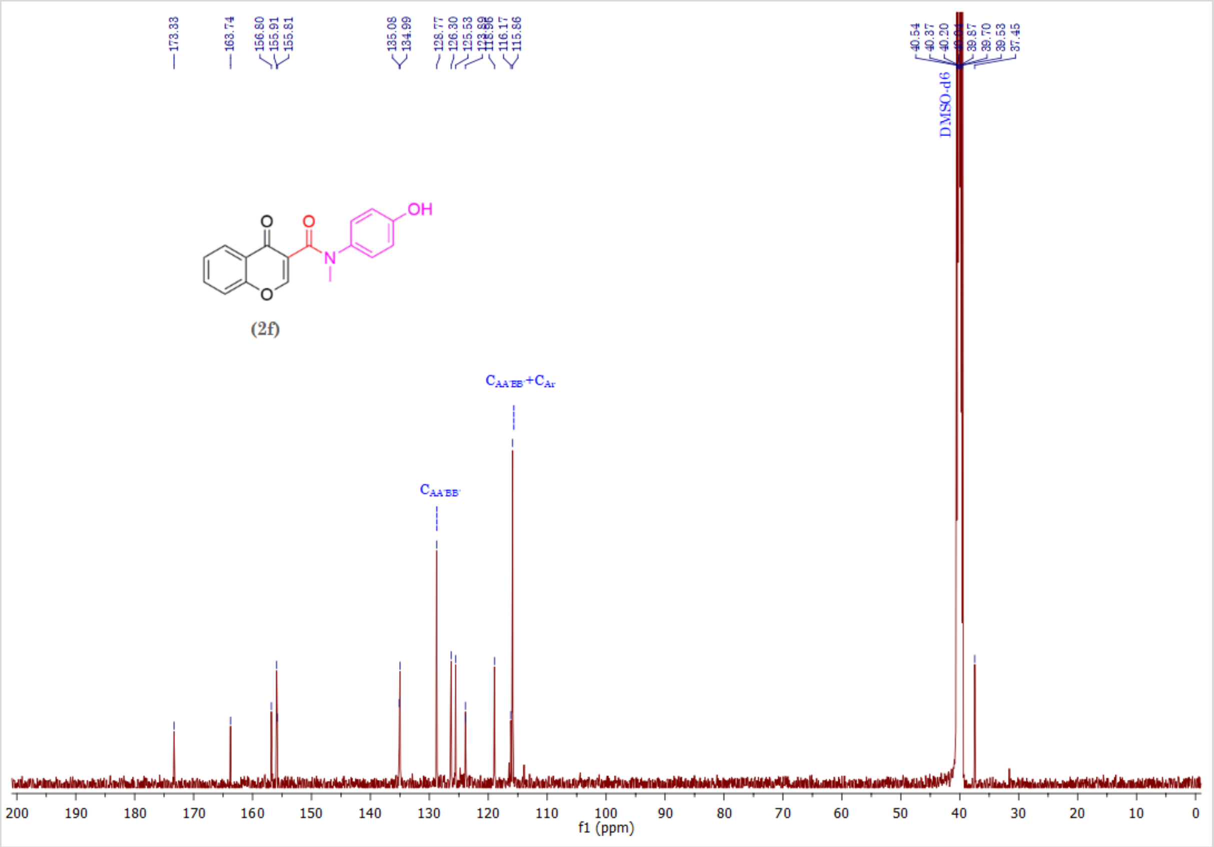

Figure S17. ^{13}C{^{1}H} NMR spectrum of compound **2f** (125 MHz, DMSO-d$_6$)

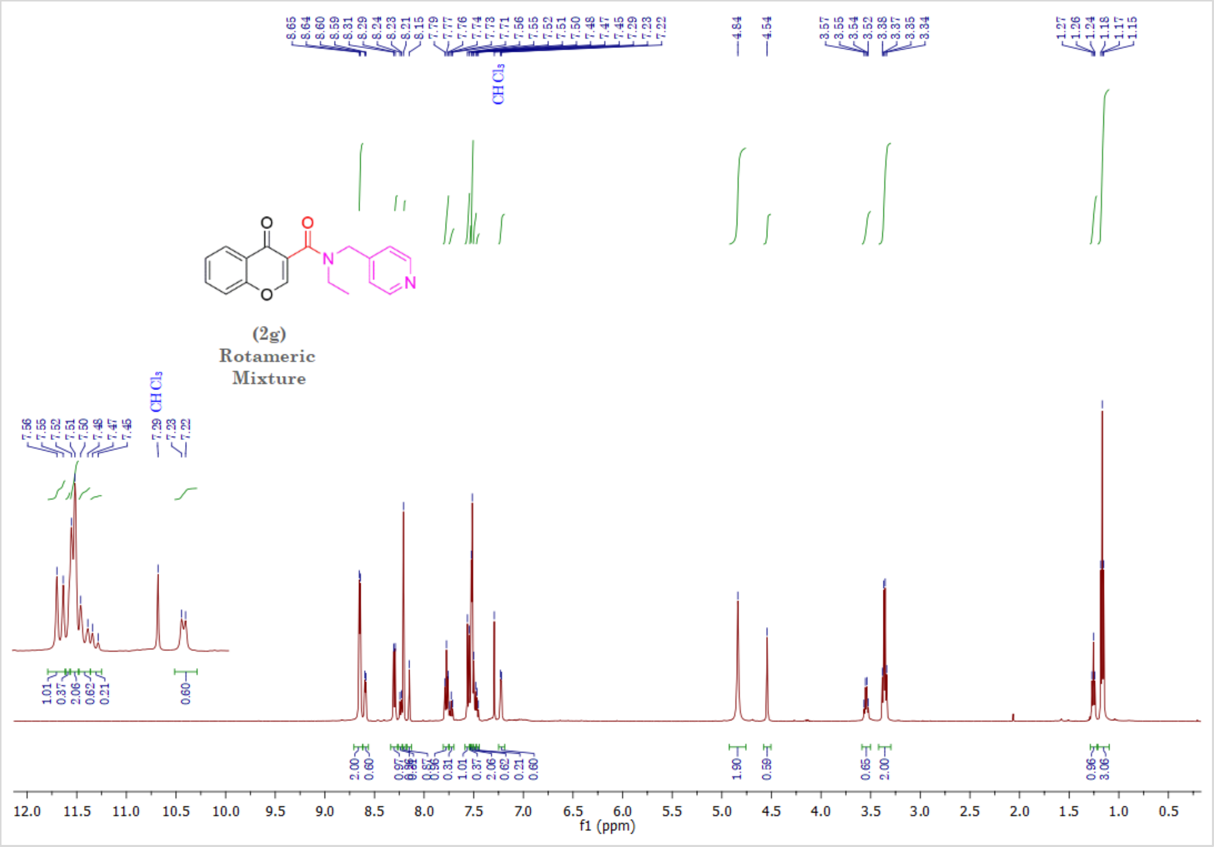

Figure S18. ^{1}H NMR spectrum of compound **2g** (500 MHz, $CDCl_3$)

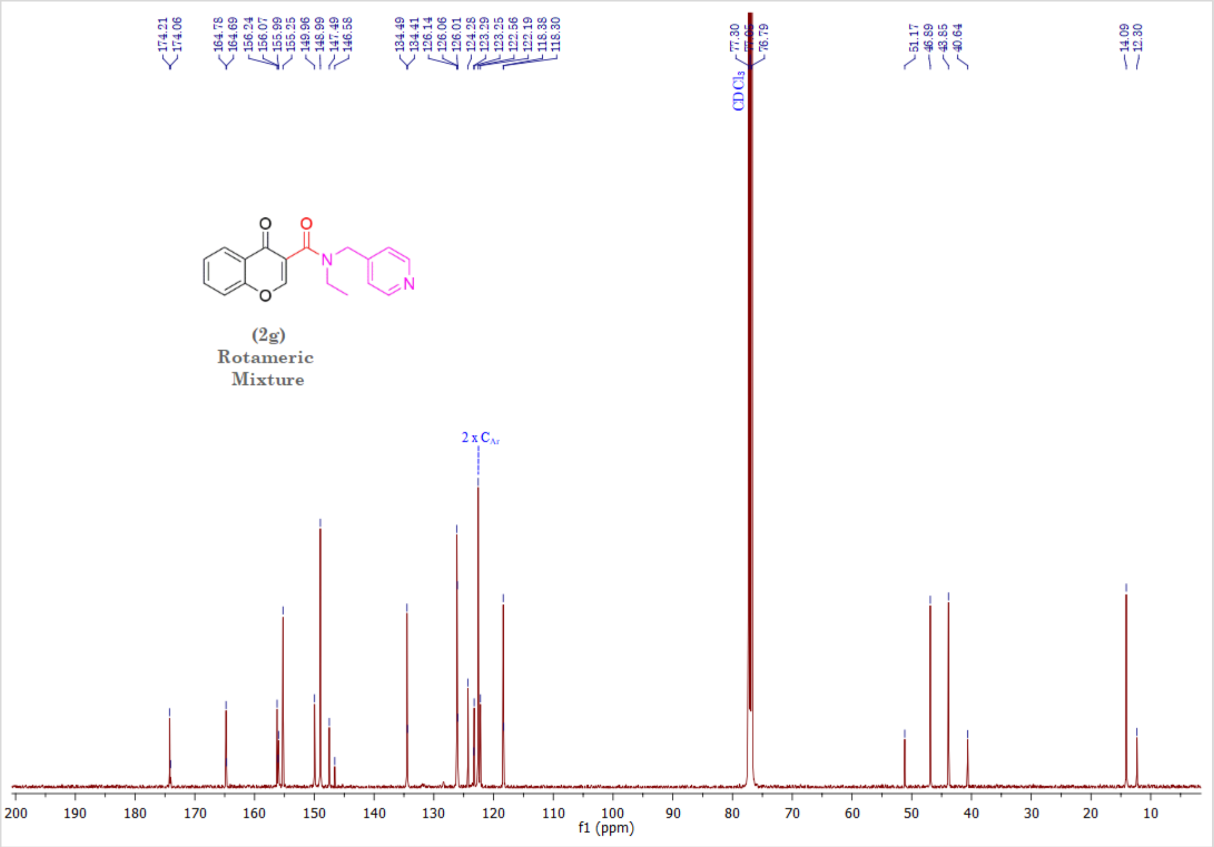

Figure S19. $^{13}C\{^{1}H\}$ NMR spectrum of compound **2g** (125 MHz, $CDCl_3$)

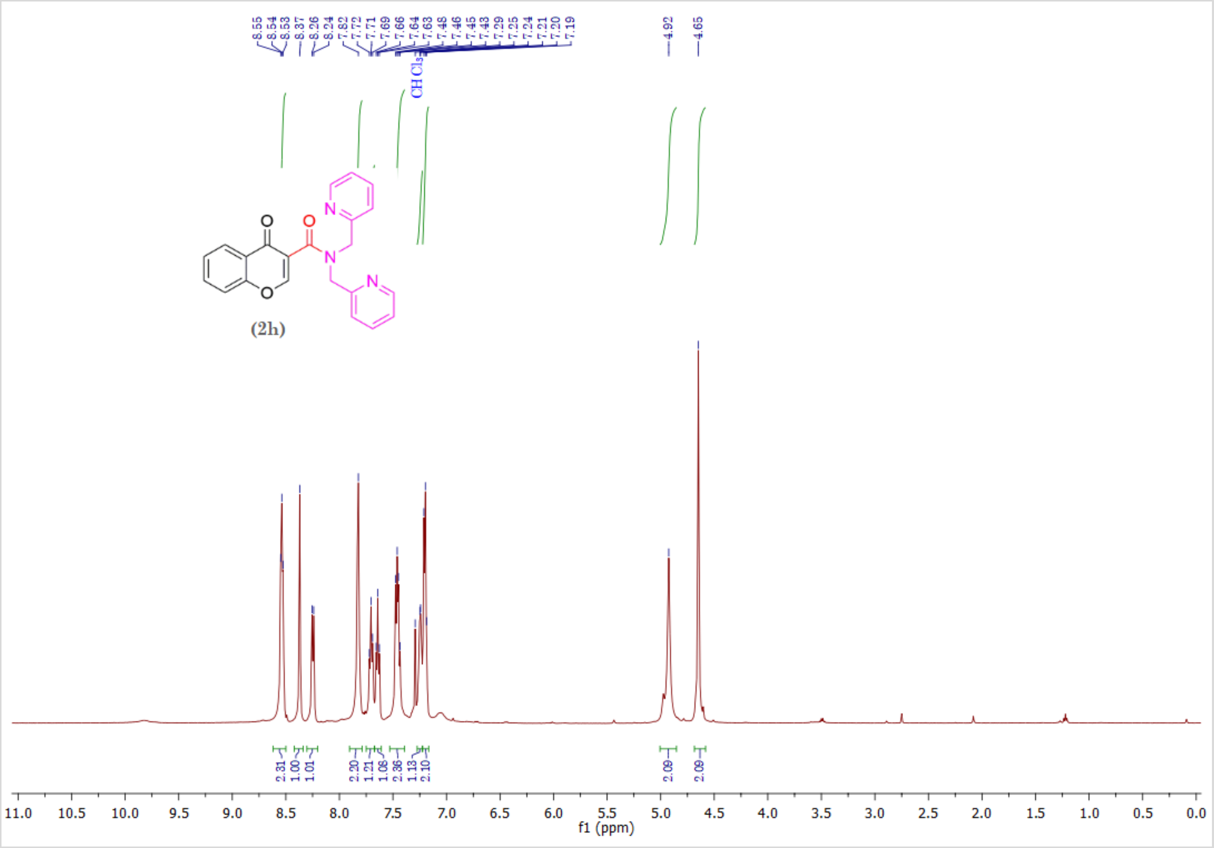

Figure S20. ^{1}H NMR spectrum of compound **2h** (500 MHz, $CDCl_3$)

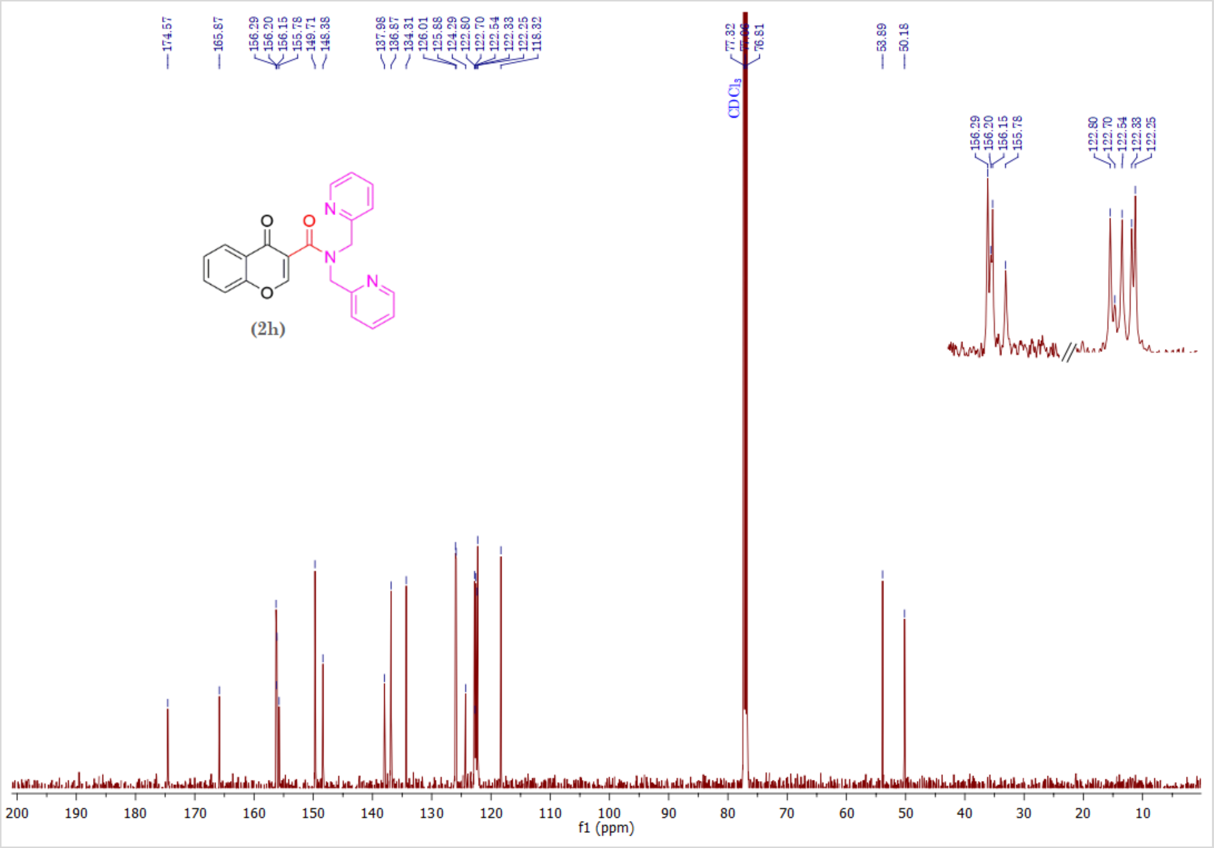

Figure S21. $^{13}C\{^{1}H\}$ NMR spectrum of compound **2h** (125 MHz, $CDCl_3$)

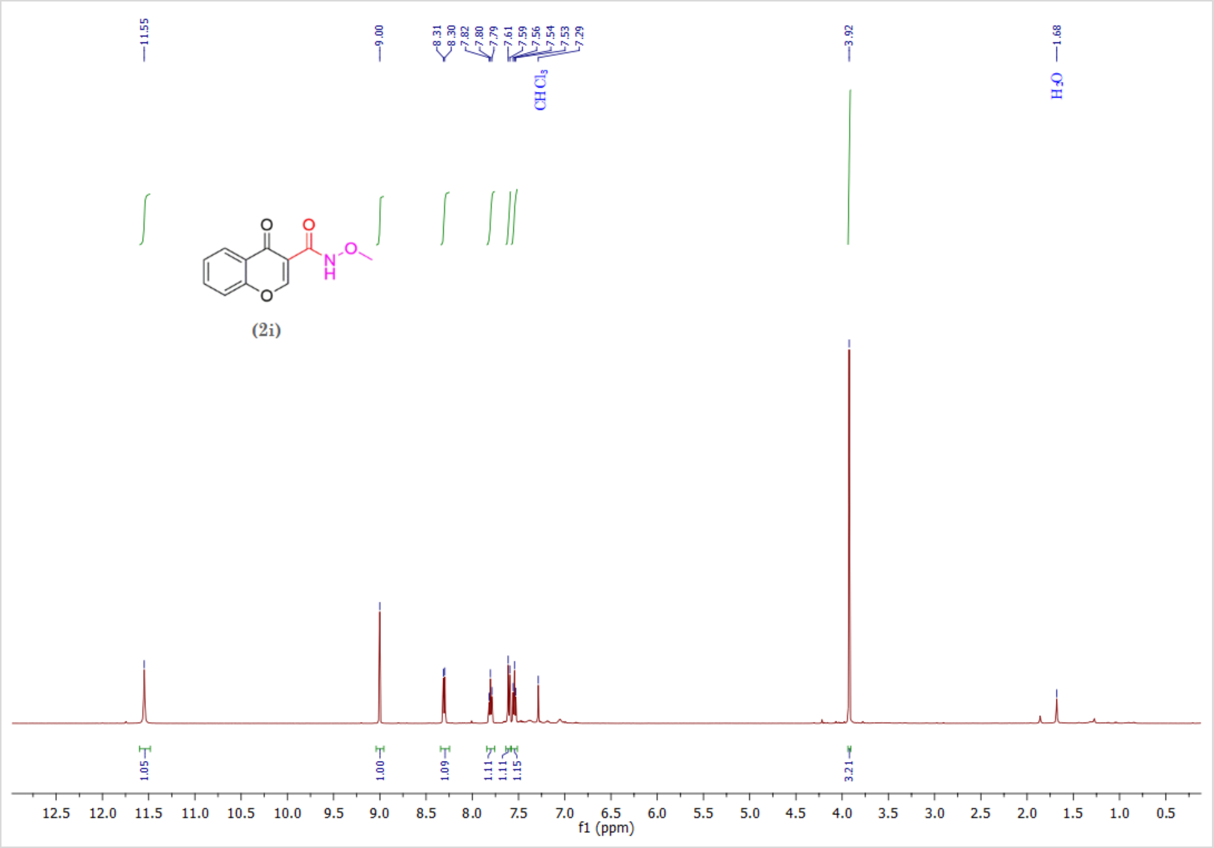

Figure S22. ^{1}H NMR spectrum of compound **2i** (500 MHz, $CDCl_3$)

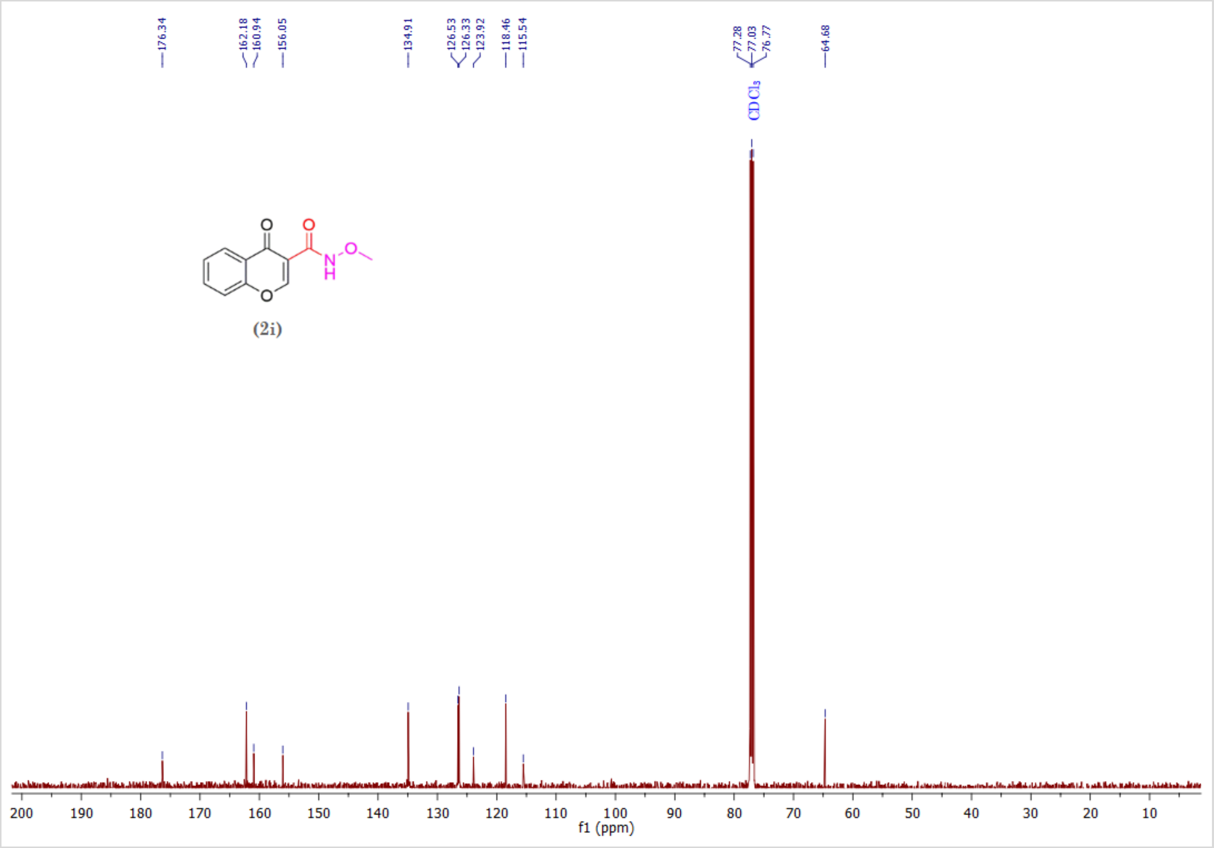

Figure S23. $^{13}C\{^{1}H\}$ NMR spectrum of compound **2i** (125 MHz, $CDCl_3$)

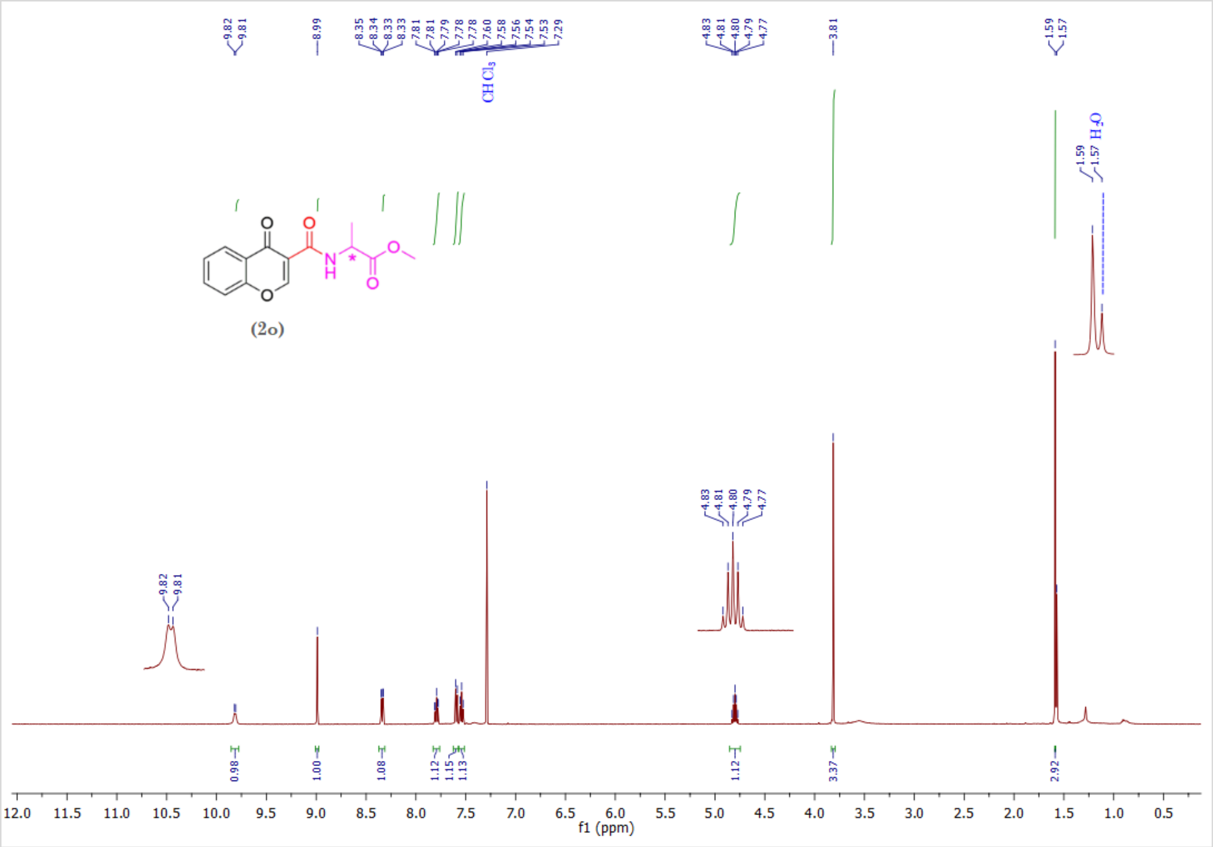

Figure S24. ${}^{1}H$ NMR spectrum of compound **2o** (500 MHz, $CDCl_3$)

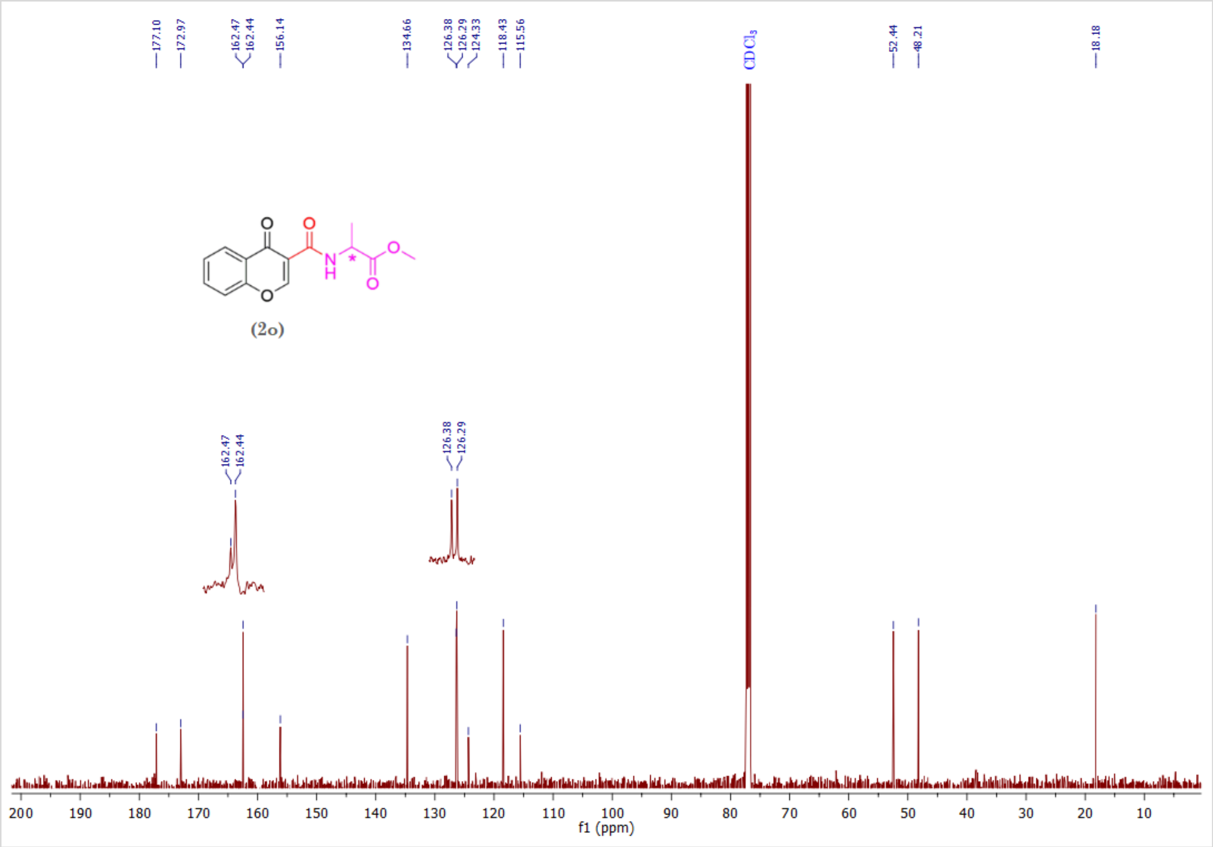

Figure S25. ${}^{13}C\{{}^{1}H\}$ NMR spectrum of compound **2o** (125 MHz, $CDCl_3$)

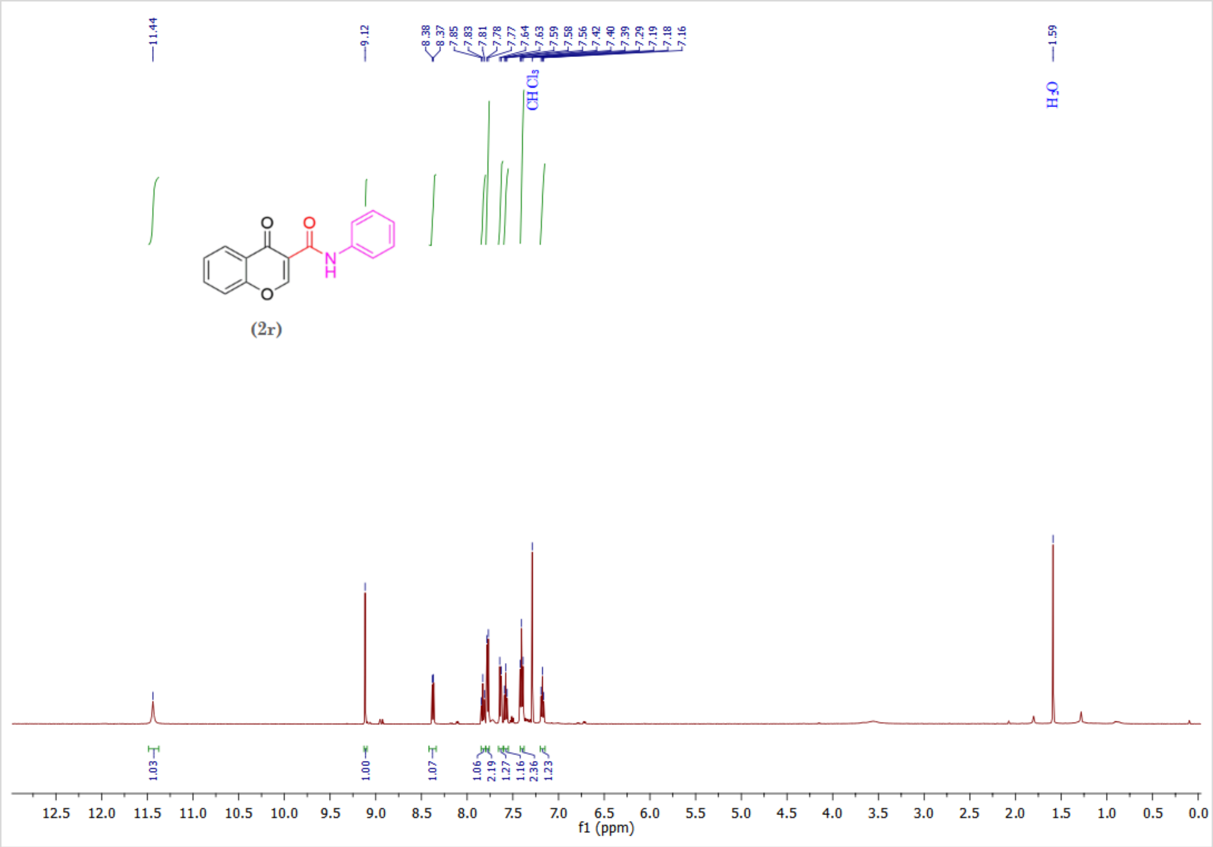

Figure S26. ^{1}H NMR spectrum of compound **2r** (500 MHz, $CDCl_3$)

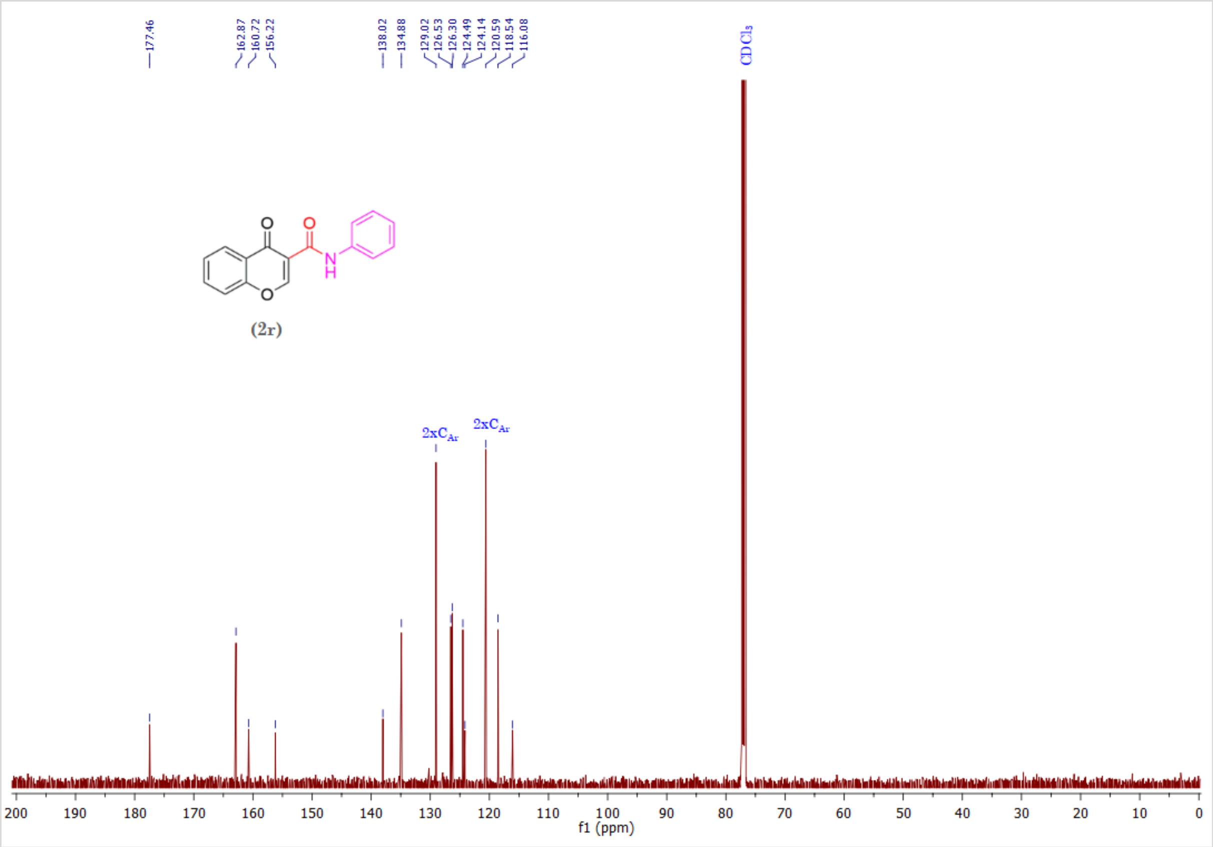

Figure S27. $^{13}C\{^{1}H\}$ NMR spectrum of compound **2r** (125 MHz, $CDCl_3$)

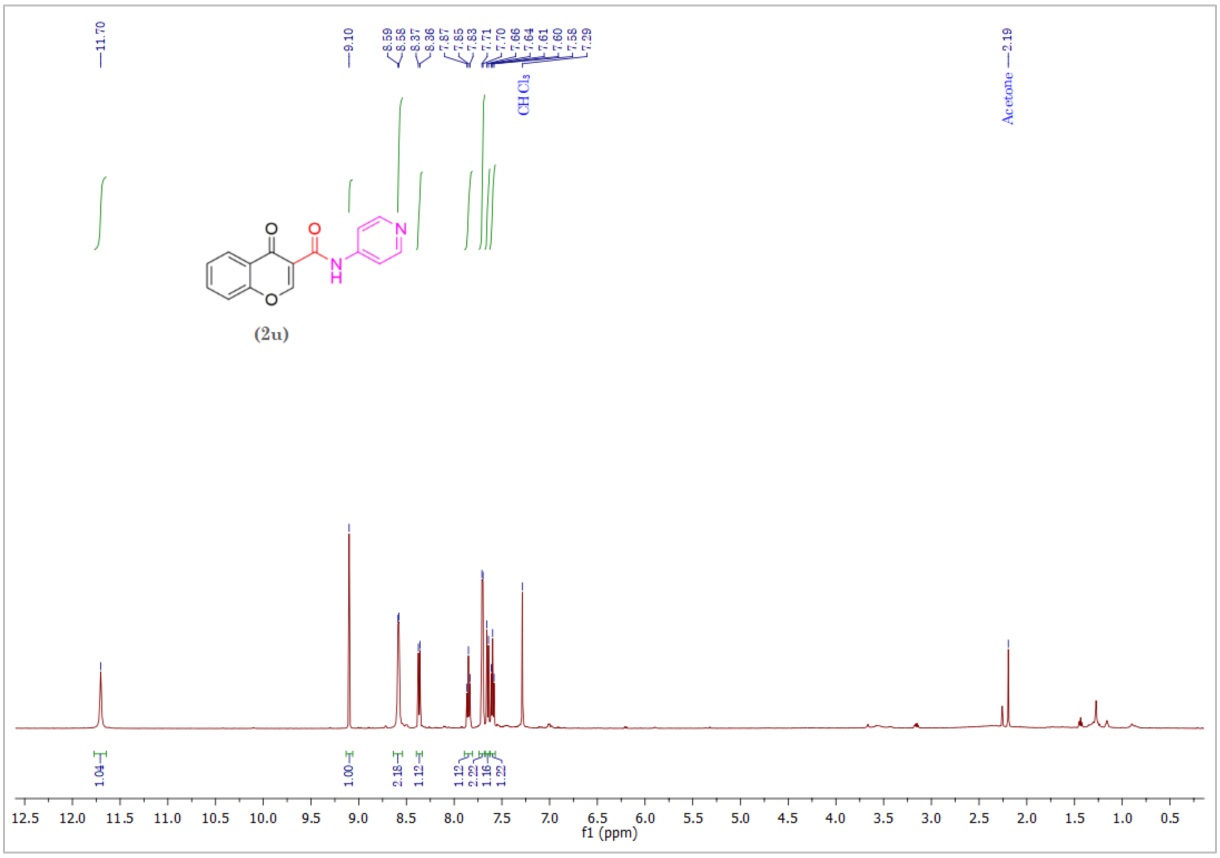

Figure S28. ^{1}H NMR spectrum of compound **2u** (500 MHz, $CDCl_3$)

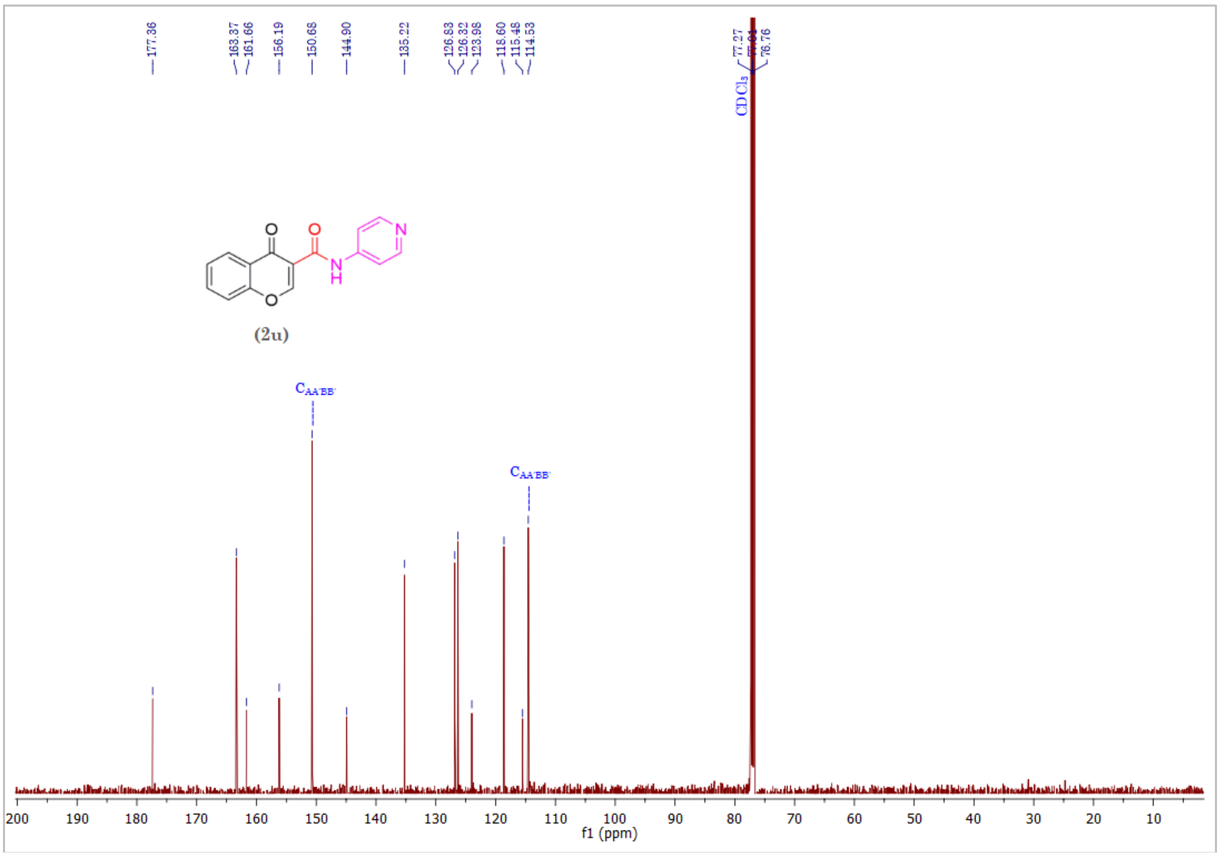

Figure S29. $^{13}C\{^1H\}$ NMR spectrum of compound **2u** (125 MHz, $CDCl_3$)

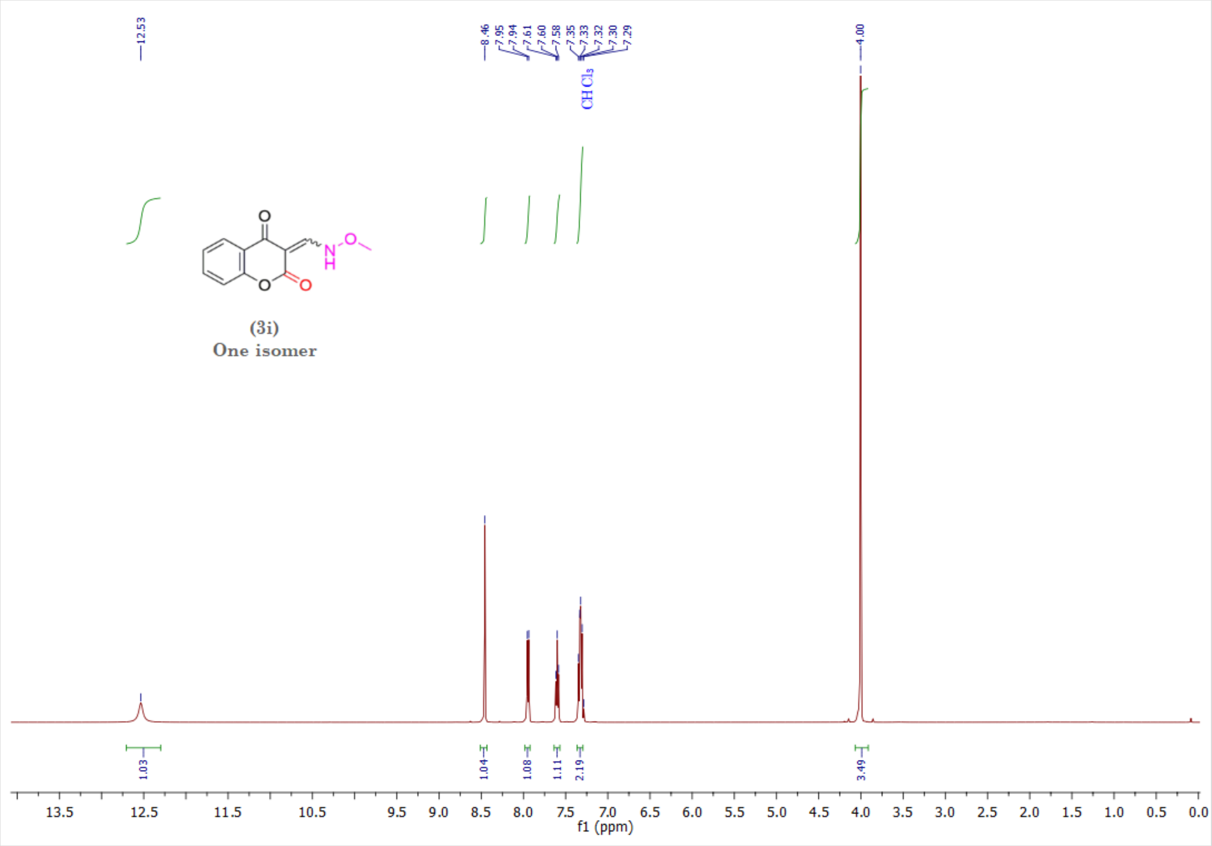

Figure S30. ^{1}H NMR spectrum of compound **3i** (500 MHz, $CDCl_3$)

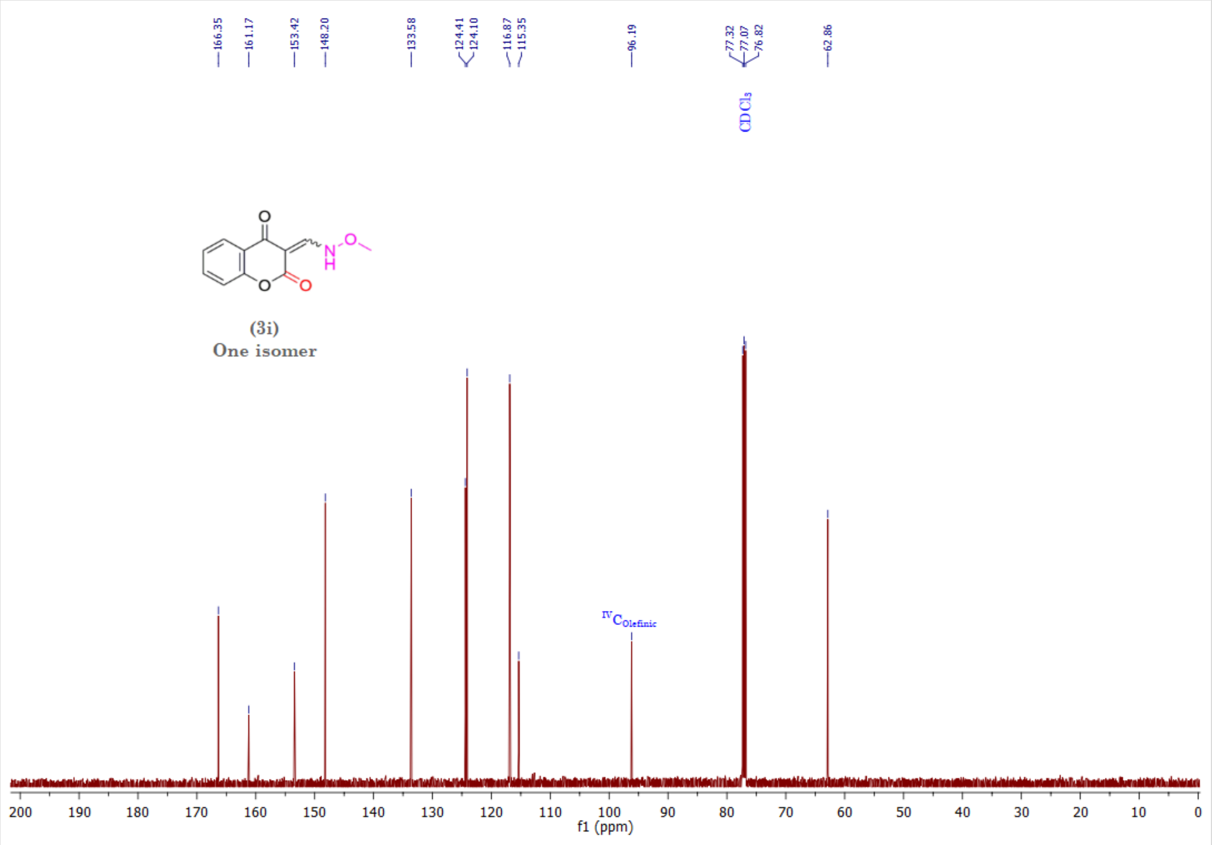

Figure S31. $^{13}C\{^{1}H\}$ NMR spectrum of compound **3i** (125 MHz, $CDCl_3$)

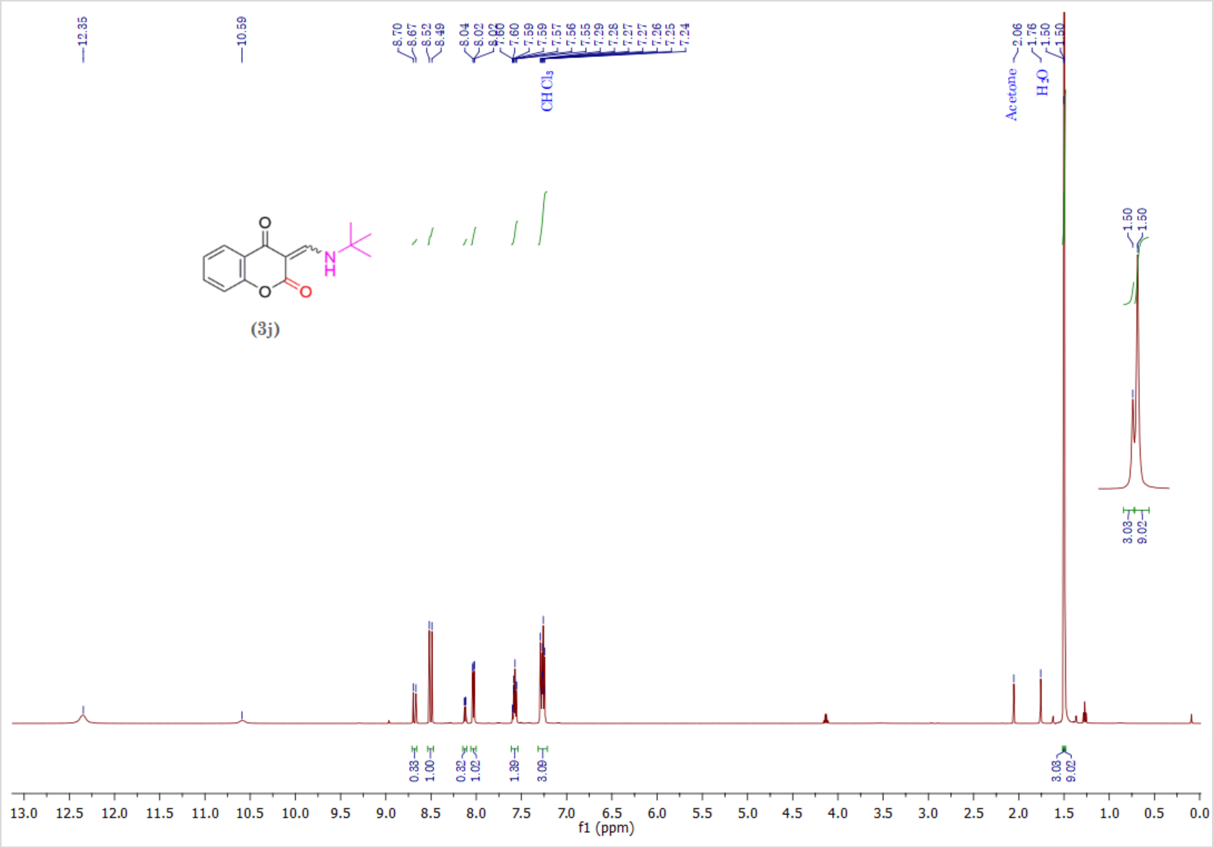

Figure S32. ^{1}H NMR spectrum of compound **3j** (500 MHz, $CDCl_3$)

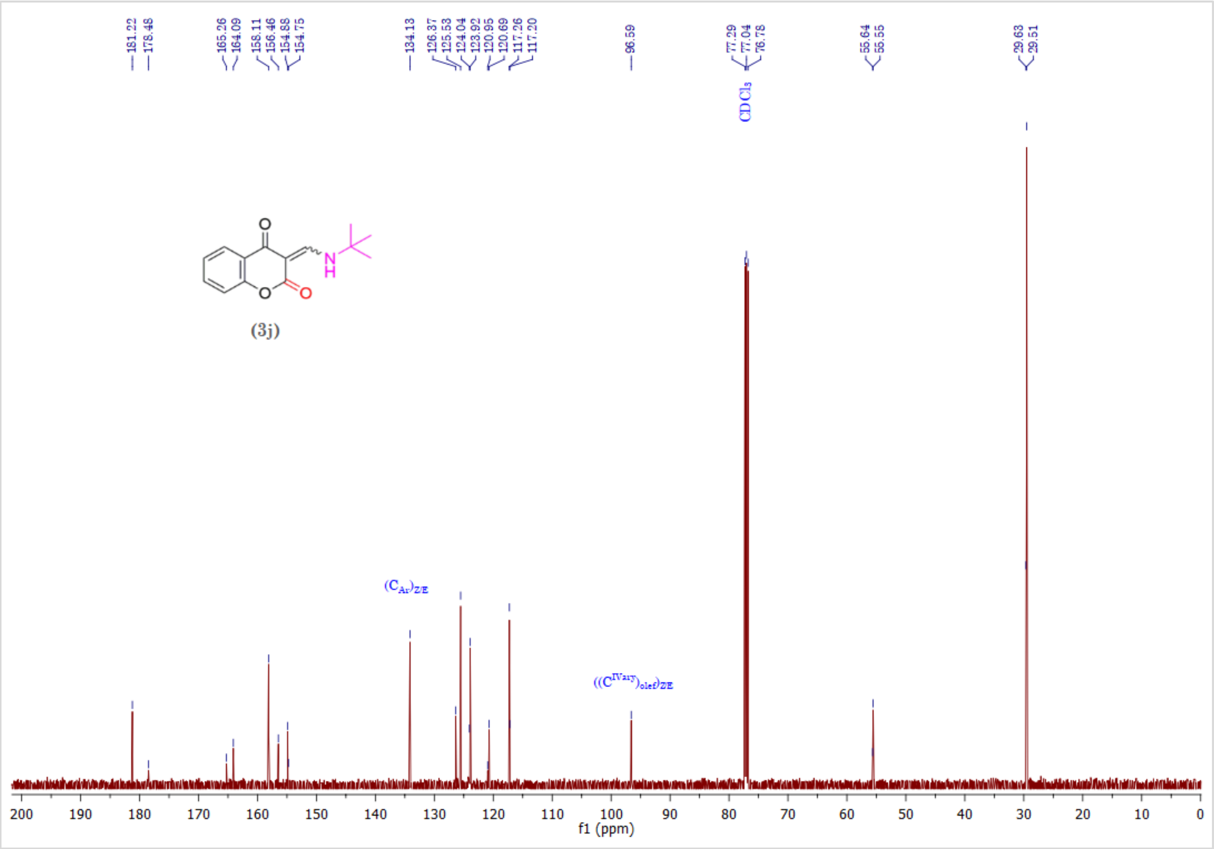

Figure S33. $^{13}C\{^{1}H\}$ NMR spectrum of compound **3j** (125 MHz, $CDCl_3$)

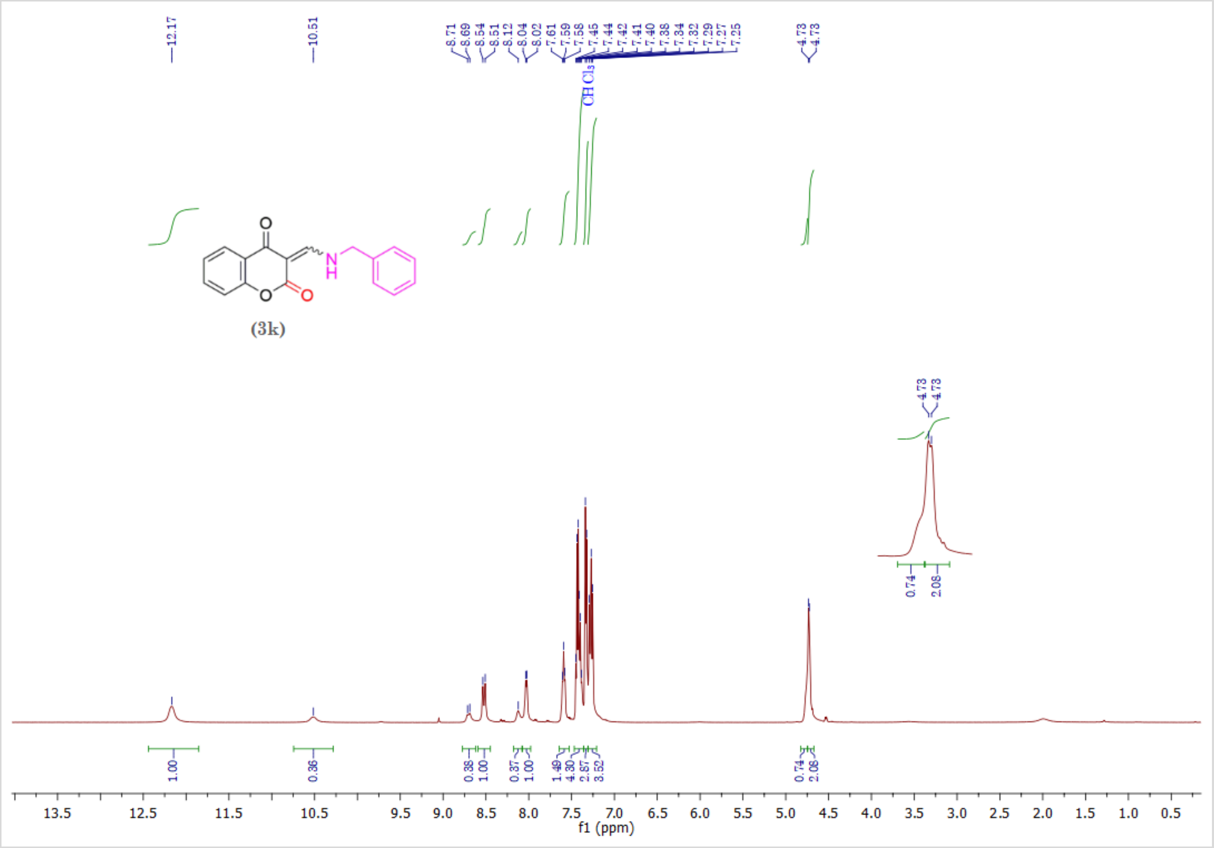

Figure S34. ^{1}H NMR spectrum of compound **3k** (500 MHz, $CDCl_3$)

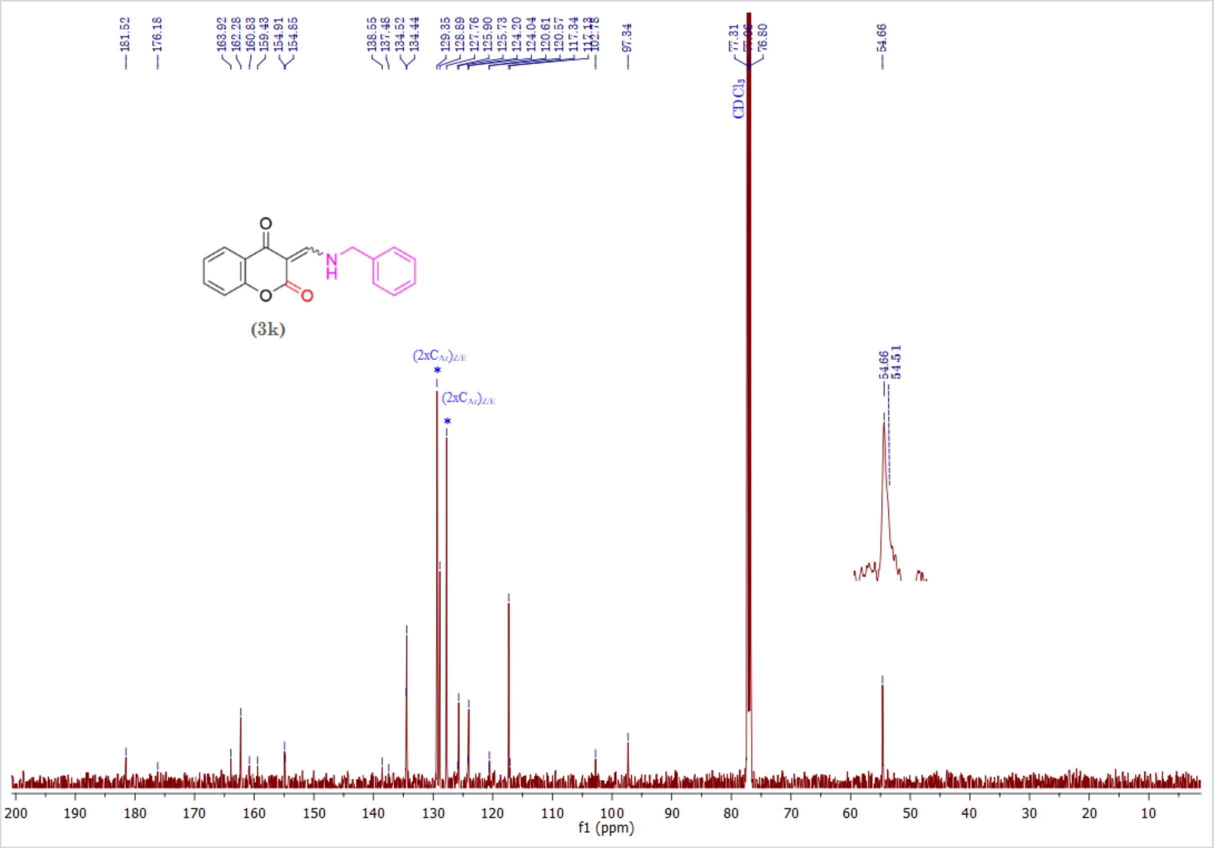

Figure S35. $^{13}C\{^{1}H\}$ NMR spectrum of compound **3k** (125 MHz, $CDCl_3$)

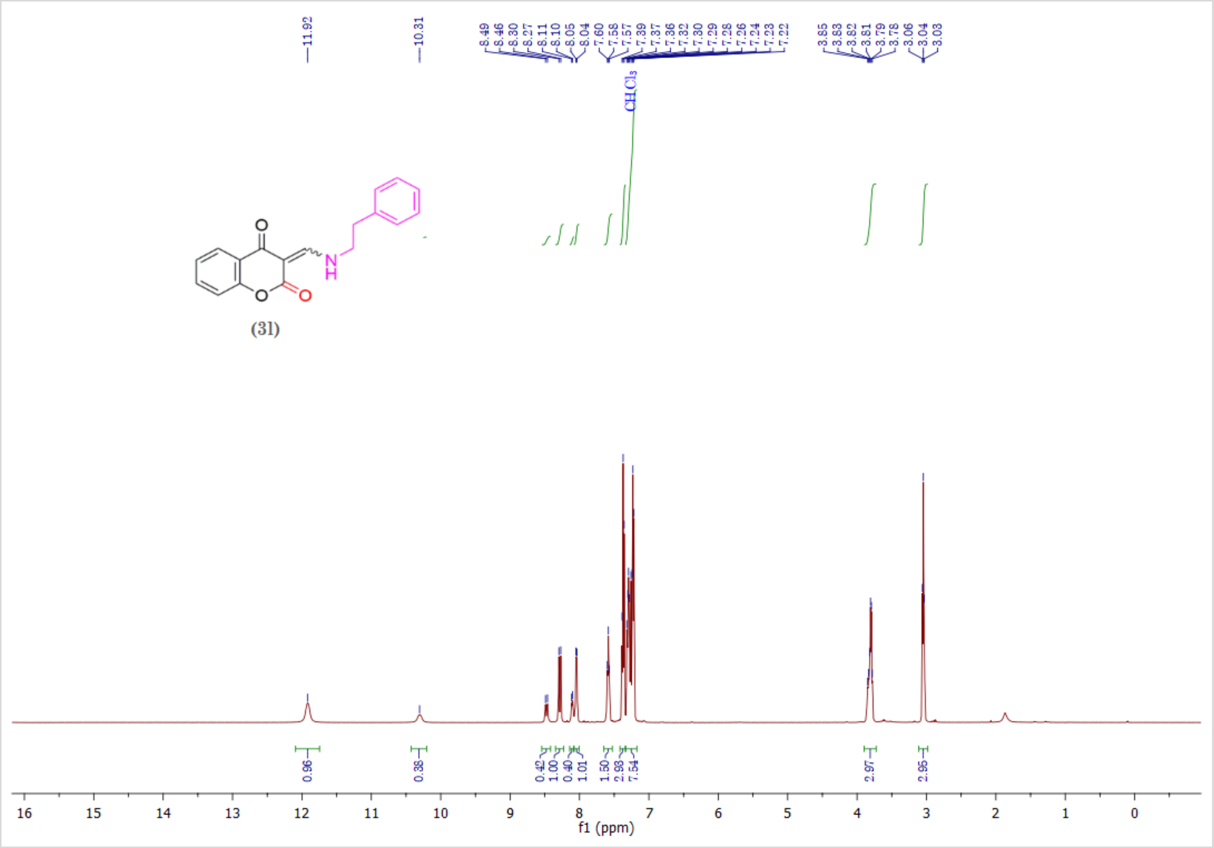

Figure S36. ^{1}H NMR spectrum of compound **3l** (500 MHz, $CDCl_3$)

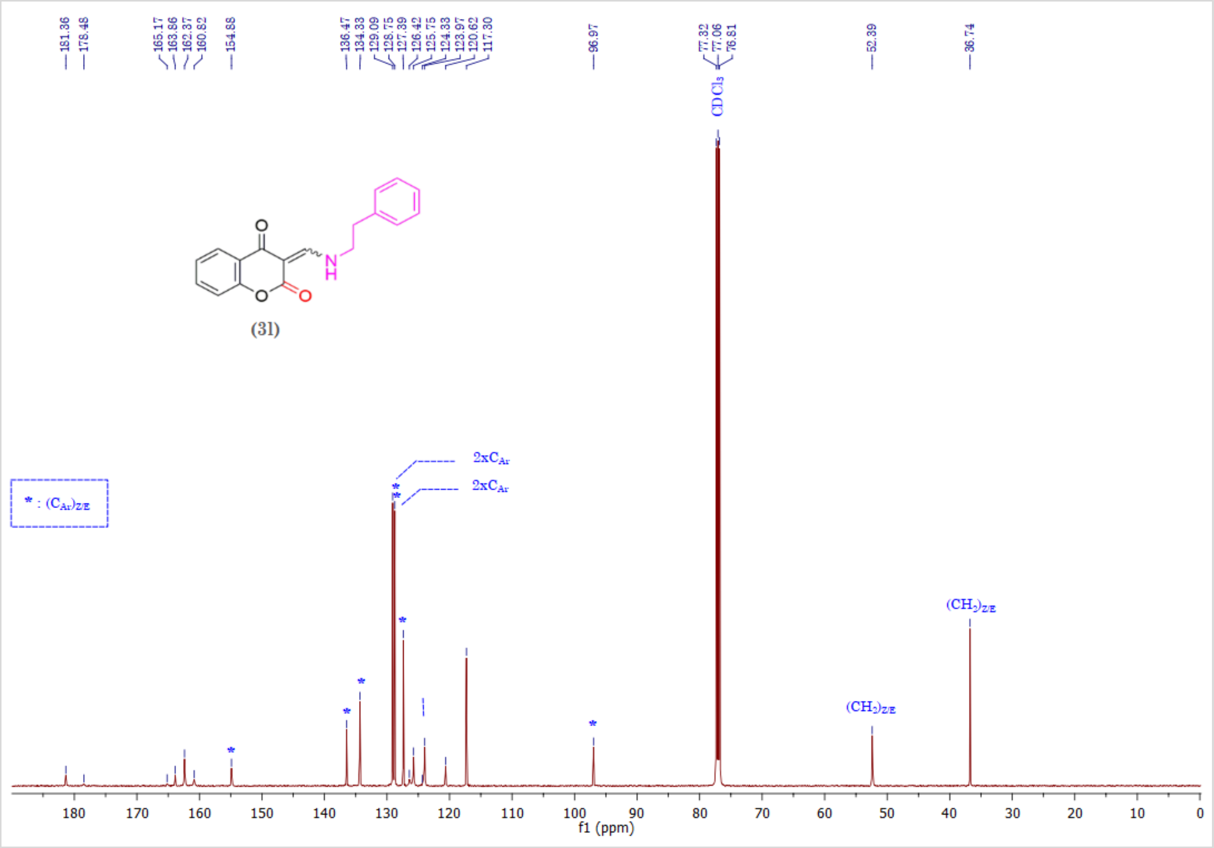

Figure S37. $^{13}C\{^{1}H\}$ NMR spectrum of compound **3l** (125 MHz, $CDCl_3$)

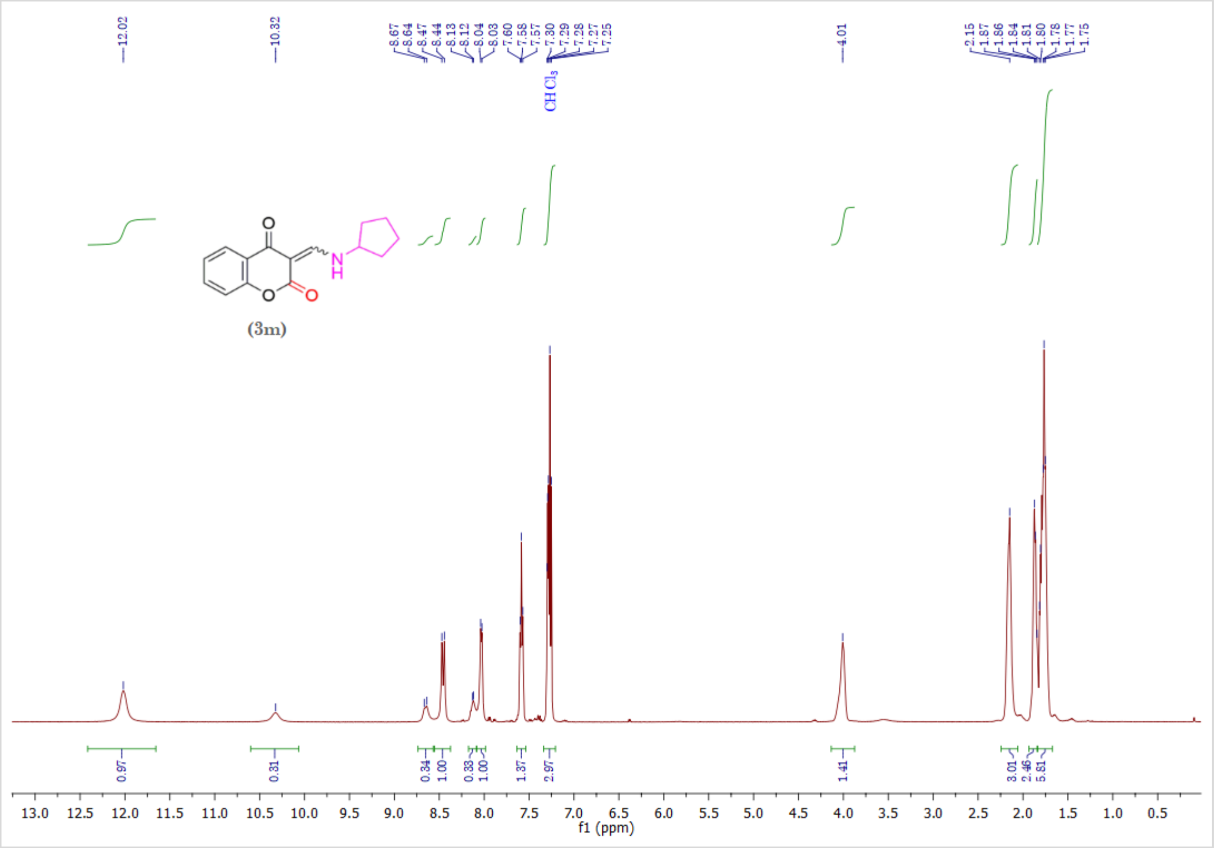

Figure S38. ^{1}H NMR spectrum of compound **3m** (500 MHz, $CDCl_3$)

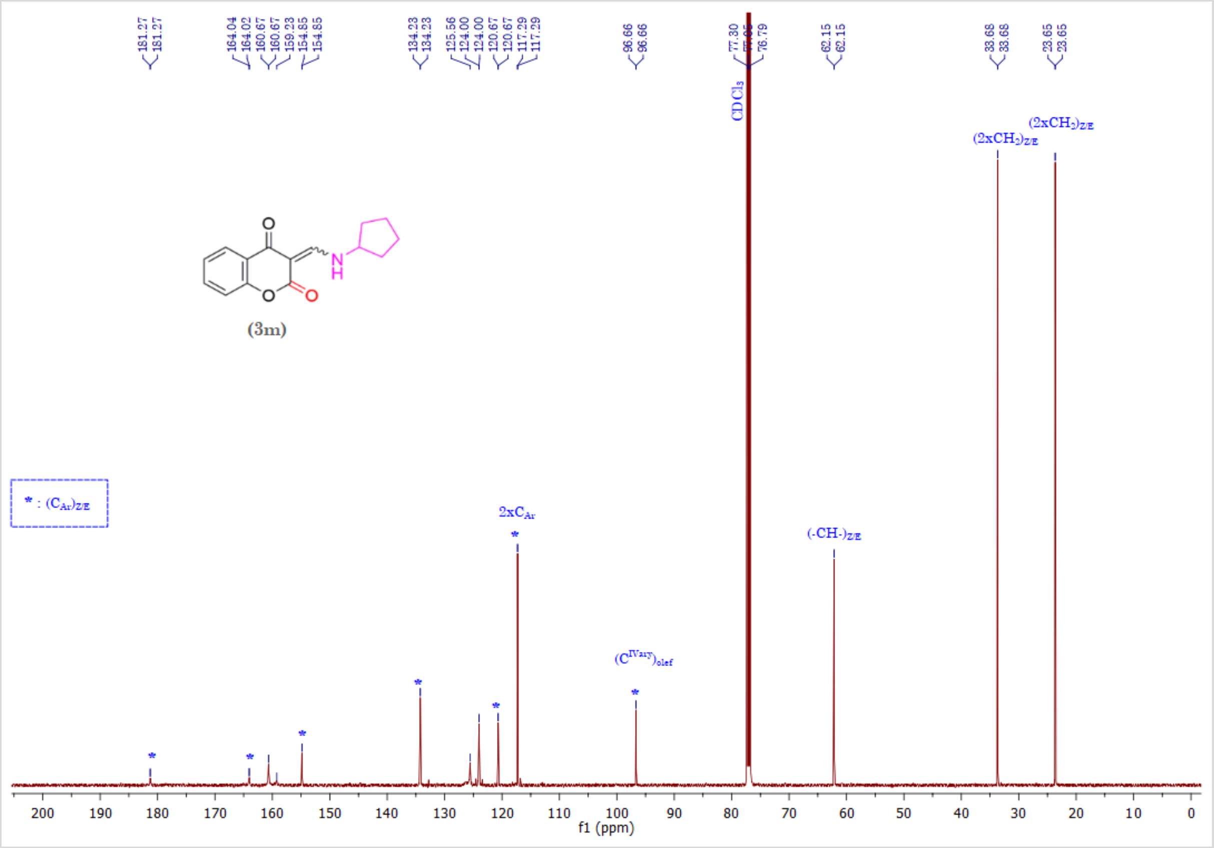

Figure S39. $^{13}C\{^{1}H\}$ NMR spectrum of compound **3m** (125 MHz, $CDCl_3$)

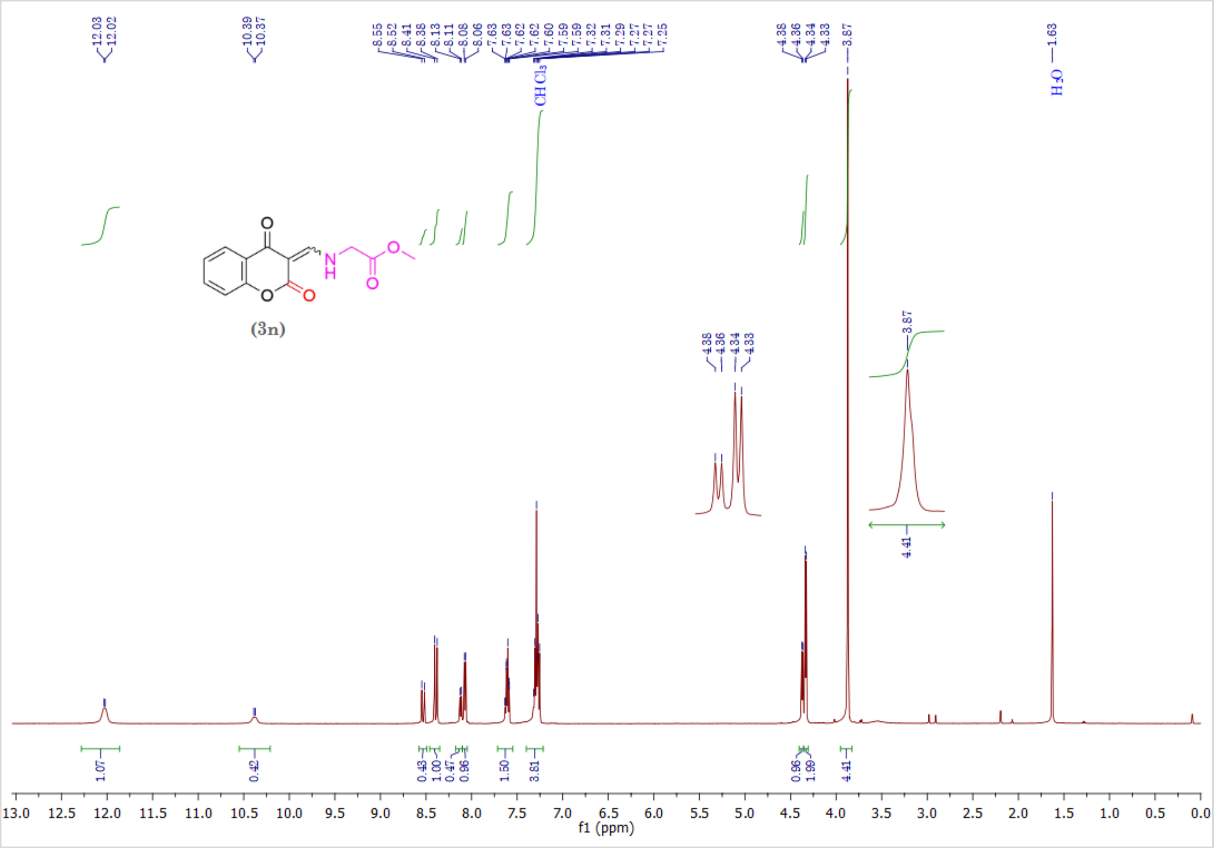

Figure S40. ^{1}H NMR spectrum of compound **3n** (500 MHz, $CDCl_3$)

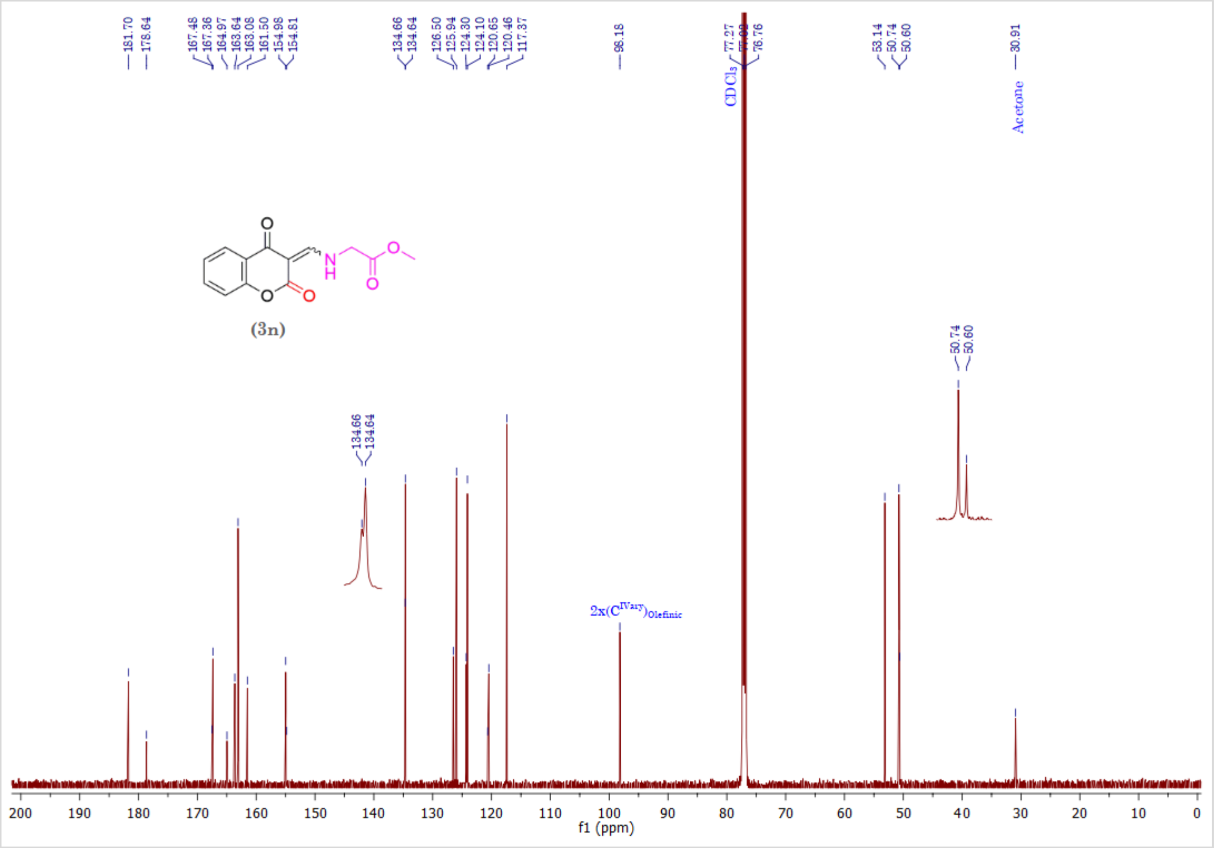

Figure S41. $^{13}C\{^{1}H\}$ NMR spectrum of compound **3n** (125 MHz, $CDCl_3$)

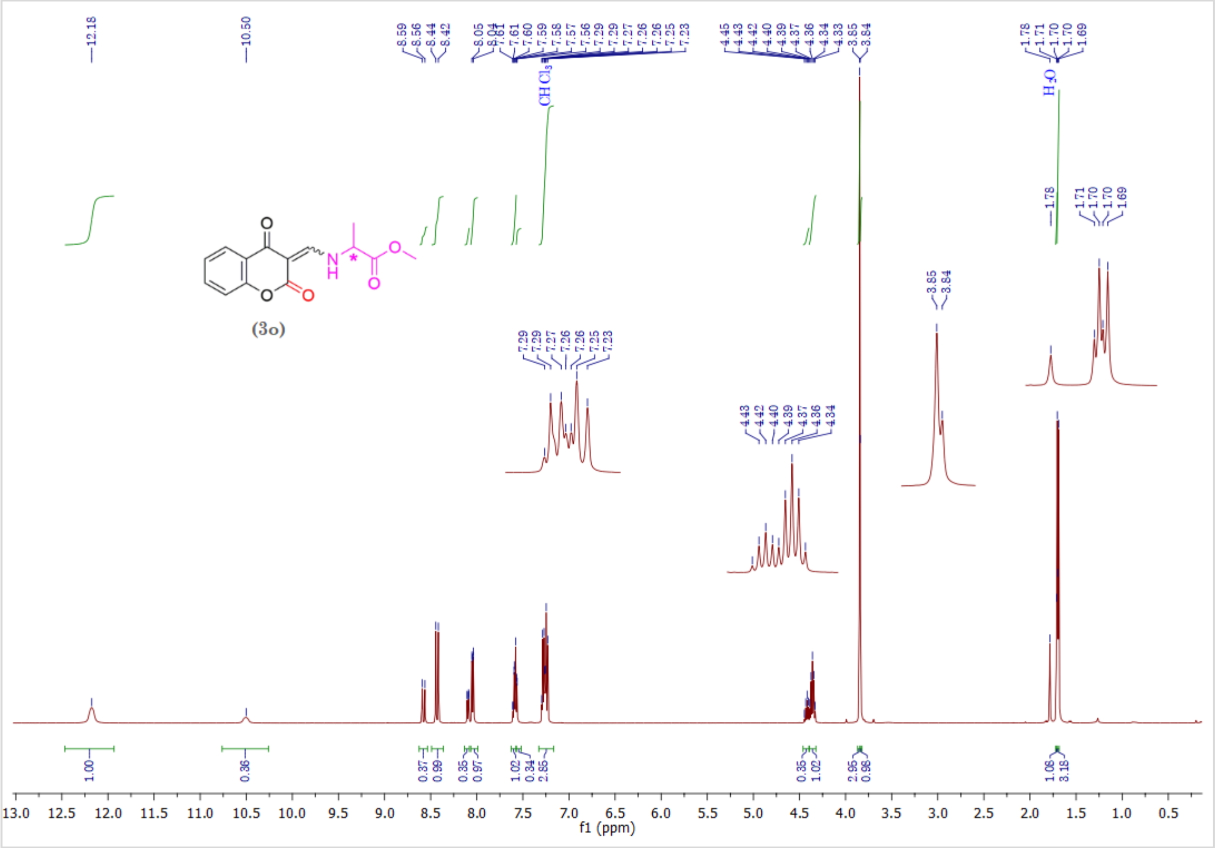

Figure S42. ^{1}H NMR spectrum of compound **3o** (500 MHz, $CDCl_3$)

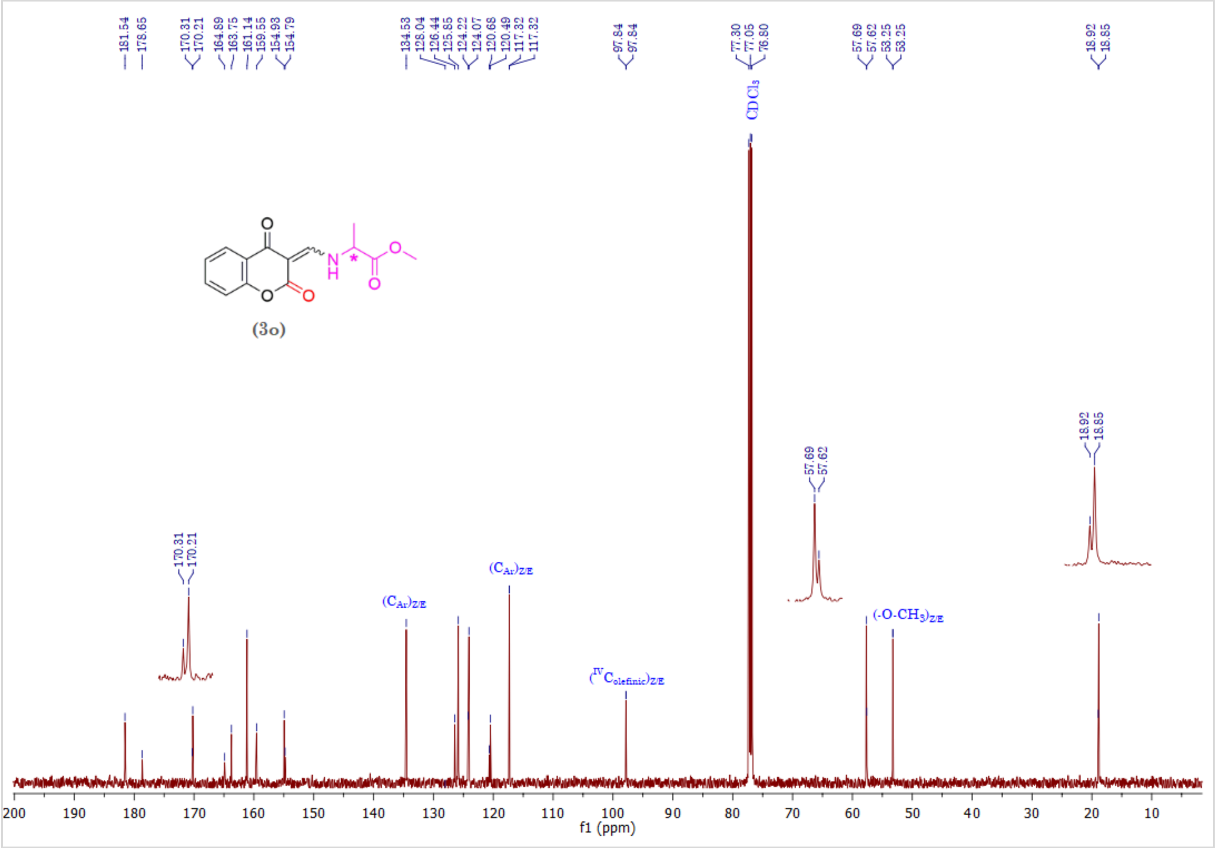

Figure S43. $^{13}C\{^{1}H\}$ NMR spectrum of compound **3o** (125 MHz, $CDCl_3$)

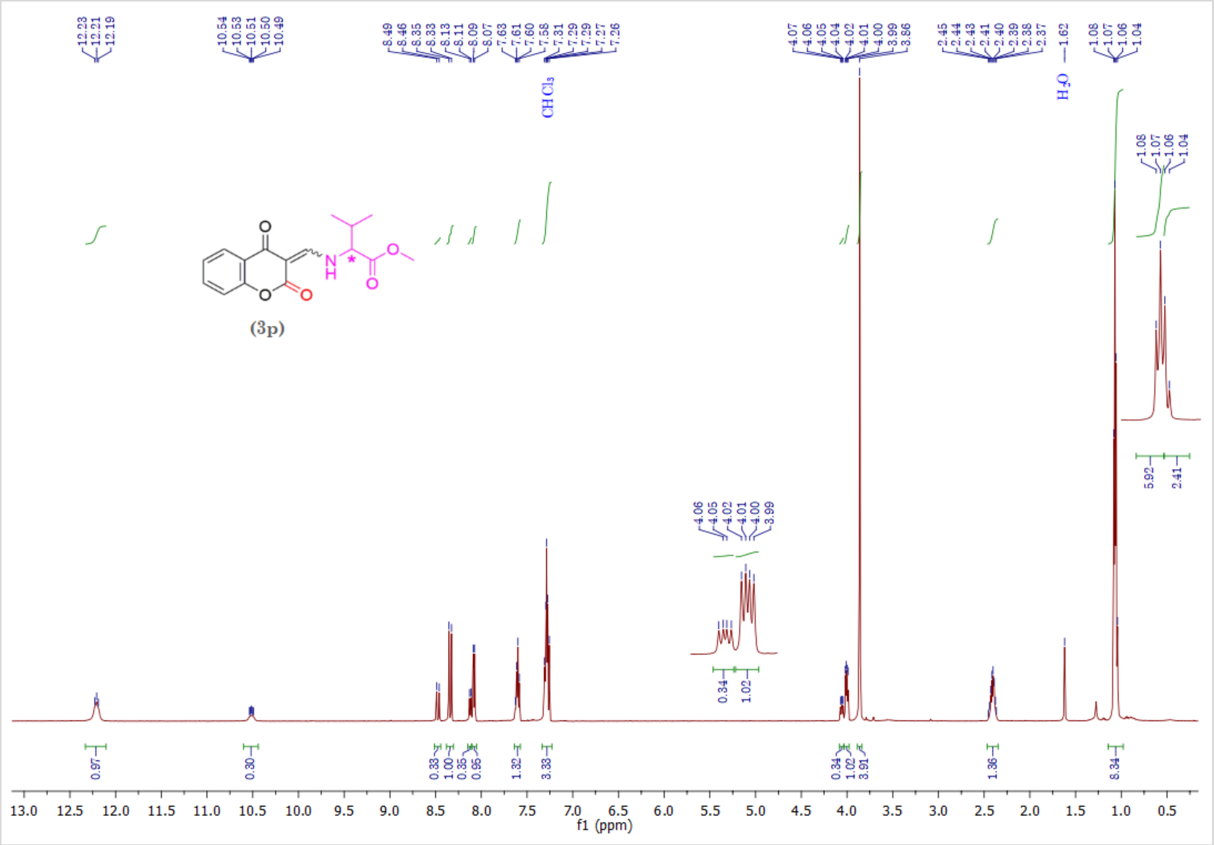

Figure S44. ^{1}H NMR spectrum of compound **3p** (500 MHz, $CDCl_3$)

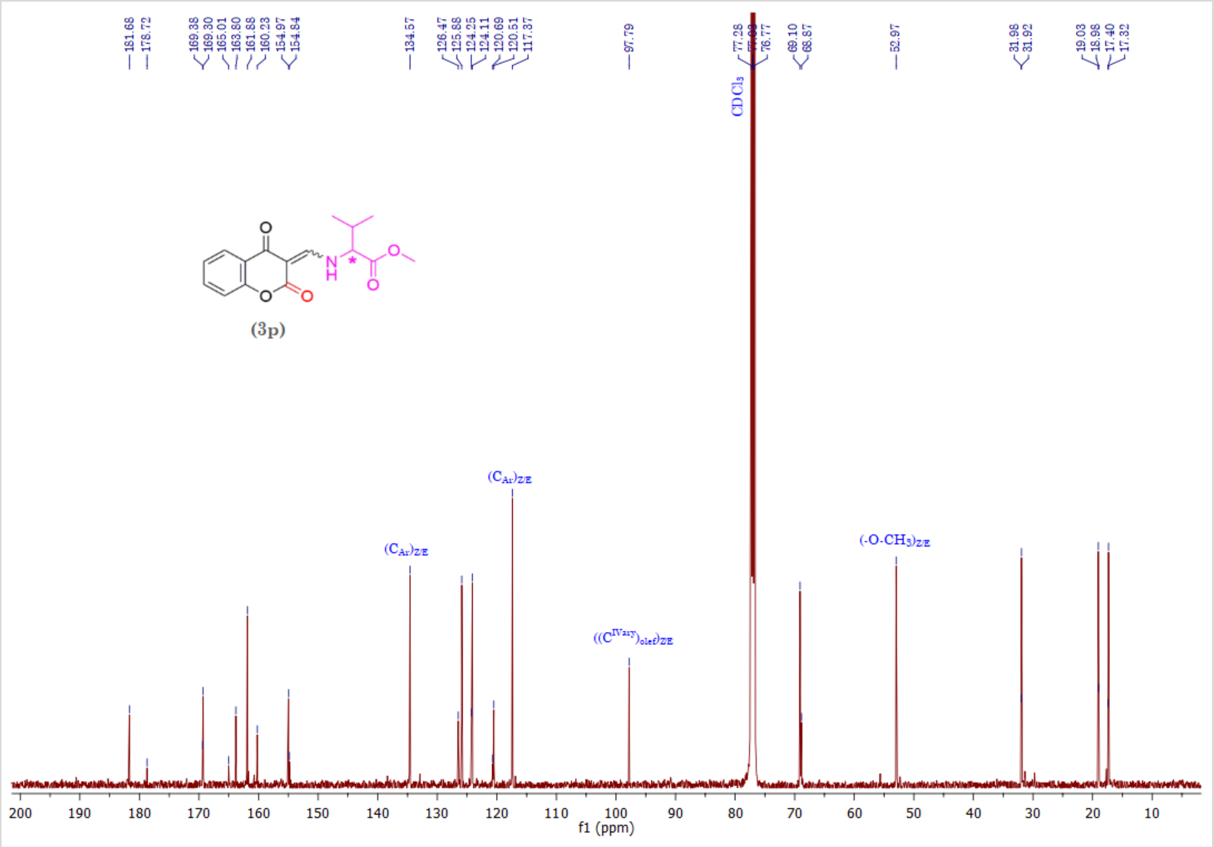

Figure S45. $^{13}C\{^{1}H\}$ NMR spectrum of compound **3p** (125 MHz, $CDCl_3$)

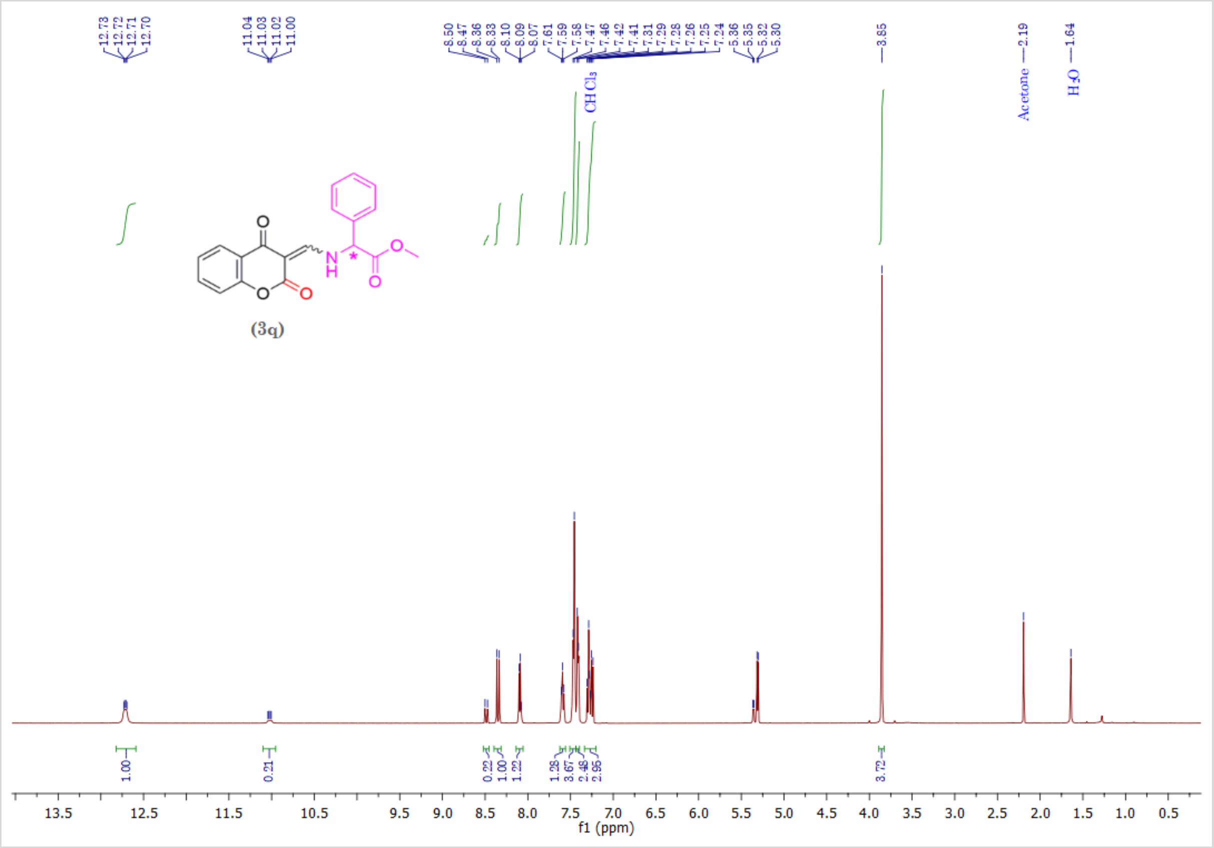

Figure S46. ^{1}H NMR spectrum of compound **3q** (500 MHz, $CDCl_3$)

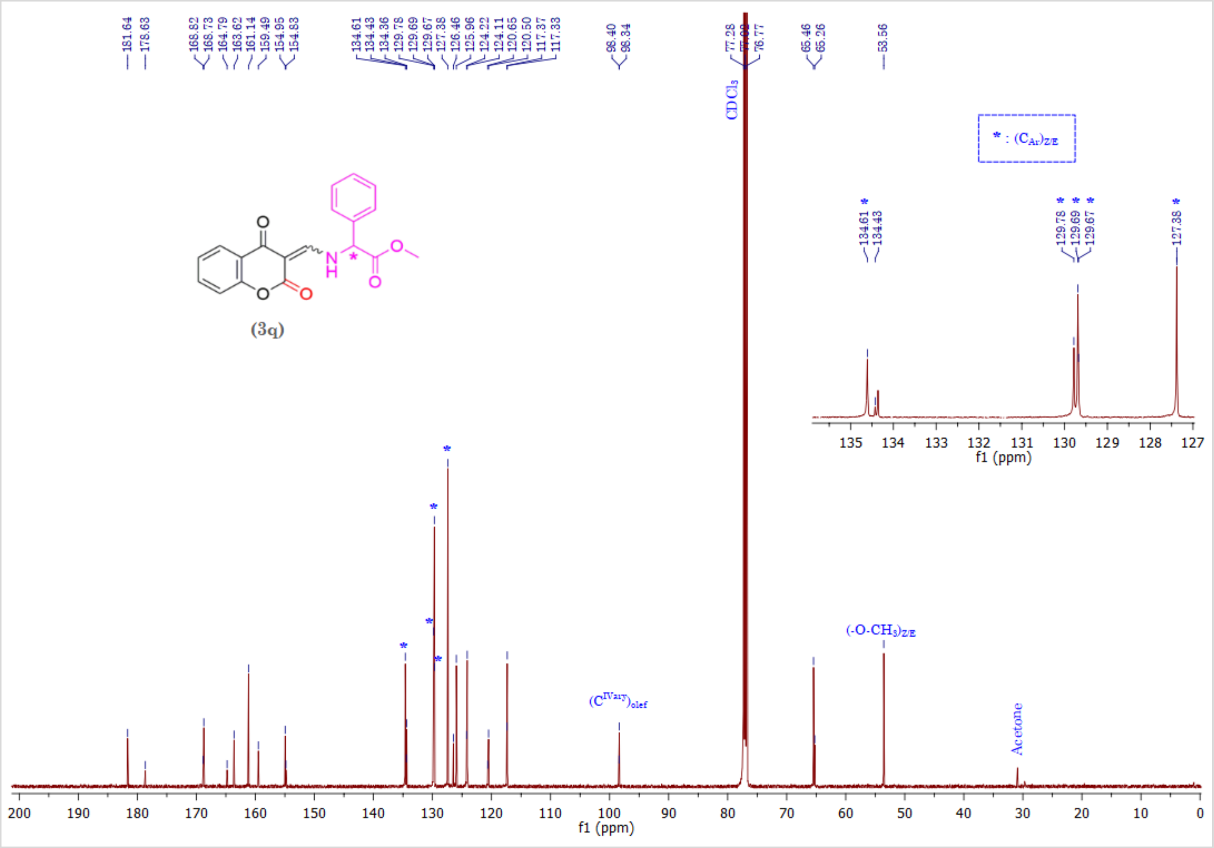

Figure S47. $^{13}C\{^{1}H\}$ NMR spectrum of compound **3q** (125 MHz, $CDCl_3$)

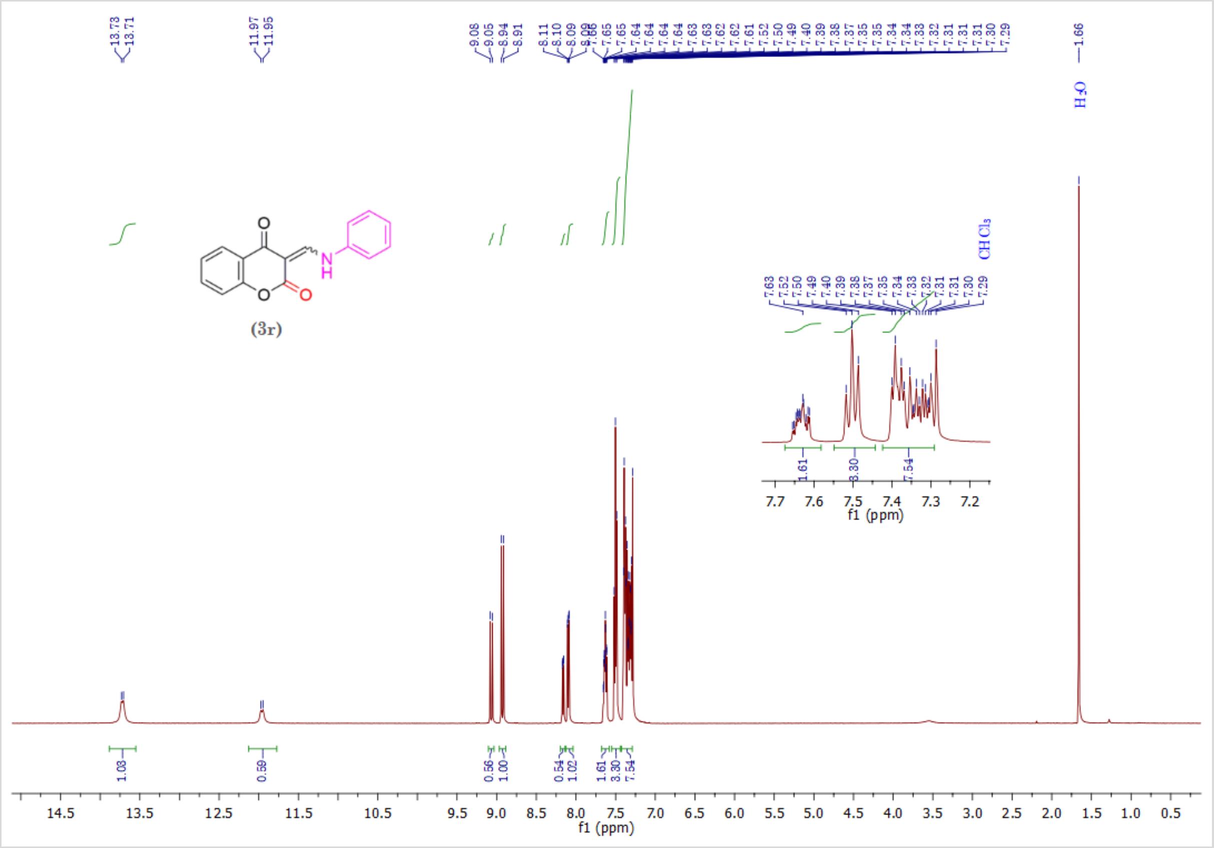

Figure S48. ^{1}H NMR spectrum of compound **3r** (500 MHz, $CDCl_3$)

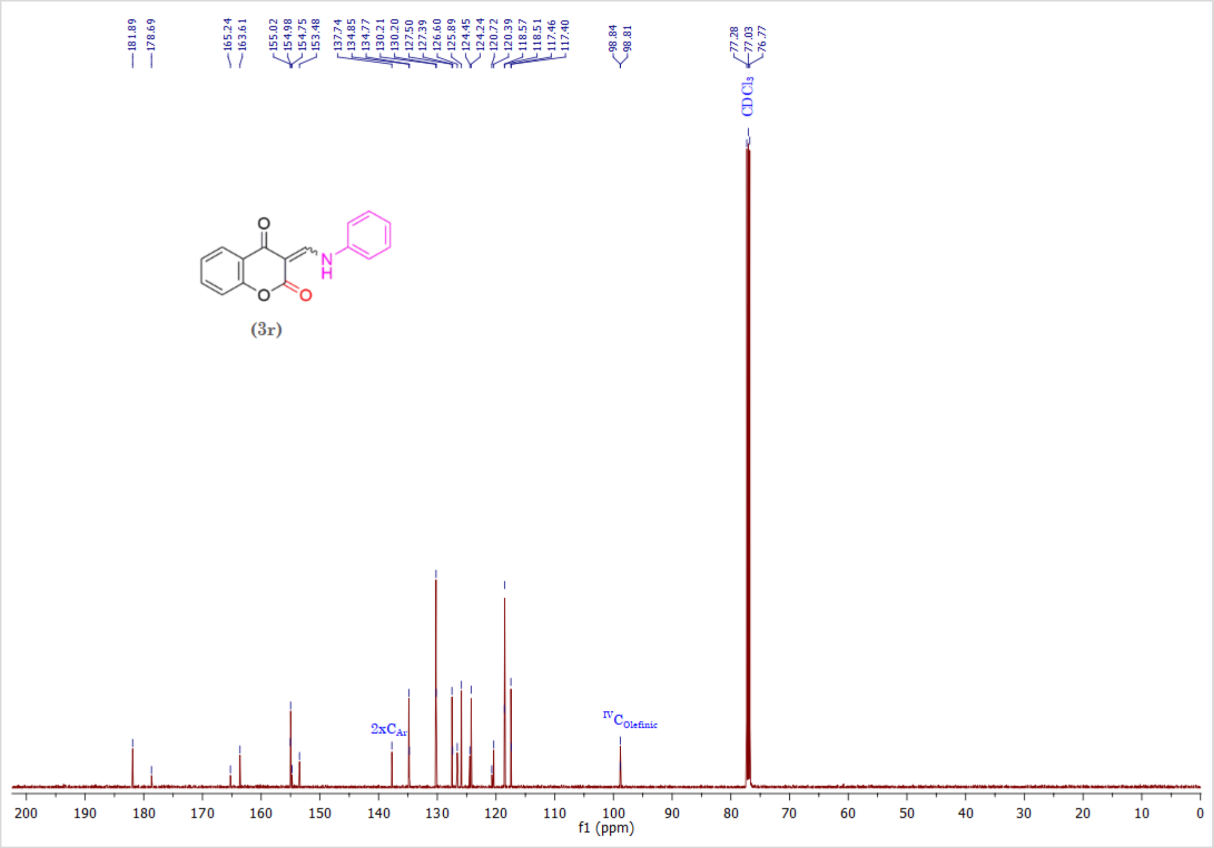

Figure S49. $^{13}C\{^{1}H\}$ NMR spectrum of compound **3r** (125 MHz, $CDCl_3$)

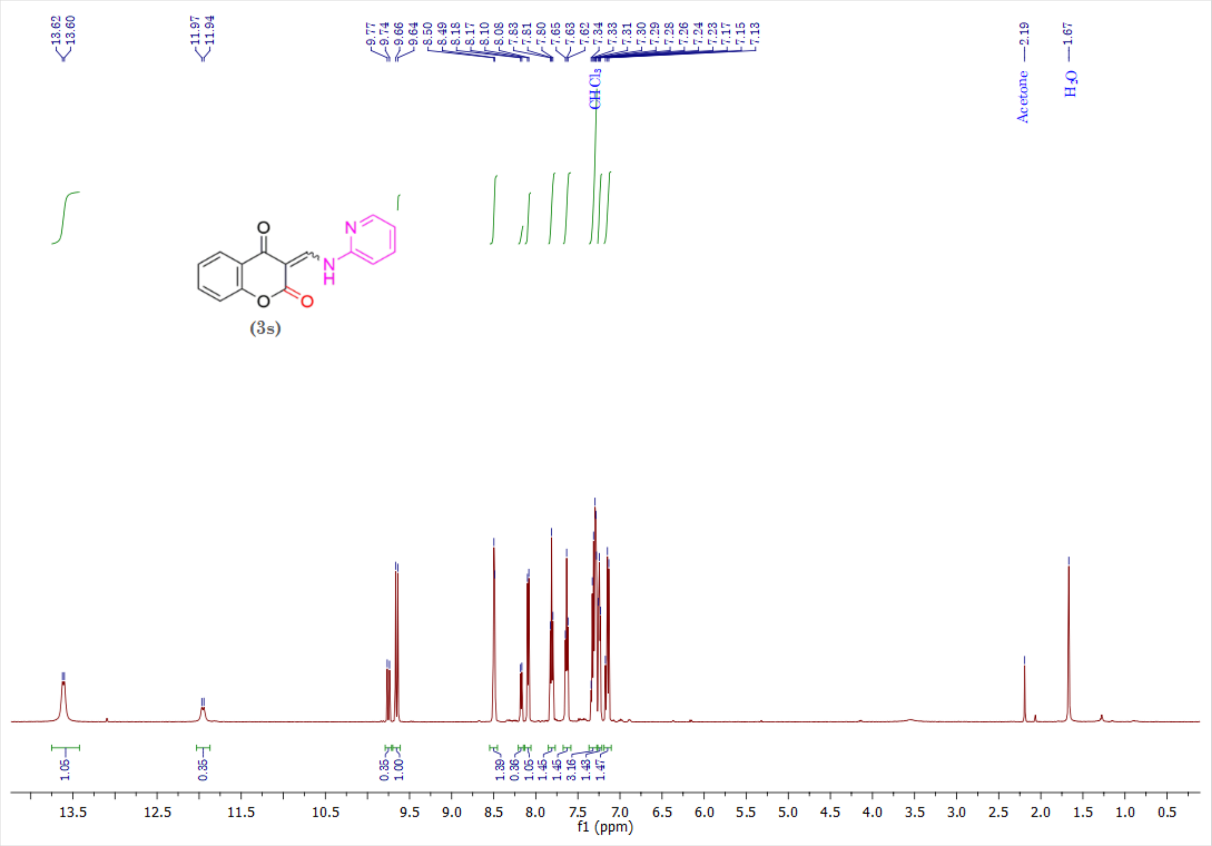

Figure S50. ^{1}H NMR spectrum of compound **3s** (500 MHz, $CDCl_3$)

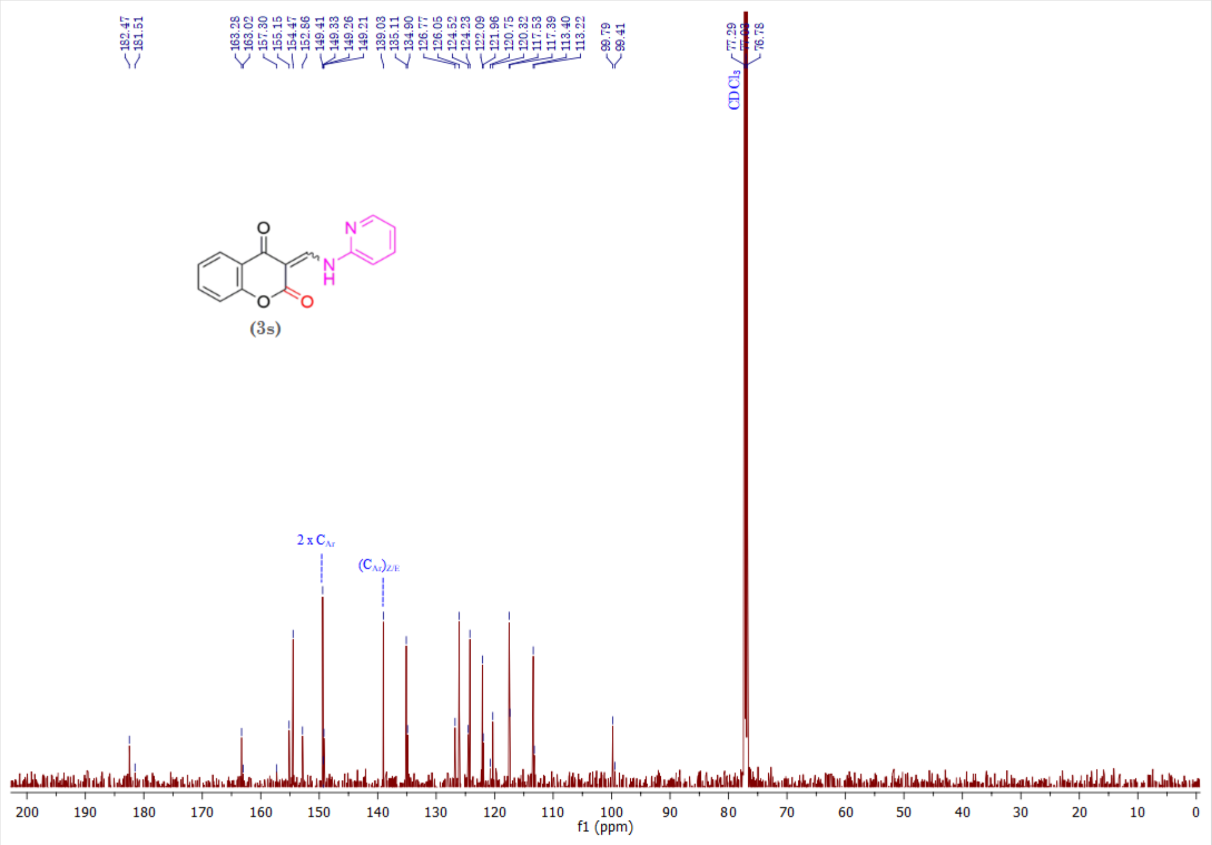

Figure S51. $^{13}C\{^{1}H\}$ NMR spectrum of compound **3s** (125 MHz, $CDCl_3$)

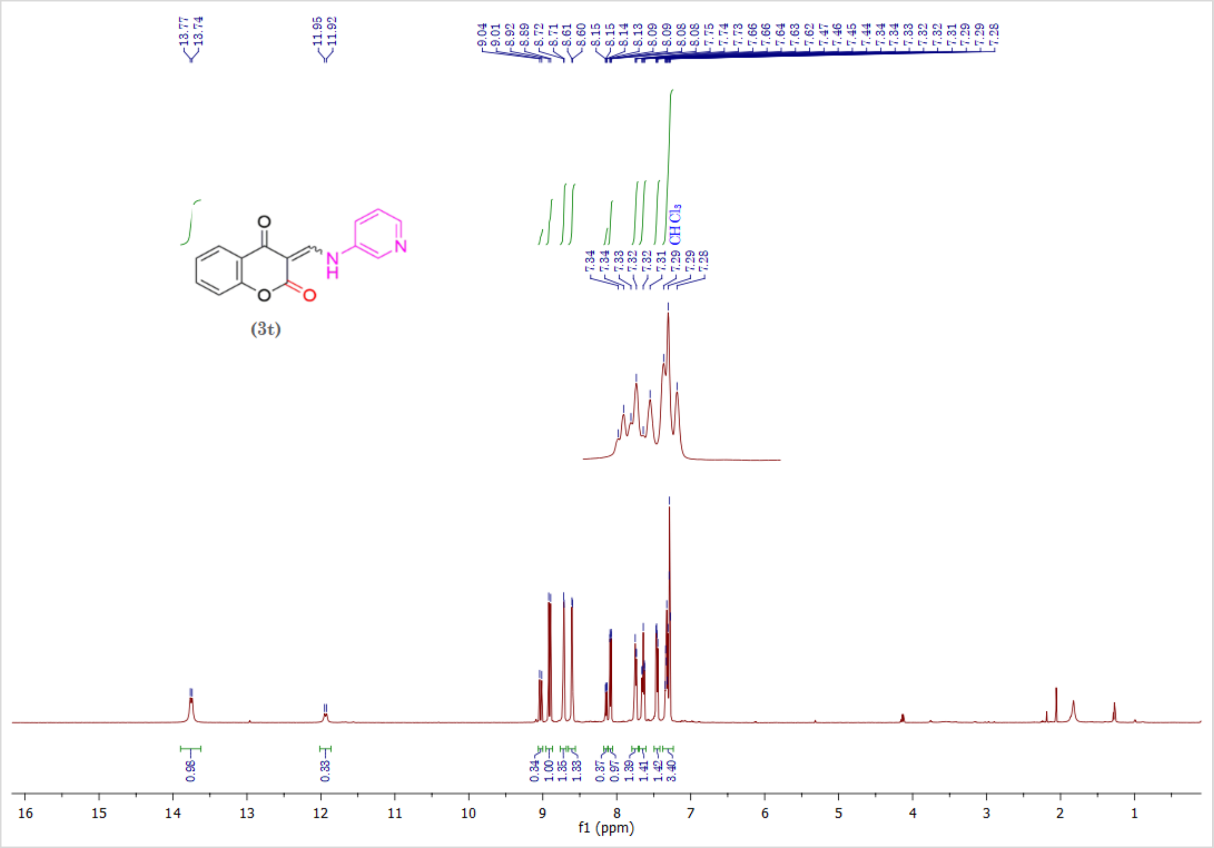

Figure S52. ^{1}H NMR spectrum of compound **3t** (500 MHz, $CDCl_3$)

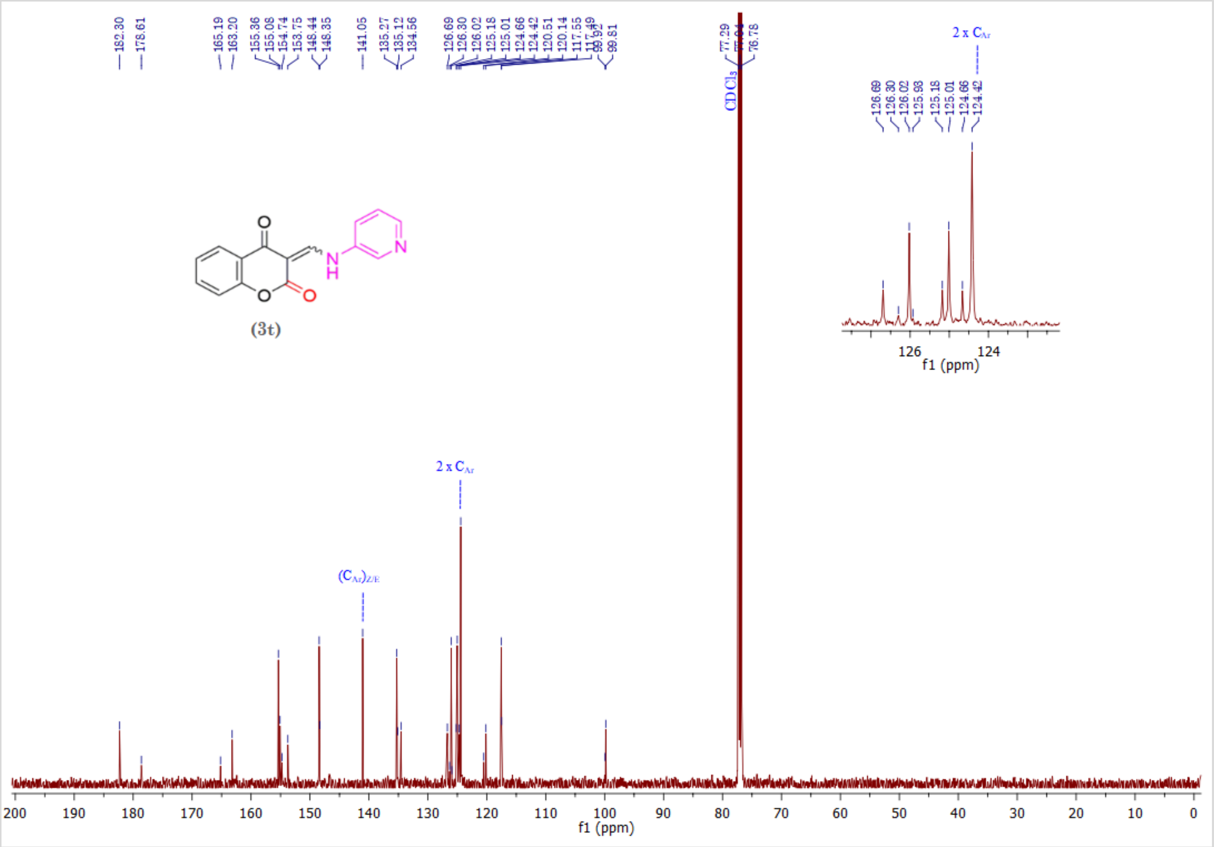

Figure S53. $^{13}C\{^{1}H\}$ NMR spectrum of compound **3t** (125 MHz, $CDCl_3$)

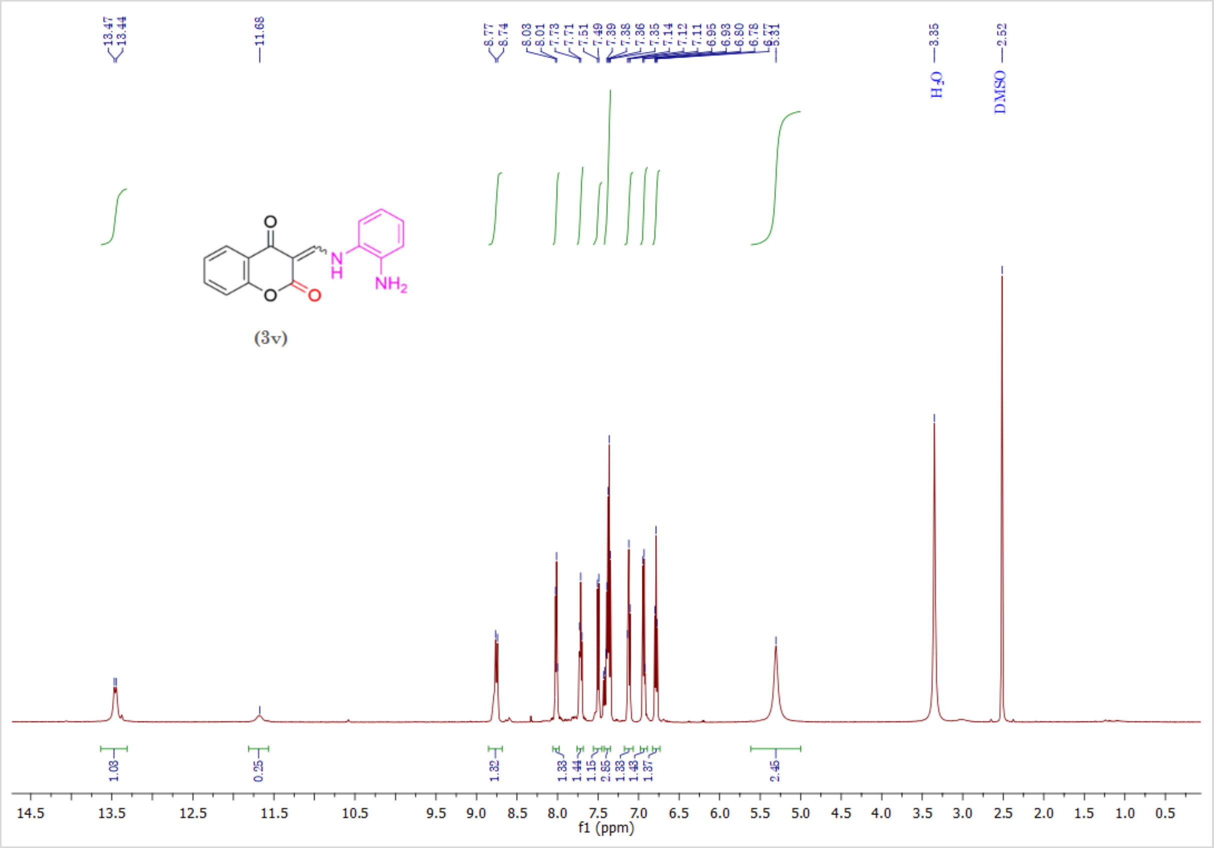

Figure S54. ^{1}H NMR spectrum of compound **3v** (500 MHz, DMSO-d6)

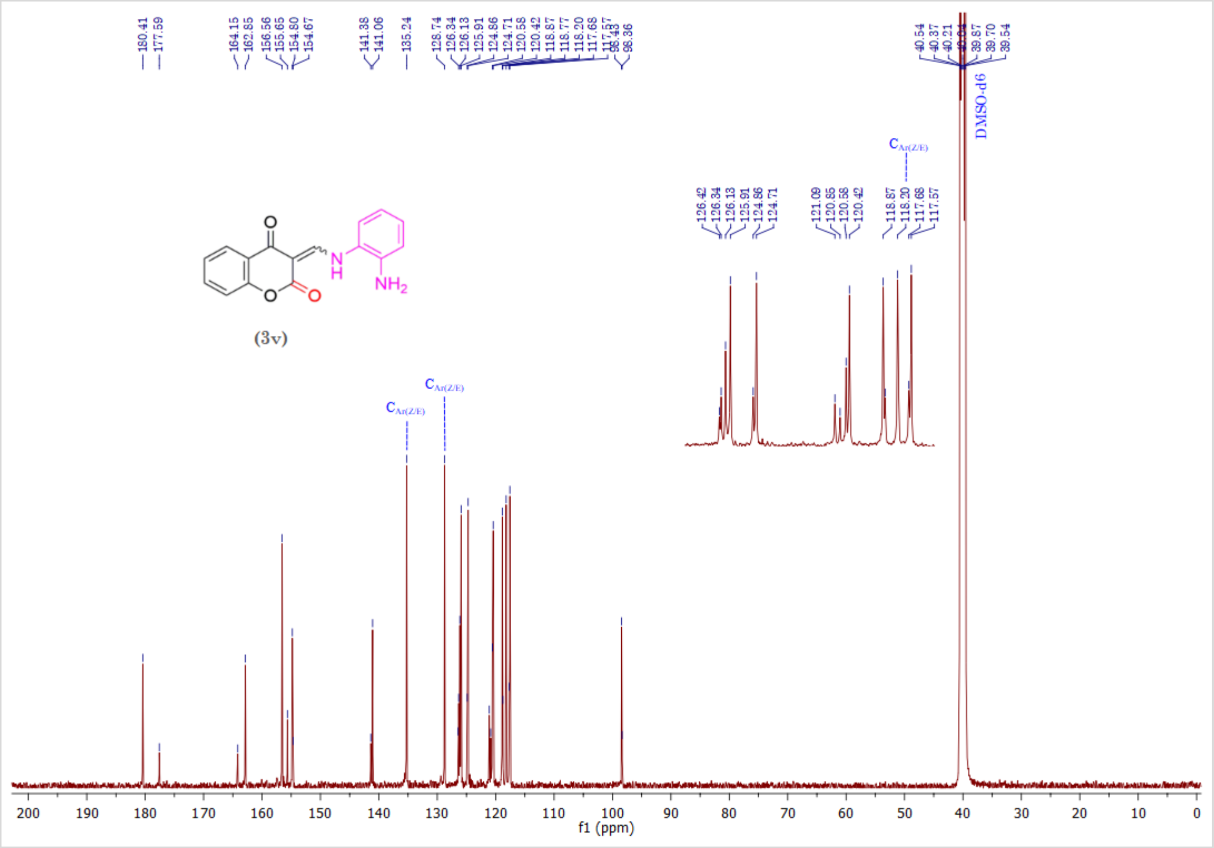

Figure S55. ^{13}C{^{1}H} NMR spectrum of compound **3v** (125 MHz, DMSO-d6)

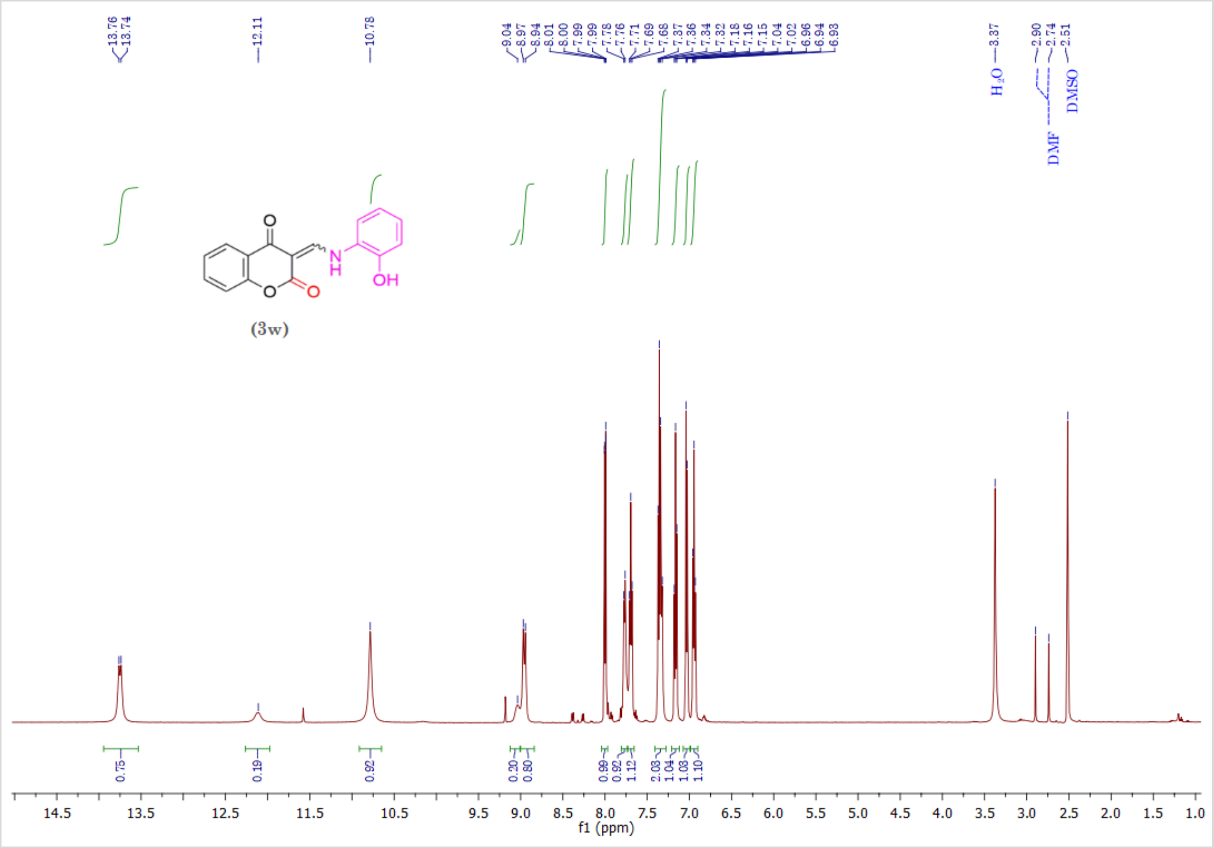

Figure S56. ^{1}H NMR spectrum of compound **3w** (500 MHz, DMSO-d6)

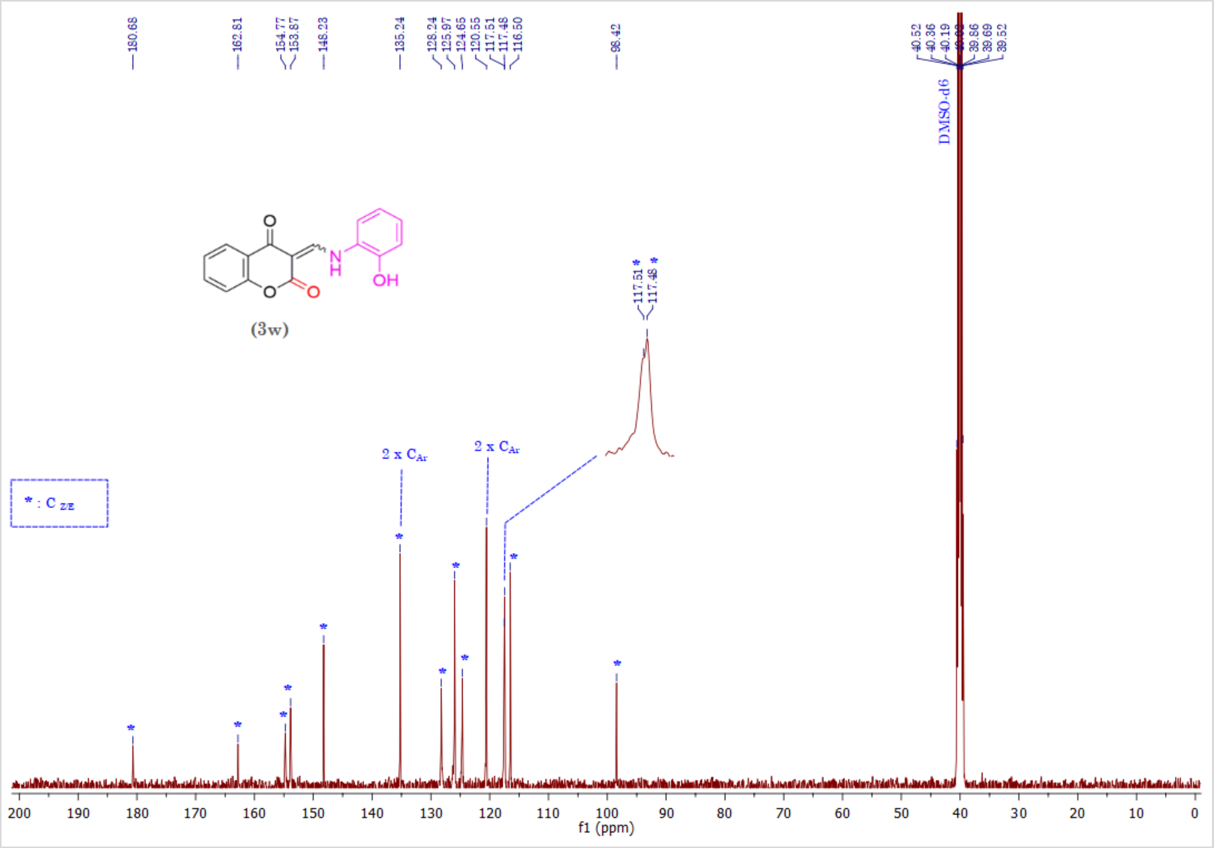

Figure S57. ^{13}C{^{1}H} NMR spectrum of compound **3w** (125 MHz, DMSO-d6)

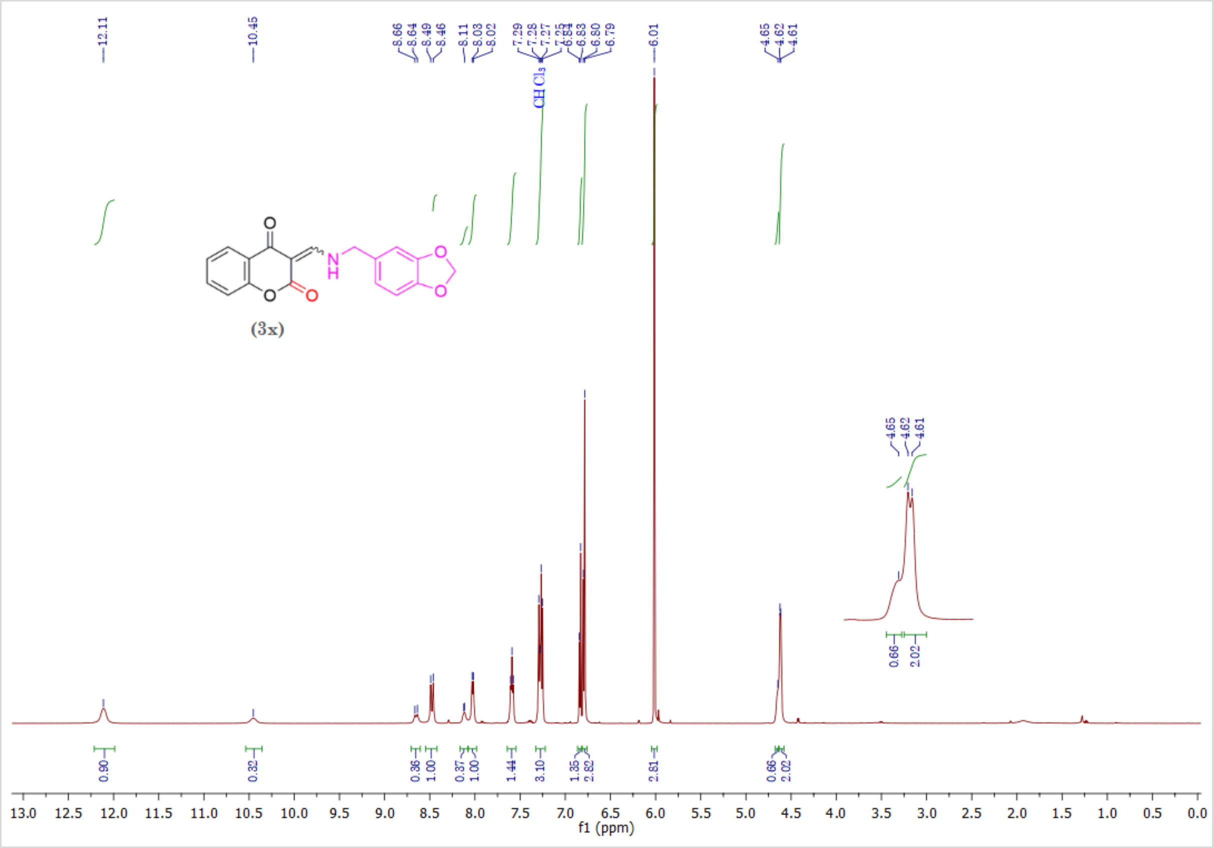

Figure S58. ^{1}H NMR spectrum of compound **3x** (500 MHz, $CDCl_3$)

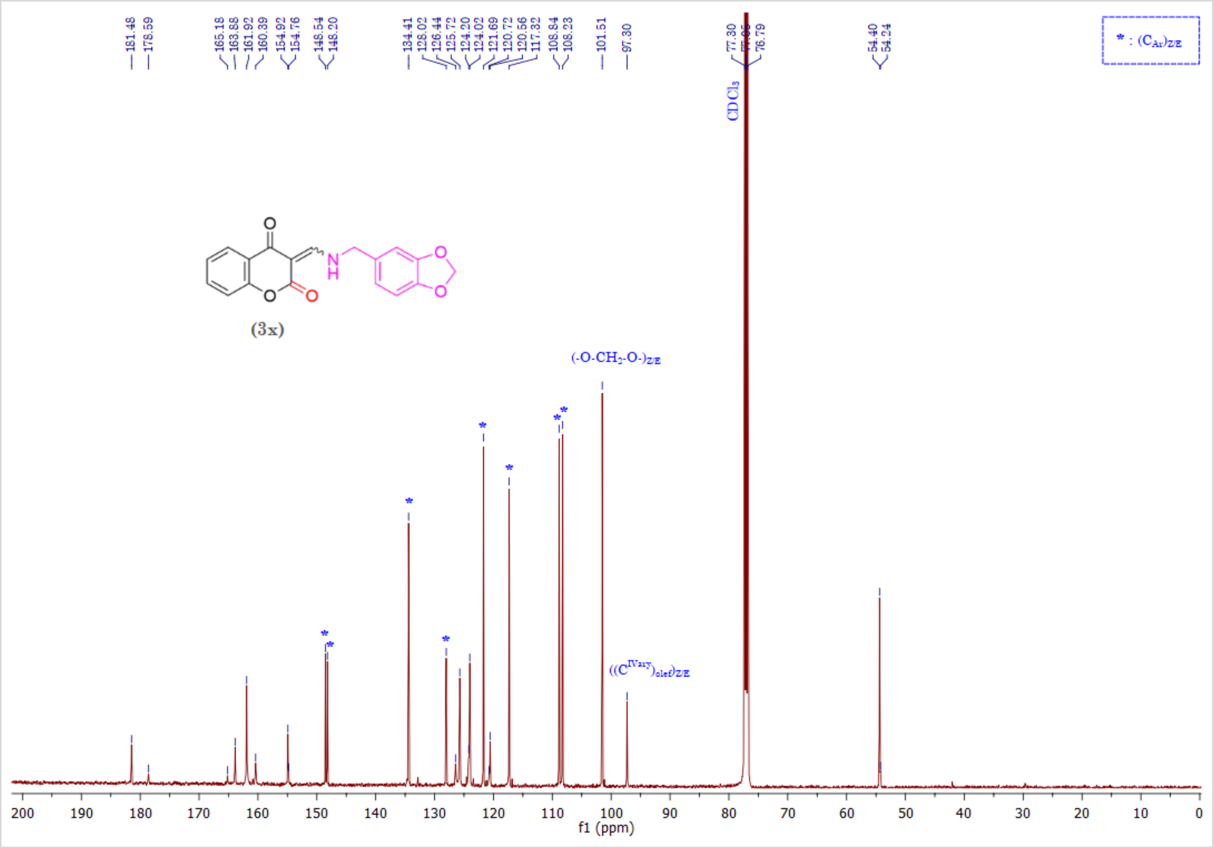

Figure S59. $^{13}C\{^{1}H\}$ NMR spectrum of compound **3x** (125 MHz, $CDCl_3$)

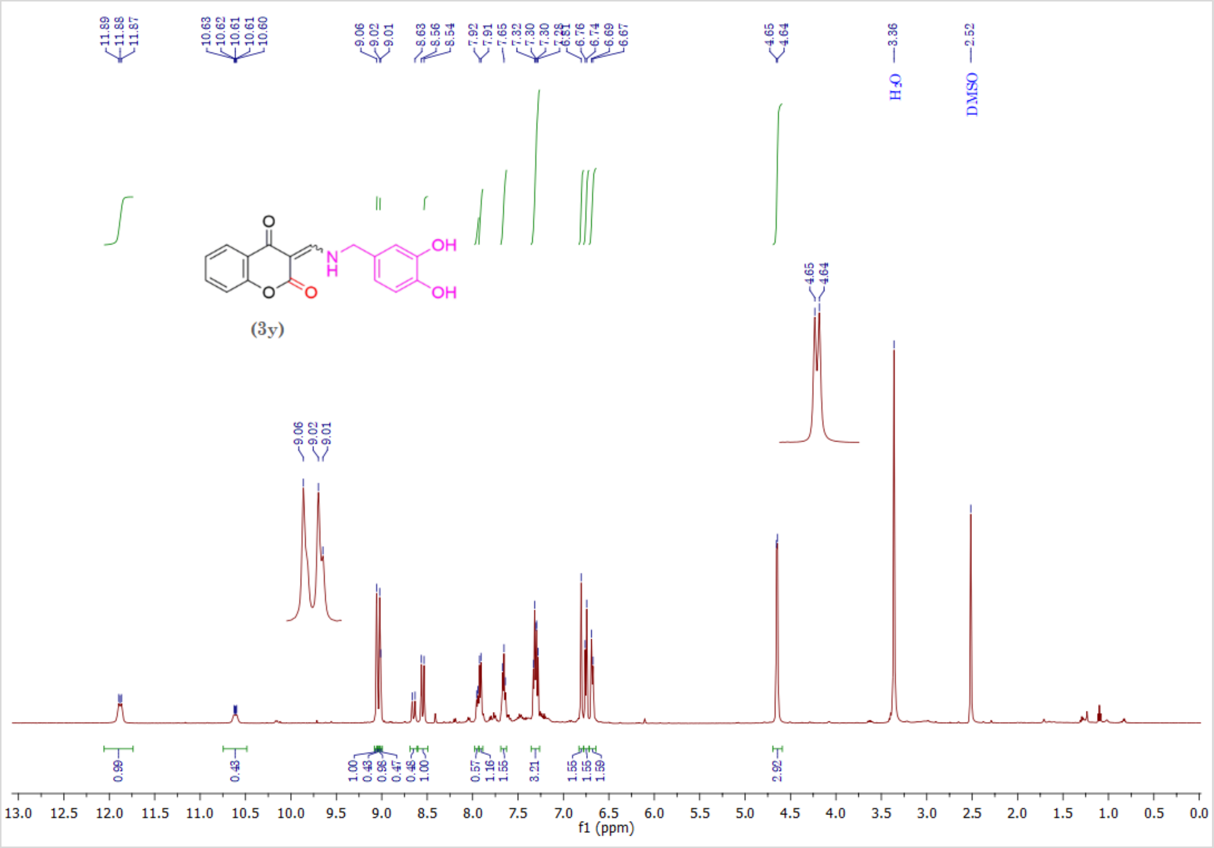

Figure S60. ^{1}H NMR spectrum of compound **3y** (500 MHz, DMSO-d6)

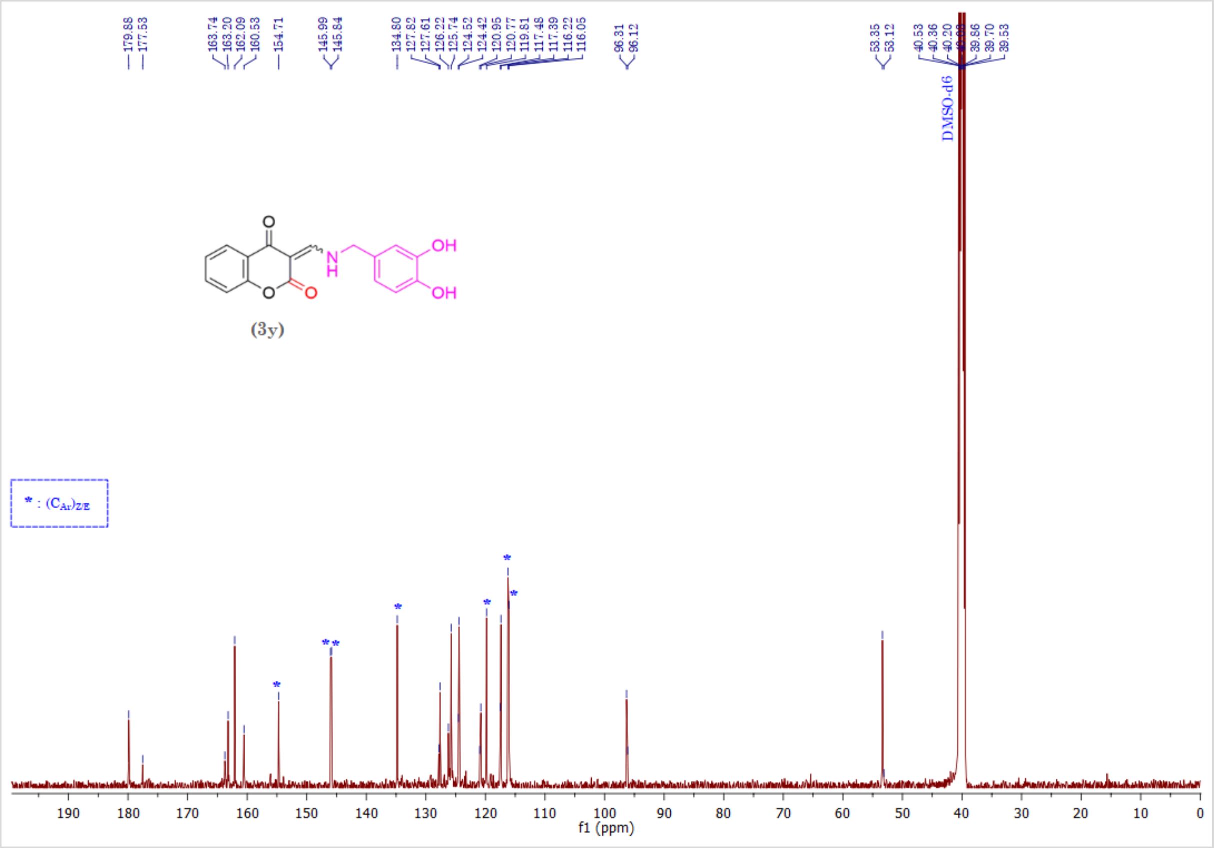

Figure S61. ^{13}C{^{1}H} NMR spectrum of compound **3y** (125 MHz, DMSO-d6)

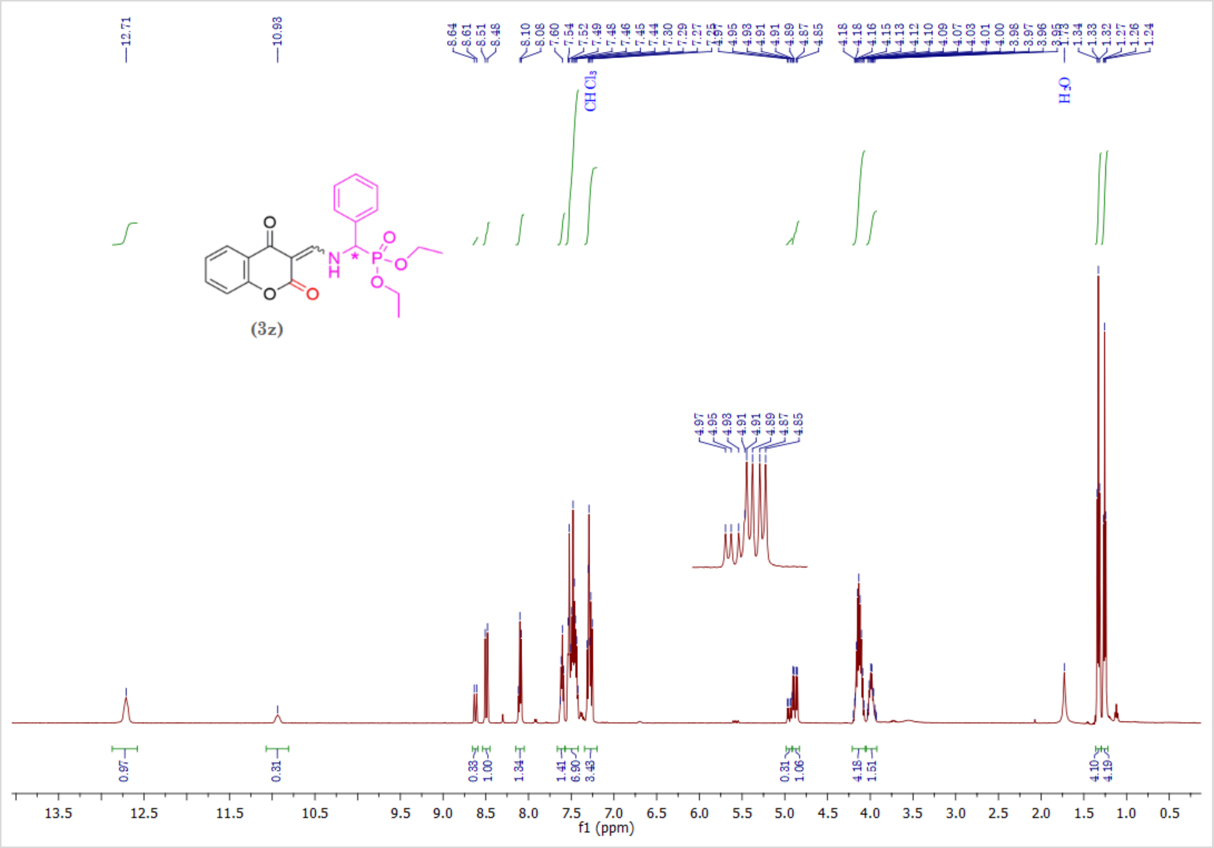

Figure S62. ^{1}H NMR spectrum of compound **3z** (500 MHz, $CDCl_3$)

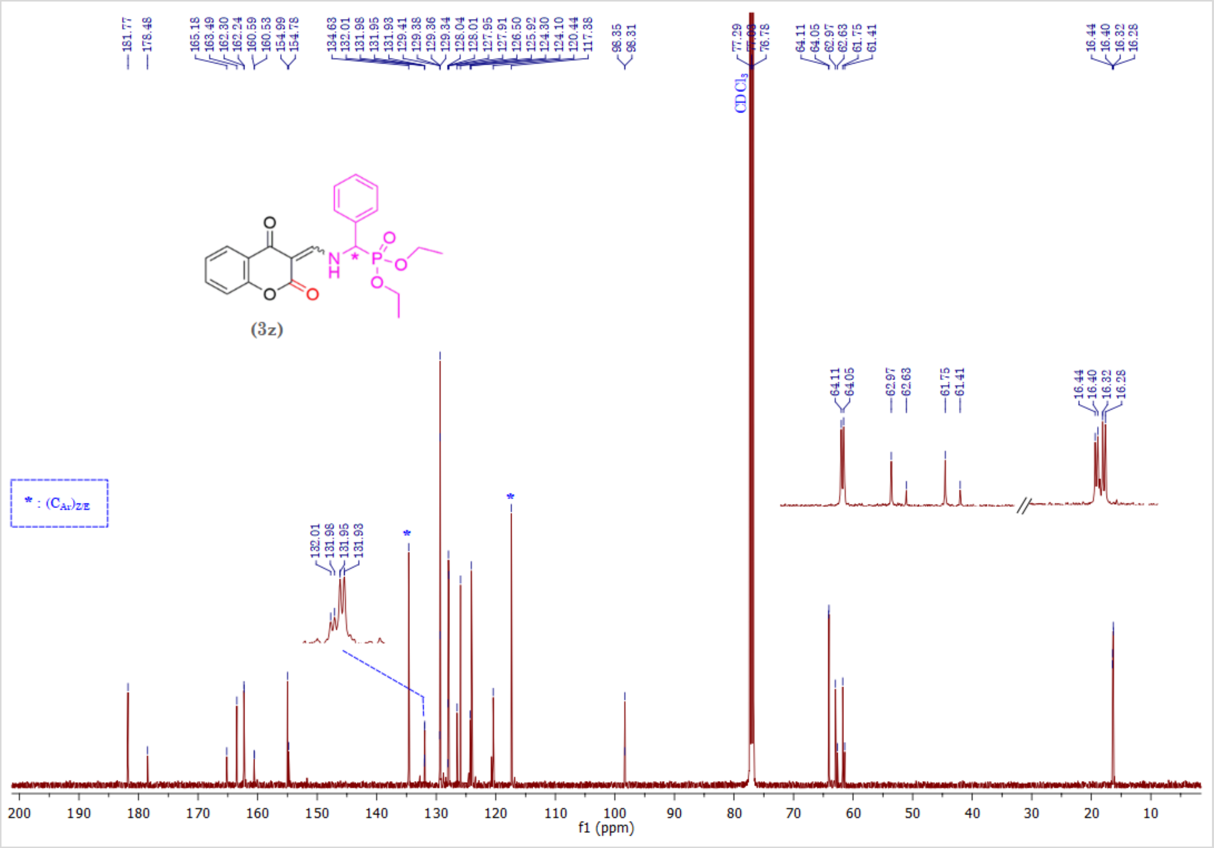

Figure S63. $^{13}C\{^1H\}$ NMR spectrum of compound **3z** (125 MHz, $CDCl_3$)

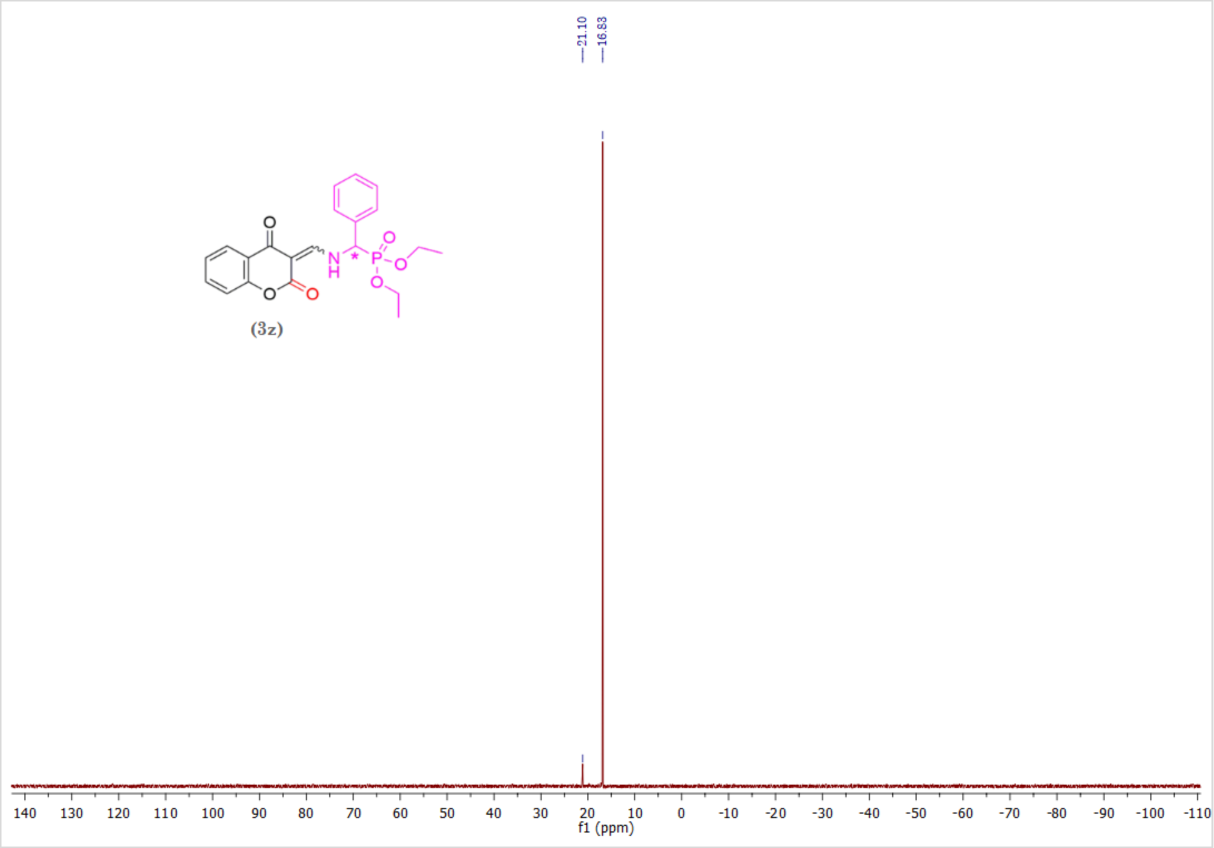

Figure S64. ^{31}P NMR spectrum of compound **3z** (202 MHz, $CDCl_3$)

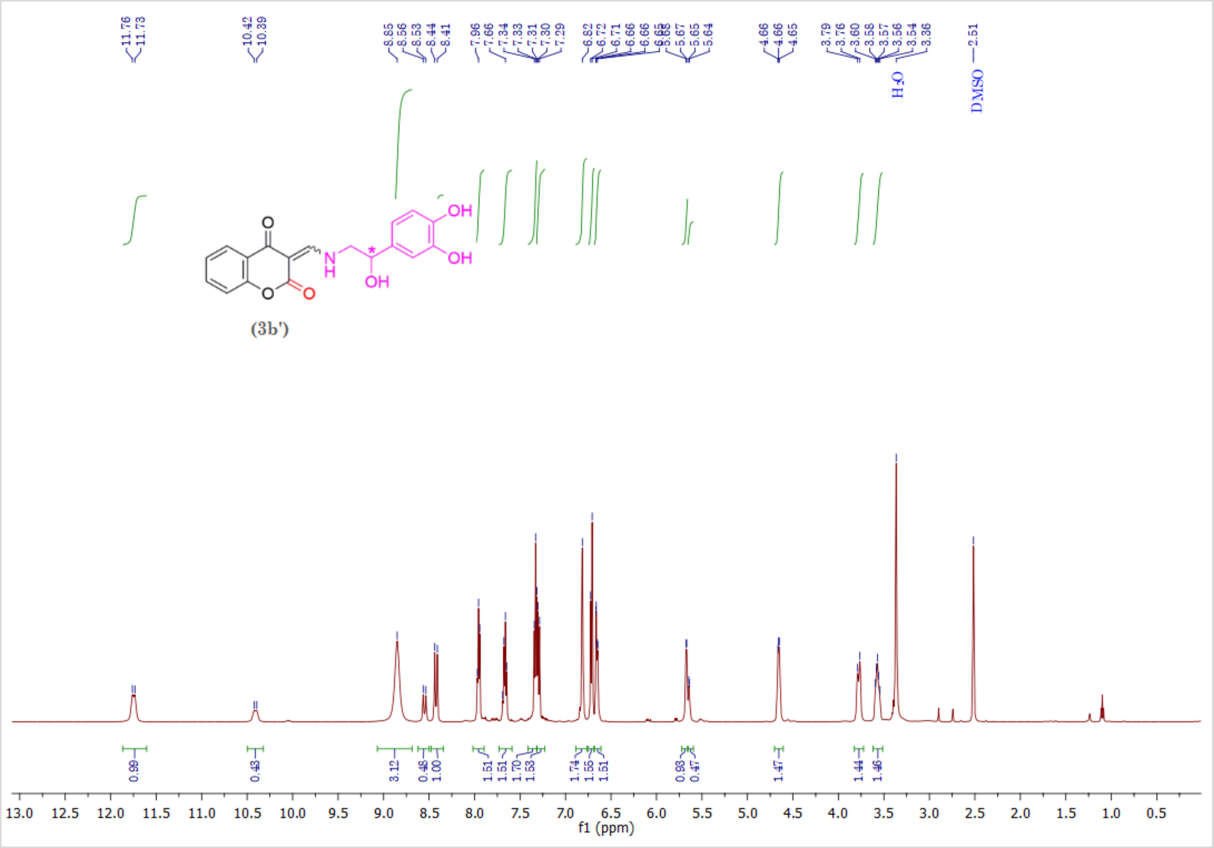

Figure S65. ^{1}H NMR spectrum of compound **3a'** (500 MHz, DMSO-d6)

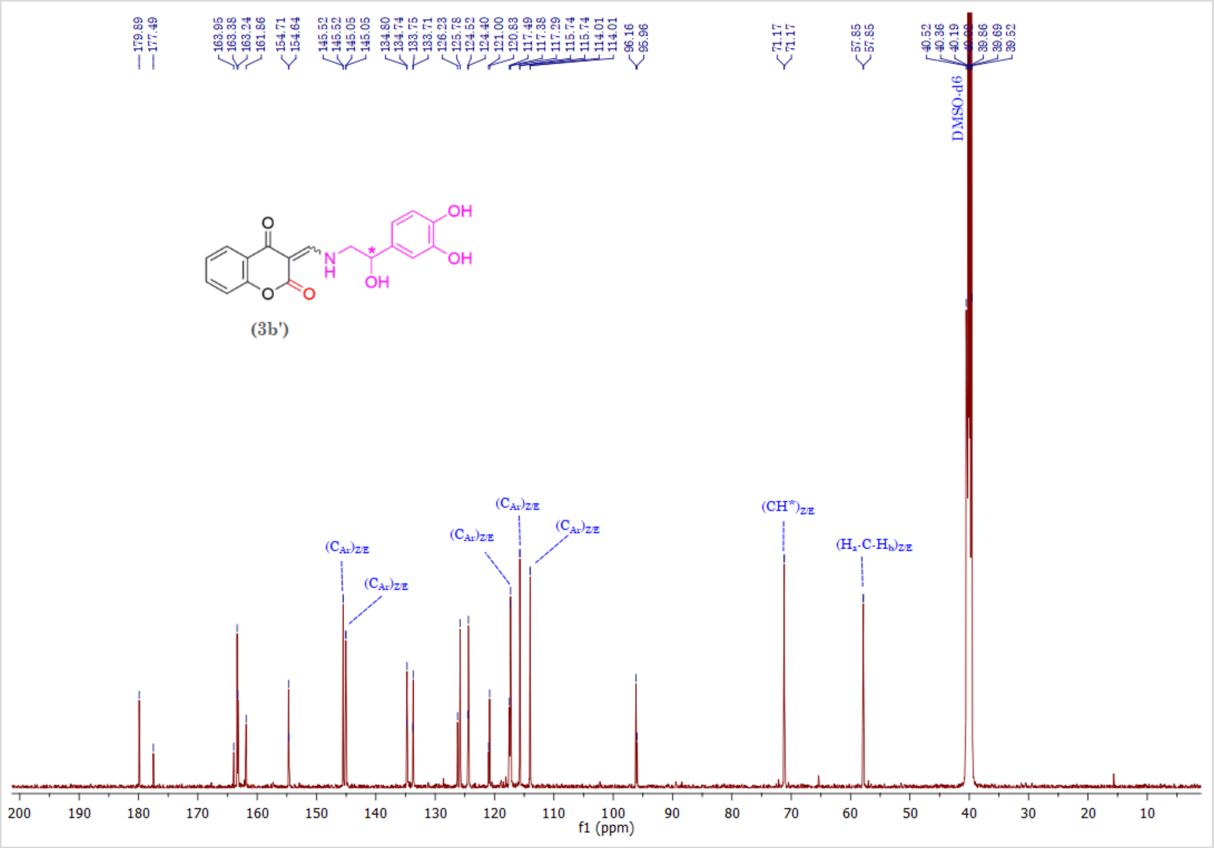

Figure S66. ^{13}C{^{1}H} NMR spectrum of compound **3a'** (125 MHz, DMSO-d6)

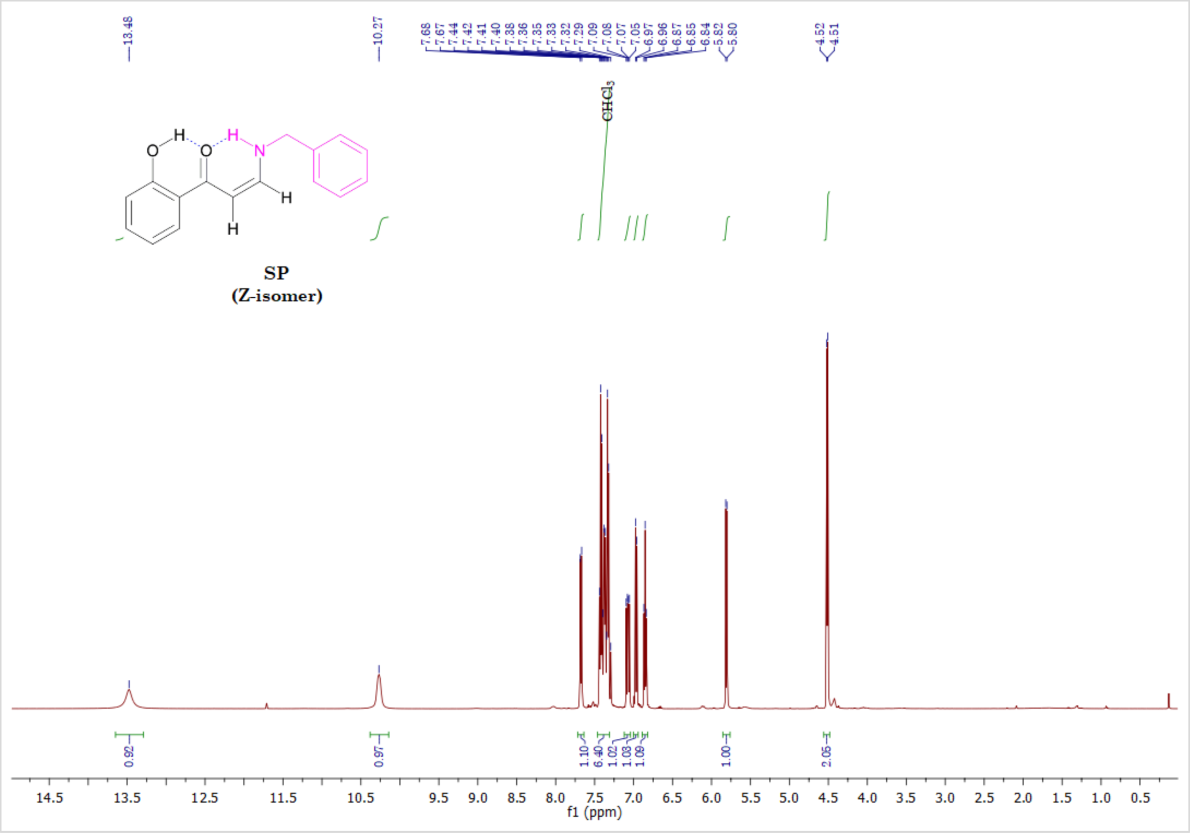

Figure S67. ^{1}H NMR spectrum of ring-opening product **SP** (500 MHz, $CDCl_3$)

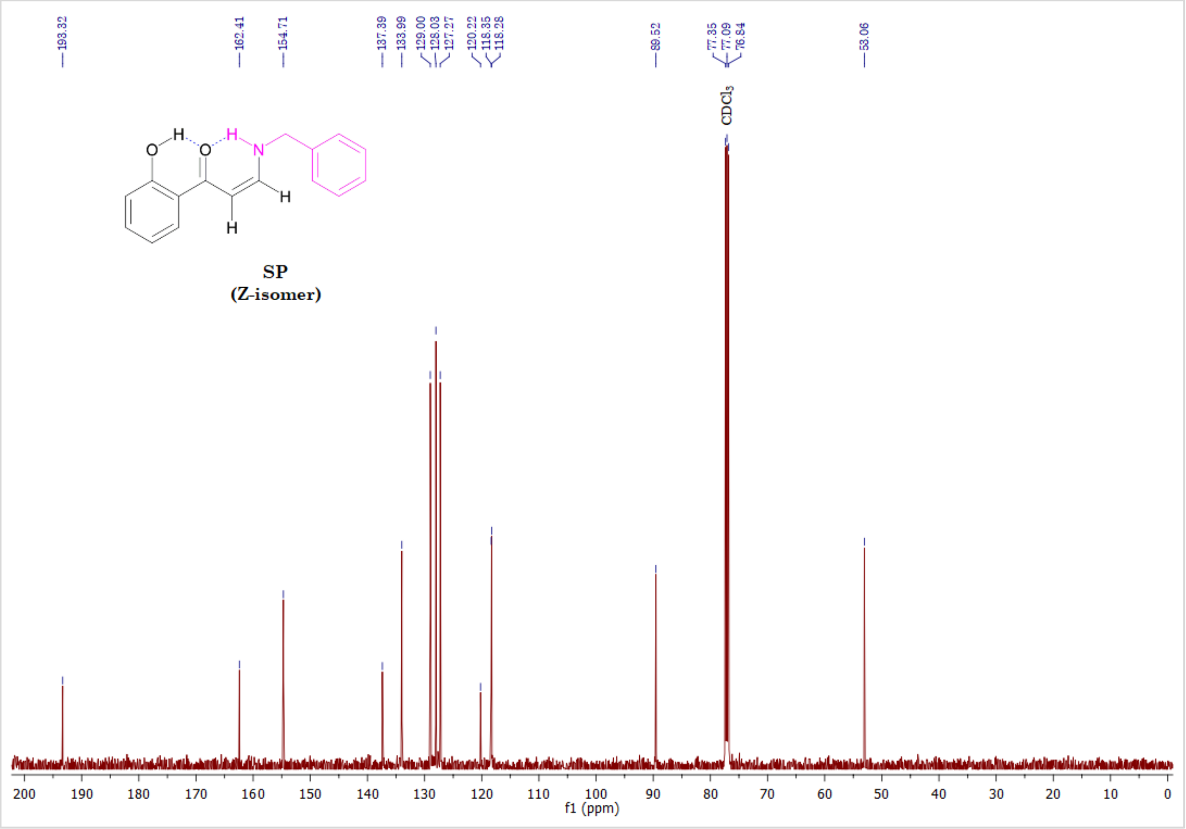

Figure S68. ^{13}C NMR spectrum of ring-opening product **SP** (125 MHz, $CDCl_3$)
